# Investigating Bidirectional Causal Relationships Between Imaging‐Derived Brain Phenotypes and Sedative‐Hypnotic Use Disorder: A Mendelian Randomization Study

**DOI:** 10.1111/adb.70160

**Published:** 2026-05-28

**Authors:** Liqin Lu, Guoxin Zhuang, Jue Deng, Siheng Huang, Liang Meng, Fenglan Li, Xiaoli Zhu

**Affiliations:** ^1^ Department of Forensic Science Fujian Police College Fuzhou China; ^2^ Department of Penalty Execution Fujian Police College Fuzhou China; ^3^ Zhangzhou Drug Rehabilitation Center Zhangzhou Fujian China

**Keywords:** imaging‐derived brain phenotypes, Mendelian randomization, sedative‐hypnotic use disorder

## Abstract

Despite sedative‐hypnotic use disorder (SHUD) being a major public health priority due to its high abuse liability, the underlying causal neurobiological mechanisms of individual susceptibility remain largely unknown. We conducted a large‐scale, bidirectional two‐sample Mendelian randomization (MR) study utilizing genome‐wide association study (GWAS) summary statistics for 3935 brain imaging‐derived phenotypes (IDPs) from the UK Biobank (*N* = 33 000 participants) and SHUD data from the FinnGen R10 release (*N* = 2258 cases). Causal effects were primarily estimated using the inverse‐variance weighted (IVW) method, which was supplemented by a suite of sensitivity analyses including MR‐Egger, weighted median and MR‐PRESSO. Multiple testing was controlled via Benjamini–Hochberg false discovery rate (FDR) correction (q<0.05). Forward MR identified 34 brain IDPs with significant causal associations with SHUD risk. Specifically, increased susceptibility was linked to structural metrics in the temporal and limbic regions, including higher cortical thickness in the left transverse temporal sulcus (OR = 1.35, 95% CI 1.09–1.67, FDR = 0.04) and right middle temporal gyrus (OR = 1.30, 95% CI 1.07–1.58, FDR = 0.04), as well as elevated white‐to‐grey matter intensity contrast in the right insula (OR = 1.31, 95% CI 1.13–1.51, FDR = 0.02) and left parahippocampal gyrus (OR = 1.29, 95% CI 1.08–1.54, FDR = 0.04). Conversely, protective effects were observed for brainstem volume (OR = 0.74, 95% CI 0.61–0.91, FDR = 0.04) and white matter integrity in the left cingulum (OR = 0.73, 95% CI 0.57–0.92, FDR = 0.04). Functional connectivity analyses revealed that higher node activity in the salience network (OR = 0.70, 95% CI 0.53–0.92, FDR = 0.04) and strengthened connectivity between the visual and somatomotor networks (OR range: 0.54–0.74) predicted reduced risk. In contrast, specific edges within the default mode network and visual network were positively associated with SHUD susceptibility (OR = 1.56 and 1.48, respectively). Reverse MR analysis provided limited evidence for a causal effect of genetically predicted SHUD on right middle temporal gyrus thickness. Although a nominal association was observed (OR = 1.03, 95% CI 1.00–1.05, *p* = 0.02), this finding did not survive multiple testing correction (FDR = 0.56) and lacked robustness across sensitivity models. This research establishes a causal link between SHUD susceptibility and specific cortical morphologies, subcortical volumes and functional network topographies, emphasizing the pivotal roles of the temporal–limbic and salience systems. These findings provide novel mechanistic insights into the neurobiological foundations of SHUD while identifying potential imaging biomarkers for risk stratification and the advancement of therapeutic targets.

## Graphical abstract

Using bidirectional two‐sample Mendelian randomisation across 3935 brain imaging‐derived phenotypes, we causally linked specific neuroimaging features to sedative‐hypnotic use disorder (SHUD) susceptibility. Increased cortical thickness in temporal‐limbic regions and altered default mode network connectivity conferred risk, whereas larger brainstem volume, intact cingulum white matter integrity, and heightened salience network activity were protective. These findings implicate the temporal‐limbic and salience systems in SHUD aetiology, providing novel imaging biomarkers and potential therapeutic targets.

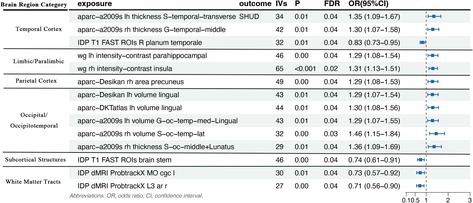

**Abbreviations**
BZDs: benzodiazepines
CI: confidence intervals
GWAS: genome‐wide association study
HR: hazard ratio
IDPs: imaging‐derived phenotypes
IVs: instrumental variables
IVW: inverse‐variance weighted
LD: linkage disequilibrium
MR: Mendelian randomization
MRI: magnetic resonance imaging
MR‐PRESSO: Mendelian Randomization Pleiotropy RESidual Sum and Outlier
OR: odds ratios
SHUD: sedative‐hypnotic use disorder
SNPs: single nucleotide polymorphisms

## 1 Background

Sedative‐hypnotics, particularly benzodiazepines (BZDs) and Z‐drugs, are among the classes of pharmaceuticals with the highest abuse liability and potential for dependence. This phenomenon is largely attributed to their modulatory effects on inhibitory $G{\mathrm{ABA}}_{A}$ receptors in the brain [1, 2]. Clinical evidence suggests that although sedative‐hypnotics effectively treat short‐term insomnia and comorbid conditions such as schizophrenia and depression, their prolonged or off‐label use presents a significant risk of dependence and addiction [3]. According to a 2023 report on controlled psychotropic substances, there has been a steady escalation in both prescription rates and dosages. This trend is accompanied by a rising incidence of emergency department visits and overdose‐related fatalities, particularly those involving BZDs [4]. Collectively, these developments underscore sedative‐hypnotic abuse as a critical public health priority and a significant societal challenge.

Abuse of sedative‐hypnotics often leads to significant tolerance and debilitating withdrawal symptoms, encompassing insomnia, anxiety, cognitive dysfunction and various psychological disturbances [5]. Clinically, drug addiction is conceptualized as a chronic and complex brain disorder involving a network of critical regions, specifically the addiction circuitry, which includes the ventral tegmental area, nucleus accumbens, prefrontal cortex, amygdala and hippocampus [6]. Observational studies have indicated that prolonged or excessive exposure to sedative‐hypnotics is associated with enduring cognitive impairments, particularly deficits in higher‐order cognitive functions [7]. Despite these clinical findings, neuroimaging research into the correlates of sedative‐hypnotic abuse remains sparse. Although non‐invasive magnetic resonance imaging (MRI) has identified connectivity abnormalities within the executive control, default mode and salience networks in various substance use disorders [8, 9, 10, 11], systematic investigations specifically targeting sedative‐hypnotic use disorder (SHUD) remain scarce. Furthermore, due to the limitations of traditional observational designs, such as restricted sample sizes and confounding factors, the causal relationships between imaging‐derived phenotypes (IDPs) and the neurofunctional abnormalities associated with this condition remain elusive.

Mendelian randomization (MR) is a robust epidemiological framework that employs genetic variants as instrumental variables (IVs) to mitigate confounding and circumvent reverse causation, thereby facilitating the evaluation of causal links between exposures and disease outcomes [8]. Previous MR studies have successfully characterized causal relationships between substance abuse and altered brain function [12]. For instance, cannabis use has been causally linked to compromised white matter integrity and aberrant fibre connectivity [13]. However, the causal interplay between SHUD and brain structural and functional characteristics has not been fully elucidated. In the present study, we employed a two‐sample bidirectional MR approach to systematically analyse the causal associations between 3935 brain IDPs and SHUD. This study aims to clarify the potential bidirectional influences between brain morphology and SHUD, providing novel mechanistic insights for the prevention and clinical management of this condition.

## 2 Methods

### 2.1 Data Sources and Study Population

Genome‐wide association study (GWAS) summary statistics for 3935 brain IDPs were obtained from the UK Biobank repository [14]. This dataset includes association results for approximately 17 103 079 single nucleotide polymorphisms (SNPs) across chromosomes 1–22 and the X chromosome, derived from a cohort of approximately 33 000 participants. The brain IDPs analysed in this study encompassed volumetric measures of the temporal cortex, amygdala, brainstem, hippocampus and thalamic subnuclei, alongside Brodmann area FreeSurfer metrics and white matter and grey matter intensity pairs. The UK Biobank study received ethical approval from the Northwest Multicentre Research Ethics Committee (MREC), and all participants provided written informed consent for data acquisition and sharing.

Genetic data for sedative‐hypnotic‐related mental and behavioural disorders were retrieved from the FinnGen study (Release R10; https://r10.risteys.finngen.fi). The FinnGen project integrates genomic data with national health registry information. As of Release R10, the dataset for this phenotype includes approximately 2258 cases. This study utilized publicly available GWAS summary data provided by FinnGen. All participants in the FinnGen study provided informed consent, and the project was conducted in accordance with appropriate ethical standards and approvals.

### 2.2 Genetic Instrument Selection

To ensure the validity of causal inference and minimize potential bias, this study strictly adhered to the three fundamental MR assumptions: (1) the relevance assumption, which requires that genetic variants are robustly associated with the exposure; (2) the independence assumption, stating that variants are independent of potential confounders; and (3) the exclusion restriction, where variants influence the outcome solely through the exposure.

Initially, we screened for genetic variants significantly associated with the exposure. Given the relatively limited number of genome‐wide significant loci for certain brain IDPs, a preliminary significance threshold was set at *p* < 1 × 10^−5^ to ensure sufficient statistical power while maintaining reasonable specificity. When multiple SNPs were in close physical proximity, only the locus with the highest association significance was retained. Subsequently, we employed the clumping algorithm in PLINK (Version 1.9, https://www.cog‐genomics.org/plink/1.9/) to identify independent IVs. Linkage disequilibrium (LD) thresholds were set at $r^{2}$ < 0.05 for brain IDPs and $r^{2}$ < 0.05 for SHUD, utilizing a clumping window of 1 Mb. LD was assessed using the 1000 Genomes Project European (Phase 3) population as the reference panel [15]. Finally, to evaluate instrument strength and avoid weak instrument bias, we calculated the F‐statistic using the formula $F=\beta^{2}/SE^{2}$, where β represents the effect size and SE denotes the standard error. Only SNPs with *F*‐statistic > 10 were included in the final analyses.

### 2.3 Bidirectional Two‐Sample MR Analysis

Bidirectional two‐sample MR analyses were conducted to investigate potential causal associations between brain IDPs and sedative‐hypnotic‐related mental and behavioural disorders. In the forward MR analysis, IDPs were treated as the exposure and sedative‐hypnotic‐related disorders as the outcomes. Conversely, in the reverse MR analysis, sedative‐hypnotic‐related disorders served as the exposure, whereas the IDPs identified as significant in the forward analysis were utilized as outcomes to assess potential reverse causation.

The primary analysis was performed using the inverse‐variance weighted (IVW) method with a multiplicative random‐effects model. To evaluate the robustness of the results, four complementary MR methods were implemented: (1) MR‐Egger regression to provide estimates adjusted for potential directional pleiotropy; (2) the weighted median method, which yields reliable estimates even when up to 50% of the IVs are invalid; (3) the weighted mode method; and (4) the simple mode method, both of which offer supplementary evidence under varying assumptions of instrument validity.

Effect estimates (β) represent the association between genetic variants and the exposure or outcome. For binary outcomes, β coefficients were transformed into odds ratios (OR) with 95% confidence intervals (CI) through exponential transformation. All statistical tests were two‐sided, with nominal significance set at *p* < 0.05. Given the large number of brain IDPs examined in both forward (*n* = 3935) and reverse (*n* = 34) MR analyses, the Benjamini–Hochberg false discovery rate (FDR) correction was applied to control for multiple testing, with *FDR*‐corrected *p*‐values (*q*‐values) < 0.05 considered statistically significant for primary causal inferences [16, 17].

### 2.4 Sensitivity Analysis

To ensure the reliability and stability of the MR results, a comprehensive suite of sensitivity analyses was conducted. Heterogeneity among the IVs was quantified using Cochran's *Q* statistic within both the IVW and MR‐Egger frameworks, with p<0.05 indicating substantial heterogeneity. Potential directional horizontal pleiotropy was assessed via the MR‐Egger regression intercept test; a significant deviation from zero suggested the presence of unbalanced pleiotropy. Furthermore, the Mendelian Randomization Pleiotropy RESidual Sum and Outlier (MR‐PRESSO) global test was implemented to detect potential outlier instruments. When outliers were identified, causal effect estimates were re‐evaluated after removing the offending variants, yielding outlier‐corrected estimates. To further evaluate whether the causal associations were disproportionately driven by any single genetic variant, leave‐one‐out analyses were performed by sequentially omitting each SNP and re‐estimating the effect sizes. Finally, causal inferences were deemed robust if multiple MR methods yielded consistent effect estimates and no substantial violations were detected in heterogeneity or pleiotropy diagnostic tests.

## 3 Results

### 3.1 Baseline Characteristics

The study cohort consisted of *n* = 2258 sedative‐hypnotic users, including 1106 males (49%) and 1152 females (51.0%). The mean age at the initial onset of mental and behavioural disorders was 40.56 years, with means of 41.24 years for males and 39.91 years for females. The age distribution at onset peaked in the 20–30 year (26.3%), 30–40 year (22.7%) and 40–50 year (19.4%) age groups. Lower proportions were observed in the 10–20 years (5.3%) and elderly groups, specifically those aged 60–70 years (7.4%), 70–80 years (4.5%) and ≥ 80 years (1.2%) (Table S1).

During the follow‐up period, sedative‐hypnotic use was significantly associated with a higher risk of various neurological disorders compared to no exposure (Table 1). The most pronounced risk elevations were observed for Alzheimer's disease (HR = 5.29, 95% CI 2.82–12.44) and epilepsy (HR = 5.00, 95% CI 3.41–7.35). Substantial risks were also noted for Parkinson's disease (HR = 2.82, 95% CI 1.29–6.17), migraine with aura (HR = 2.58, 95% CI 1.62–4.12), multiple sclerosis (HR = 2.38, 95% CI 1.28–4.45), migraine (HR = 2.28, 95% CI 1.52–3.41), migraine without aura (HR = 2.26, 95% CI 1.62–4.12) and sleep apnoea (HR = 2.13, 95% CI 1.46–3.11). Regarding psychiatric and behavioural disorders, markedly increased risks were identified for schizophrenia (HR = 18.29, 95% CI 12.46–26.83), bipolar affective disorder (HR = 16.45, 95% CI 11.33–23.9), depression (HR = 7.76, 95% CI 4.77–12.62), vascular dementia (HR = 6.95, 95% CI 2.50–19.36) and dementia (HR = 5.59, 95% CI 2.65–11.78).

**TABLE 1:** Association between sedative‐hypnotic use and the risk of neurological and psychiatric disorders.

Disorder category	Disorder	HR (95% CI)	*p*
Neurological disorders	Alzheimer	5.29 (2.82–12.44)	< 0.001
	Epilepsy	5.00 (3.41–7.35)	< 0.001
	Parkinson's disease	2.82 (1.29–6.17)	< 0.001
	Migraine with aura	2.58 (1.62–4.12)	< 0.001
	Multiple sclerosis	2.38 (1.28–4.45)	< 0.001
	Migraine	2.28 (1.52–3.41)	< 0.001
	Migraine without aura	2.26 (1.62–4.12)	< 0.001
	Sleep apnoea	2.13 (1.46–3.11)	< 0.001
Psychiatric disorders	Schizophrenia	18.29 (12.46–26.83)	< 0.001
	Bipolar affective disorder	16.45 (11.33–23.9)	< 0.001
	Depression	7.76 (4.77–12.62)	< 0.001
	Vascular dementia	6.95 (2.50–19.36)	< 0.001
	Dementia	5.59 (2.65–11.78)	< 0.001

### 3.2 IV Selection

For the forward MR analysis, valid IVs were extracted for each of the 3935 brain IDPs using the following criteria: significant associations between SNPs and exposure (*p* < 10^−5^), LD clumping ($r^{2}$ < 0.01 with a window size of 1 Mb) and the exclusion of weak instruments (defined as an *F*‐statistic < 10). This process yielded between 8 to 103 valid IVs per brain IDP for MR analysis (Data S1). For the reverse MR analysis, applying screening criteria of *p* < 10^−5^, $r^{2}$ < 0.05 and a window size of 1 Mb while retaining only instruments with an *F*‐statistic > 10 yielded 31 IVs significantly associated with sedative‐hypnotic‐related disorder (Table S2).

### 3.3 Forward MR: The Putative Causal Effects of Brain IDPs on SHUD

In the forward MR analysis, we used brain IDPs as exposures and SHUD as the outcome. Among 3935 IDPs evaluated, 177 were successfully matched with sufficient valid IVs, ranging from 12 to 75 SNPs per phenotype (Data S2), for MR analysis. After FDR correction (*q* < 0.05), 34 brain IDPs demonstrated significant causal associations with SHUD. Among these, 14 structural IDPs were mapped to specific anatomical regions, including the temporal, parietal and occipital cortices, the brainstem, the insula, the limbic system and various white matter tracts. The remaining 20 significant IDPs were functional connectivity measures distributed across multiple networks, including the salience, visual, default mode, auditory and somatomotor networks.

**FIGURE 1:**
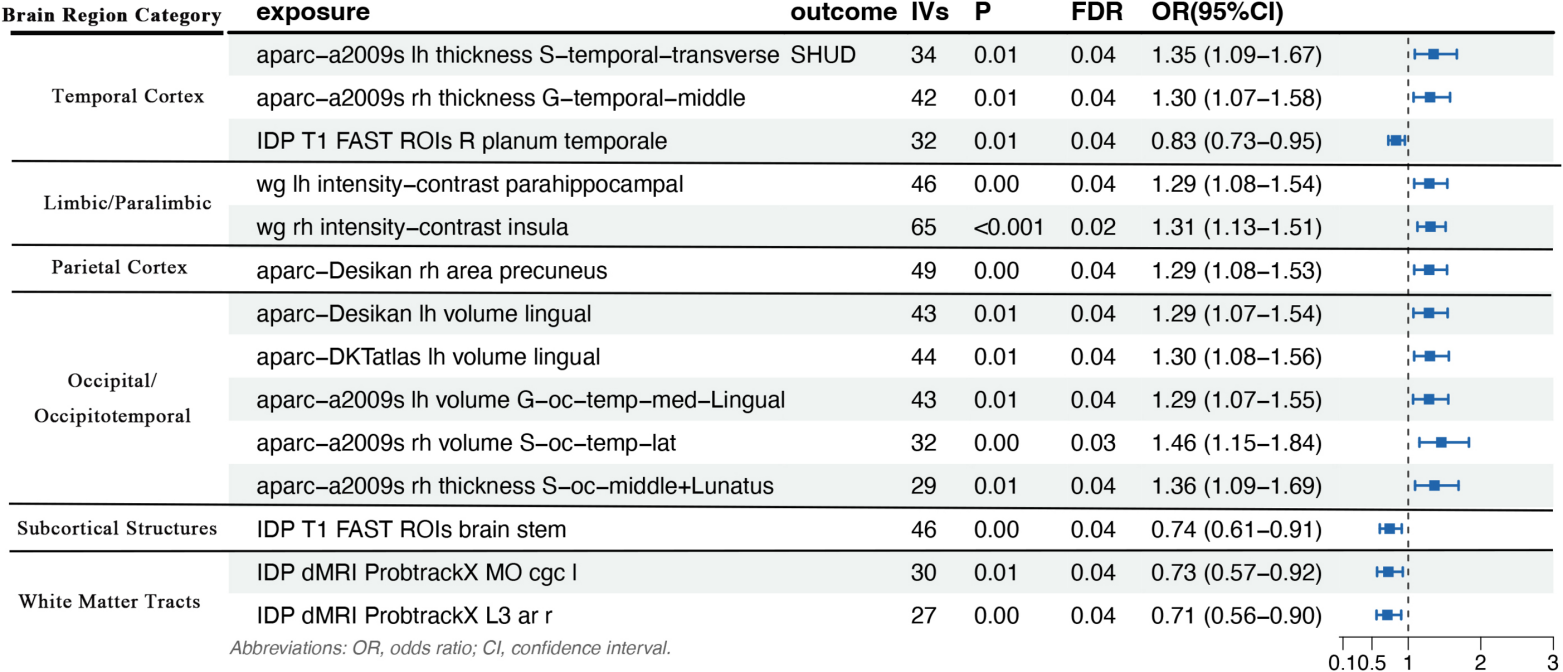
Associations between brain imaging–derived phenotypes (IDPs) and the risk of SHUD. Forest plot presenting the ORs and 95% CIs for IDPs significantly associated with SHUD risk (FDR < 0.05). Features are categorized into cortical regions (temporal, limbic, parietal and occipitotemporal), subcortical structures and white matter tracts. The vertical dashed line indicates OR = 1; points to the left/right represent protective/risk associations per standard deviation (SD) increase in IDP values. CI, confidence interval; FDR, false discovery rate; OR, odds ratio; SD, standard deviation.

Results demonstrated that cortical alterations in the temporal cortex were significantly associated with SHUD risk, with all findings surviving FDR correction (Figure 1). Specifically, two cortical thickness metrics showed positive associations: Each standard deviation (SD) increase in the thickness of the left transverse temporal sulcus (lh S‐temporal‐transverse) was associated with a 35% increased risk of SHUD (OR = 1.35, 95% CI 1.09–1.67, *p* = 0.01, FDR = 0.04). Similarly, each SD increase in the right middle temporal gyrus (rh G‐temporal‐middle) thickness was linked to a 30% increase in risk (OR = 1.30, 95% CI 1.07–1.58, *p* = 0.01, FDR = 0.04). Conversely, a negative association was identified for the volume of the right planum temporale, where each SD increase correlated with a 17% reduction in SHUD risk (OR = 0.83, 95% CI 0.73–0.95, *p* = 0.01, FDR = 0.04).

Within the limbic and paralimbic regions, the white‐to‐grey matter signal intensity contrast (WG contrast) was positively associated with SHUD risk. A 1‐SD increase in the WG contrast of the left parahippocampal gyrus (wg lh intensity‐contrast parahippocampal) was associated with a 29% increase in the odds of SHUD (OR = 1.29, 95% CI 1.08–1.54, *p* = 0.00, FDR = 0.04). Furthermore, higher WG contrast in the right insula (wg rh intensity‐contrast insula) was linked to a 31% higher risk (OR = 1.31, 95% CI 1.13–1.51, *p* < 0.001, FDR = 0.02), highlighting the potential involvement of limbic structural integrity in SHUD susceptibility.

In the parietal and occipitotemporal regions, associations remained robust across multiple anatomical atlases. In the parietal cortex, a greater surface area of the right precuneus (Desikan atlas) was associated with an increased risk of SHUD (OR = 1.29, 95% CI 1.08–1.53, *p* = 0.00, FDR = 0.04). Findings regarding the lingual gyrus were particularly consistent; an increased volume in this region was identified as a significant risk factor across the Desikan (left: OR = 1.29, FDR = 0.04), DKT (left: OR = 1.30, FDR = 0.04) and a2009s (right: OR = 1.46, FDR = 0.03; left medial occipitotemporal/lingual gyrus: OR = 1.29, FDR = 0.04) parcellations. Additionally, increased cortical thickness in the right middle occipital and lunatus sulcus (rh S‐oc‐middle + Lunatus) was positively linked to SHUD risk (OR = 1.36, 95% CI 1.09–1.69, *p* = 0.01, FDR = 0.04).

In contrast, protective effects were observed in subcortical structures and white matter tracts. For subcortical regions, a 1‐SD increase in brainstem volume was associated with a 26% reduction in SHUD risk (OR = 0.74, 95% CI 0.61–0.91, *p* = 0.00, FDR = 0.04). Regarding white matter microstructure, the mode of anisotropy (MO) in the left cingulum cingulate gyrus (cgc L) and the third eigenvalue (L3) of the right acoustic radiation (ar R) were both negatively correlated with SHUD risk (OR = 0.73, FDR = 0.04; and OR = 0.71, FDR = 0.04, respectively).

**FIGURE 2:**
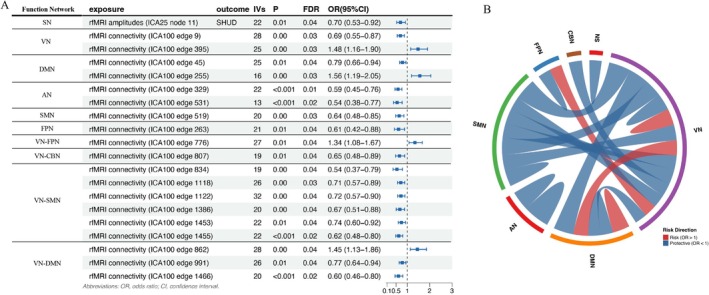
Brain network associations with SHUD risk. (A) Forest plot displaying the ORs and 95% CIs for specific within‐network nodes (amplitude) and connectivity edges (functional connectivity) significantly associated with SHUD risk. The vertical dashed line indicates the null effect (OR = 1.0). Points to the right of the line represent increased risk, whereas points to the left indicate protective effects. All displayed associations survived FDR correction (FDR < 0.05). (B) Chord diagram visualizing the topographical landscape of association in (A). Arcs represent functional networks: AN, auditory; CBN, cerebellar; DMN, default mode; FPN, frontoparietal; SMN, somatomotor; VN, visual. Ribbons denote significant connectivity, with colour indicating risk direction (red: OR > 1; blue: OR < 1) and width weighted by statistical significance (−log_10_ *p*‐value).

The functional brain network architecture, including within‐network activity and between‐network connectivity, was significantly associated with the risk of SHUD after FDR correction (Figure 2A,B). Within‐network associations were identified across several primary systems. Regarding node activity, higher rfMRI amplitude in the salience network (SN) (ICA25 node 11) was associated with a reduced risk of SHUD (OR = 0.70, 95% CI 0.53–0.92, FDR = 0.04). Within the auditory network (AN), two connectivity edges (ICA100 edges 329 and 531) exhibited strong protective effects (both *p* < 0.001, FDR < 0.05). For the visual network (VN) and default mode network (DMN), findings were bidirectional: Specific edges showed reduced risk (VN edge 9: OR = 0.69; DMN edge 45: OR = 0.79), whereas others were linked to elevated risk (VN edge 395: OR = 1.48; DMN edge 255: OR = 1.56). Additionally, protective associations were observed for connectivity within the somatomotor network (SMN) (edge 519: OR = 0.64) and the frontoparietal network (FPN) (edge 263: OR = 0.61).

Between‐network functional connectivity also revealed extensive associations with SHUD risk, particularly involving the VN. Numerous connections between the VN and SMN consistently demonstrated protective effects, including edges 834, 1118, 1122, 1386, 1453 and 1455 (ORs ranging from 0.54 to 0.74, all FDR < 0.05). Interactions between the VN and DMN were also significant, with edge 862 linked to increased risk (OR = 1.45), but edges 991 and 1466 showed protective associations. Furthermore, SHUD risk was positively associated with VN‐FPN connectivity (edge 776: OR = 1.34) and negatively associated with VN‐CBN (cerebellar) connectivity (edge 807: OR = 0.65).

### 3.4 Reverse MR: Causal Effects of Sedative‐Hypnotic Use Disorder on Brain IDPs

**FIGURE 3:**
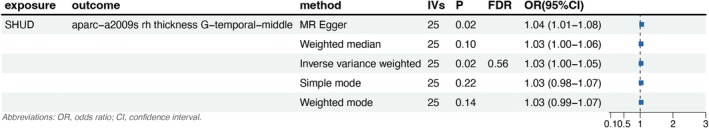
Forest plot of estimated causal effects in the reverse MR. The plot illustrates associations reaching nominal significance (*p* < 0.05). Data are presented as odds ratios (ORs) with error bars representing 95% confidence intervals (CIs). *Note:* None of the depicted associations survived multiple testing correction (FDR = 0.56).

To investigate potential reverse causality, we conducted a reverse MR analysis using SHUD as the exposure and the 34 previously identified IDPs as outcomes. Among the five MR methods employed, significant associations were exclusively identified using the IVW method (Figure 3). Specifically, genetically predicted SHUD showed a nominal association with increased cortical thickness in the right middle temporal gyrus (rMTG) (OR = 1.03, 95% CI 1.00–1.05, *p* = 0.02). Notably, this finding did not survive multiple testing correction (FDR = 0.56) and lacked consistency across alternative MR sensitivity models such as MR‐Egger and weighted median. These results suggest that there is insufficient evidence to support a robust reverse causal effect of SHUD on the identified cortical morphology.

### 3.5 Sensitivity Analysis

To evaluate the robustness of our bidirectional MR findings, a series of sensitivity analyses were performed. The leave‐one‐out sensitivity analysis demonstrated that the pooled effect estimates remained consistent and were not disproportionately influenced by any individual SNP, as all results consistently remained on the same side of the null (*β* = 0) (Data S3). The MR‐Egger intercept test showed no evidence of horizontal pleiotropy, with intercepts closely centred around zero and all *p* > 0.05 (Tables S3 and S4). Furthermore, no significant heterogeneity was detected, as indicated by Cochran's *Q* and Rücker's *Q* statistics for both IVW and MR‐Egger methods (all *p* > 0.05; Data S4). Collectively, these results reinforce the stability and reliability of our MR‐based causal inferences.

## 4 Discussion

Although brain IDPs have been widely implicated in various substance use disorders, their causal links with SHUD have remained elusive. Using a bidirectional two‐sample MR framework, we systematically evaluated 3935 IDPs, identifying 34 specific brain structural and functional features that are causally associated with SHUD risk. In contrast to the robustness of the forward MR findings, the reverse MR analysis yielded minimal evidence of SHUD‐induced brain alterations. This marked asymmetry suggests that the relationship between brain morphology and SHUD is predominantly unidirectional, with pre‐existing brain structural and functional architectures primarily serving as predisposing causal drivers of disease susceptibility rather than being downstream consequences of the disorder.

A core finding of our study is the causal association between SHUD risk and structural alterations in the temporal and limbic regions. Sedative‐hypnotics, primarily BZDs and Z‐drugs, exert their effects via ${\text{GABA}}_{A}$ receptors, which are densely distributed throughout the cerebral cortex and hippocampus [1]. Our results showed that increased cortical thickness in the transverse and middle temporal gyri is associated with higher SHUD risk. Although cortical thinning is commonly observed in advanced stage of neurodegenerative diseases, our finding of an association between increased cortical thickness and elevated SHUD risk points towards a distinct neurobiological vulnerability. This phenomenon may be explained by aberrant neurodevelopmental trajectories, particularly a deficit in synaptic pruning [18]. During late adolescence and early adulthood, which signifies the peak age of onset in our cohort, the brain undergoes extensive pruning of redundant synapses. A failure or delay in this process can result in a thicker but computationally inefficient cortex, which has been linked to increased impulsivity and susceptibility to addictive behaviours [19]. This is consistent with recent biophysical modelling suggesting that normative cortical maturation is indexed by a declining excitation–inhibition ratio; a failure in this maturation process, potentially manifesting as an abnormally thicker cortex, may serve as a marker of neurocognitive vulnerability [20]. Furthermore, as sedative‐hypnotic target ${\text{GABA}}_{A}$ receptors, a thicker temporal cortex might reflect a higher baseline density of GABAergic interneurons or receptor complexes [21]. This increased biological substrate could potentially amplify the pharmacological effects of sedative drugs, reinforcing drug‐seeking behaviour and elevating the risk of progression to a use disorder [22]. Interestingly, chronic exposure to BZDs has been shown to trigger nanoscale reorganization of ${\text{GABA}}_{A}$ receptors and scaffold destabilization [23], suggesting that individuals with a higher baseline receptor density may experience more profound plastic changes in response to drug use.

Specifically, the positive association between the white‐to‐grey matter (WG) contrast in the parahippocampal gyrus and insula with SHUD risk is noteworthy. The WG contrast is a sensitive marker of cortical myelination and structural integrity [24]. The insula serves as a critical hub of the SN, functioning to segregate the most relevant among internal and extrapersonal stimuli to guide behaviour [25]. Elevated WG contrast and atypical myelination in this region may disrupt the salience mapping process. Such neurostructural profiles may bias individuals towards overvaluing drug‐related cues while becoming less sensitive to internal homeostatic exhaustion, which represents a core mechanism of addiction [26]. Recent neuroimaging research underscores that the SN, primarily comprising the anterior insula and anterior cingulate cortex, plays a key role in detecting salient stimuli; its structural and functional alterations are repeatedly linked to abnormal salience attribution and impaired cognitive control in individuals with substance use disorders [27].

Conversely, we identified protective factors in subcortical structures and white matter tracts. We found that larger brainstem volume was associated with a reduced risk of SHUD (OR = 0.74). Given the role of the brainstem in regulating the ascending reticular activating system (ARAS) and its high density of GABAergic neurons, a robust brainstem volume might serve as a biological buffer against the sedative‐induced desensitization of arousal systems [28]. Furthermore, the protective role of the cingulum bundle (OR = 0.73) and the FPN (edge 263, OR = 0.61) reinforces the importance of top‐down inhibitory control. Our findings suggest that superior structural connectivity in these pathways may enhance inhibitory control and emotional resilience, thereby mitigating the progression from initial sedative use to chronic use disorder [29, 30]. This aligns with evidence that resilience against addiction is characterized by hyperconnectivity in circuits implicated in the regulation of habits and top‐down inhibitory control [31].

At the functional level, our MR analysis highlights that SHUD susceptibility is characterized by the dysregulation of functional brain architecture. The identified causal relationships involving the SN, DMN and VN support the triple network model of addiction [32, 33]. Higher amplitude in the SN (ICA25 node 11) was protective (OR = 0.70), suggesting that stable intrinsic SN activity allows for efficient switching from the DMN's internal cravings to the FPN's executive control. This is consistent with recent evidence identifying the insula as a causal hub that mediates uncoordinated switching between network activities in substance use disorders [25]. Notably, the VN emerged as a central connectivity hub. Although often overlooked in addiction research, the consistent protective effects between the VN and SMN (e.g., edges 834 and 1118) suggest that efficient sensorimotor integration acts as a resilience factor. This coordination may facilitate higher‐order sensory filtering, reducing the likelihood that visual drug cues are translated into impulsive motor actions [34, 35].

Reverse MR analysis revealed a nominal association between SHUD liability and increased cortical thickness in the rMTG (OR = 1.03). Although the rMTG is integral to cognitive processing [36], this negligible effect warrants considerable caution. The association failed to show consistency in sensitivity analyses, indicating limited robustness and a potential risk of false‐positive discovery. Consequently, these limited findings provide only tenuous evidence for reverse causality and do not support a bidirectional feedback mechanism. Instead, the absence of robust reverse signals substantiates our forward MR findings, reinforcing that the identified IDPs represent predisposing neurobiological vulnerabilities rather than secondary consequences of chronic substance exposure. Evidence suggests that although chronic substance use can alter brain structure, many morphological deviations, particularly those concerning cortical thickness, may predate the onset of substance use disorders [37].

Several limitations should be acknowledged. First, the IVs' strength for SHUD in the reverse MR was lower than that for the brain IDPs, which may have limited our power to detect subtle reverse causal effects. Second, our data primarily derive from European ancestry populations, necessitating further validation of cross‐population generalizability. Third, MR cannot fully exclude gene–environment interactions, whereas sedative‐hypnotic use is substantially influenced by environmental and psychosocial factors. Fourth, this study focused on static imaging phenotypes; integrating longitudinal data, transcriptomic information and multimodal imaging would provide more comprehensive insights into the neurobiological mechanisms underlying sedative‐hypnotic use disorders.

## 5 Conclusion

In summary, this study provides genetic evidence suggesting a predominantly unidirectional causal influence of brain architecture on SHUD risk. By identifying 34 potential causal IDPs involving temporal–limbic and functional networks, our findings indicate that pre‐existing neurobiological substrates may primarily drive disease susceptibility, rather than being mere consequences of drug use. Although these MR‐based inferences are subject to inherent limitations, they support an innate vulnerability model for SHUD. Overall, this work offers a preliminary causal framework and identifies candidate neuroimaging biomarkers that warrant further longitudinal validation for risk stratification and preventive strategies.

## Author Contributions

Fenglan Li and Xiaoli Zhu designed and conceptualized the study. Liqin Lu and Guoxin Zhuang drafted the manuscript and performed critical revisions. Jue Deng and Siheng Huang performed data collection and statistical analysis. Liang Meng supervised the study, had full access to all data and takes responsibility for the integrity of the data and the decision to submit for publication. All authors reviewed and approved the final manuscript.

## Funding

This work was supported by grants from the Natural Science Foundation of Fujian Province (2024J08116, 2023J05082 and 2024J08115) and the Fujian Provincial Department of Finance Special Research Project for Provincial‐level Units (Mincaizhi[2020]822).

## Ethics Statement

This study utilized publicly available GWAS summary statistics. No additional ethical approval or informed consent was required as all original studies had obtained appropriate ethical approvals and participant consent.

## Consent

The authors have nothing to report.

## Conflicts of Interest

The authors declare no conflicts of interest.

## Supporting information


**Table S1:** Age distribution at first onset of mental and behavioural disorders.

**Table S2:** The information of IVs for mental and behavioural disorders due to sedative‐hypnotic drug abuse in reverse MR.

**Table S3:** MR‐Egger intercept test for horizontal pleiotropy in forward MR.

**Table S4:** MR‐Egger intercept test for horizontal pleiotropy in reverse MR.

**Data S1:** Supporting Information.

**Data S2:** Supporting Information.

**Data S3:** Supporting Information.

**Data S4:** Supporting Information.

## Data Availability

GWAS summary statistics of brain IDPs were obtained from the Oxford Brain Imaging Genetics (BIG40) web server (https://open.win.ox.ac.uk/ukbiobank/big40/). GWAS summary statistics of sedative‐hypnotic use disorders were obtained from the FinnGen study (https://r10.risteys.finngen.fi/endpoints/F5_SEDAHYP).
