# Supplementary material for: Investigating Bidirectional Causal Relationships Between Imaging‐Derived Brain Phenotypes and Sedative‐Hypnotic Use Disorder: A Mendelian Randomization Study
Source: Addict Biol. 2026 May 28;31(6):e70160. doi: 10.1111/adb.70160 (PMC13239158; doi:10.1111/adb.70160)
Supplement: Supplementary file 1 — Table S1: Age distribution at first onset of mental and behavioural disorders. [file ADB-31-e70160-s007.docx]

**Table S1** Age distribution at first onset of mental and behavioral disorders.

| Age group (years) | N (%) of participants | Mean age to onset(years) | Male mean age at onset(years) | Female mean age at onset(years) |
| --- | --- | --- | --- | --- |
| 10-20 | 119(5.3%) |  |  |  |
| 20-30 | 594(26.3%) |  |  |  |
| 30-40 | 514(22.7%) |  |  |  |
| 40-50 | 438(19.4%) |  |  |  |
| 50-60 | 298(13.2%) |  |  |  |
| 60-70 | 168(7.4%) |  |  |  |
| 70-80 | 101(4.5%) |  |  |  |
| 80 above | 26(1.2%) |  |  |  |
| Overall | 2258(100%) | 40.56 | 41.24 | 39.39 |
