# Supplementary material for: Investigating Bidirectional Causal Relationships Between Imaging‐Derived Brain Phenotypes and Sedative‐Hypnotic Use Disorder: A Mendelian Randomization Study
Source: Addict Biol. 2026 May 28;31(6):e70160. doi: 10.1111/adb.70160 (PMC13239158; doi:10.1111/adb.70160)
Supplement: Supplementary file 3 — Table S3: MR‐Egger intercept test for horizontal pleiotropy in forward MR. [file ADB-31-e70160-s002.docx]

**Table S3 MR-Egger intercept test for horizontal pleiotropy in forward MR.**

| id.exposure | id.outcome | outcome | exposure | egger_intercept | se | p-val |
| --- | --- | --- | --- | --- | --- | --- |
| zp14oS | XrkhT3 | Mental and behavioural disorders due to sedatives or hypnotics | GCST90002542 | 0.000 | 0.01 | 0.97 |
| 7hsUC5 | XrkhT3 |  | GCST90002561 | -0.004 | 0.02 | 0.84 |
| 2zm0b9 | XrkhT3 |  | GCST90002780 | -0.009 | 0.01 | 0.53 |
| 5JEIcS | XrkhT3 |  | GCST90002873 | -0.004 | 0.01 | 0.77 |
| ybqd2M | XrkhT3 |  | GCST90002946 | 0.005 | 0.02 | 0.74 |
| 0fu2rd | XrkhT3 |  | GCST90003058 | -0.004 | 0.02 | 0.80 |
| Udthtf | XrkhT3 |  | GCST90003131 | -0.001 | 0.02 | 0.95 |
| BWWfRw | XrkhT3 |  | GCST90003676 | -0.001 | 0.02 | 0.95 |
| DyIStp | XrkhT3 |  | GCST90003714 | -0.005 | 0.01 | 0.73 |
| UPacRJ | XrkhT3 |  | GCST90003733 | 0.000 | 0.02 | 0.96 |
| lxZmhp | XrkhT3 |  | GCST90003807 | 0.000 | 0.02 | 0.99 |
| 3MgmUz | XrkhT3 |  | GCST90003861 | 0.0165 | 0.01 | 0.25 |
| 2D8sge | XrkhT3 |  | GCST90004004 | -0.004 | 0.02 | 0.82 |
| ZeXRe6 | XrkhT3 |  | GCST90004301 | -0.025 | 0.02 | 0.26 |
| hkow3B | XrkhT3 |  | GCST90004578 | -0.004 | 0.02 | 0.83 |
| qQ2NoI | XrkhT3 |  | GCST90004862 | -0.011 | 0.02 | 0.62 |
| 0ODJ0i | XrkhT3 |  | GCST90004898 | 0.003 | 0.01 | 0.80 |
| bAfigz | XrkhT3 |  | GCST90005108 | -0.009 | 0.02 | 0.57 |
| DSx5uH | XrkhT3 |  | GCST90005116 | 0.022 | 0.03 | 0.47 |
| FMyqZp | XrkhT3 |  | GCST90005182 | 0.025 | 0.02 | 0.29 |
| d5214m | XrkhT3 |  | GCST90005248 | -0.003 | 0.02 | 0.91 |
| rJDbCG | XrkhT3 |  | GCST90005372 | 0.000 | 0.02 | 0.99 |
| xaUqwA | XrkhT3 |  | GCST90005384 | 0.024 | 0.03 | 0.48 |
| LUDMHS | XrkhT3 |  | GCST90005629 | -0.006 | 0.02 | 0.72 |
| Iwl9w6 | XrkhT3 |  | GCST90005660 | 0.007 | 0.02 | 0.75 |
| hsbfAE | XrkhT3 |  | GCST90005687 | 0.030 | 0.04 | 0.40 |
| EnxBQG | XrkhT3 |  | GCST90005715 | -0.004 | 0.02 | 0.85 |
| ePygEi | XrkhT3 |  | GCST90005844 | 0.000 | 0.01 | 0.98 |
| KhrXUK | XrkhT3 |  | GCST90005971 | -0.005 | 0.02 | 0.78 |
| qP5Ofh | XrkhT3 |  | GCST90005975 | -0.006 | 0.02 | 0.73 |
| kAd1xJ | XrkhT3 |  | GCST90006239 | 0.000 | 0.02 | 0.99 |
| VQvlbB | XrkhT3 |  | GCST90006306 | 0.028 | 0.02 | 0.10 |
| UZwR21 | XrkhT3 |  | GCST90006308 | -0.000 | 0.02 | 0.99 |
| Ff7kQk | XrkhT3 |  | GCST90006319 | 0.017 | 0.03 | 0.50 |
