# Supplementary material for: Investigating Bidirectional Causal Relationships Between Imaging‐Derived Brain Phenotypes and Sedative‐Hypnotic Use Disorder: A Mendelian Randomization Study
Source: Addict Biol. 2026 May 28;31(6):e70160. doi: 10.1111/adb.70160 (PMC13239158; doi:10.1111/adb.70160)
Supplement: Supplementary file 4 — Table S4: MR‐Egger intercept test for horizontal pleiotropy in reverse MR. [file ADB-31-e70160-s006.docx]

**Table S4 MR-Egger intercept test for horizontal pleiotropy in reverse MR.**

| id.exposure | id.outcome | outcome | | exposure | egger_intercept | | se | p-val |
| --- | --- | --- | --- | --- | --- | --- | --- | --- |
| oIq39k | VfyXgM | | GCST90003714 | Mental and behavioural disorders due to sedatives or hypnotics | | -0.008 | 0.00 | 0.18 |
