# Supplementary figures and images for: Investigating Bidirectional Causal Relationships Between Imaging‐Derived Brain Phenotypes and Sedative‐Hypnotic Use Disorder: A Mendelian Randomization Study
Source: Addict Biol. 2026 May 28;31(6):e70160. doi: 10.1111/adb.70160 (PMC13239158; doi:10.1111/adb.70160)

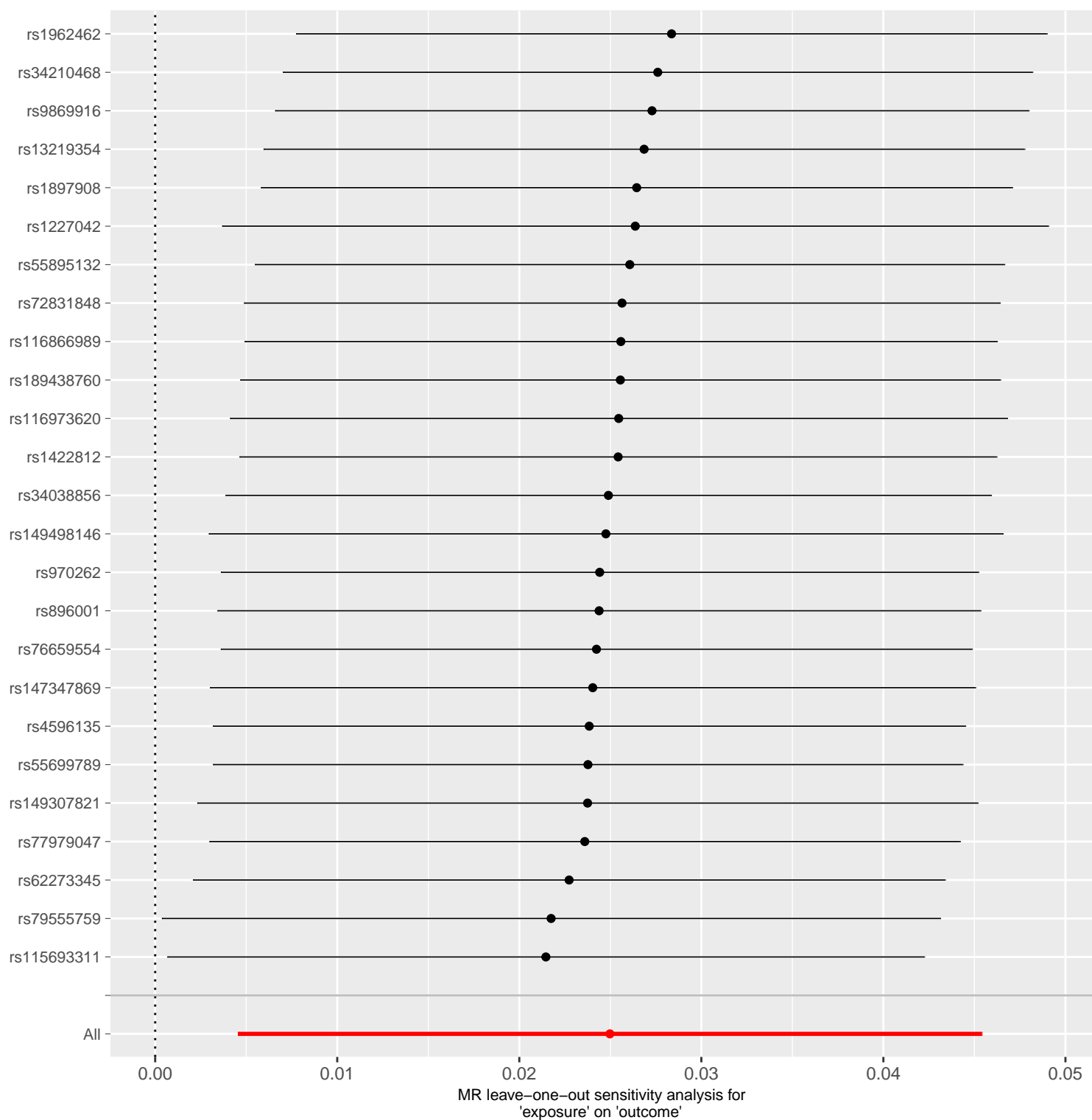

Supplement: Supplementary file 7 — Data S3: Supporting Information. [file ADB-31-e70160-s008.zip › Additional file3/Reverse MR analysis.pdf]

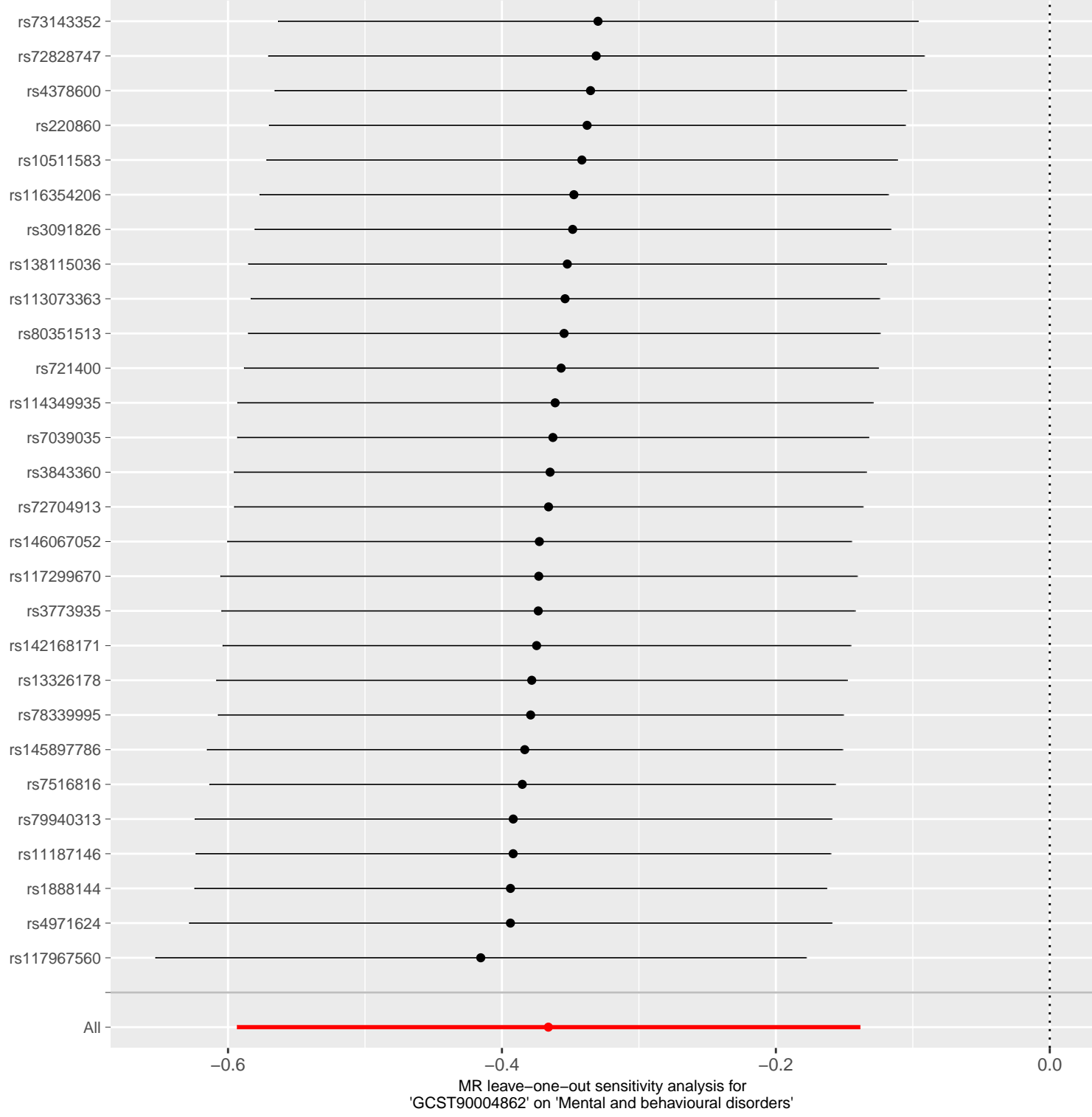

Supplement: Supplementary file 7 — Data S3: Supporting Information. [file ADB-31-e70160-s008.zip › Additional file3/Forward MR analysis/GCST90004862.pdf]

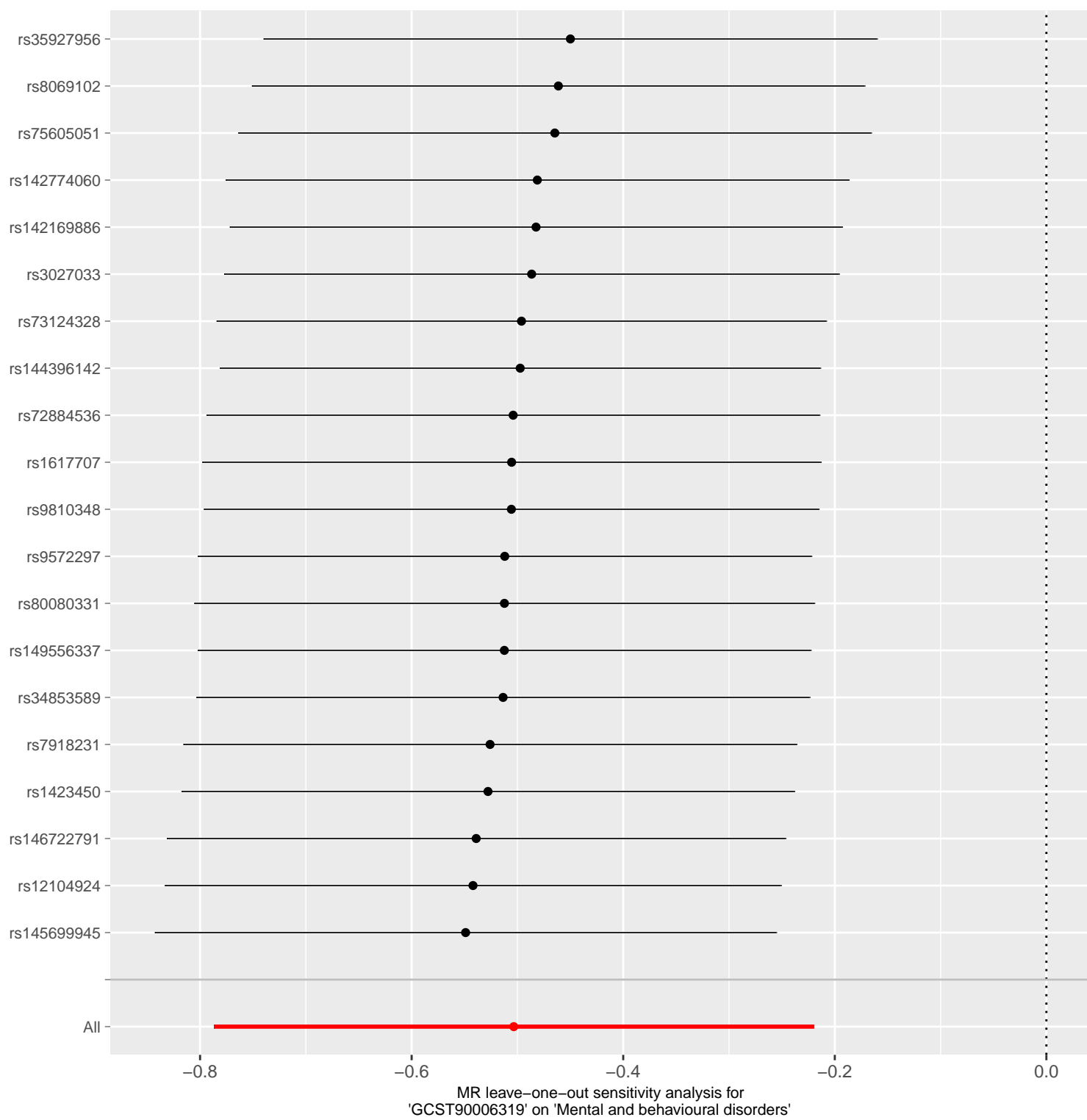

Supplement: Supplementary file 7 — Data S3: Supporting Information. [file ADB-31-e70160-s008.zip › Additional file3/Forward MR analysis/GCST90006319.pdf]

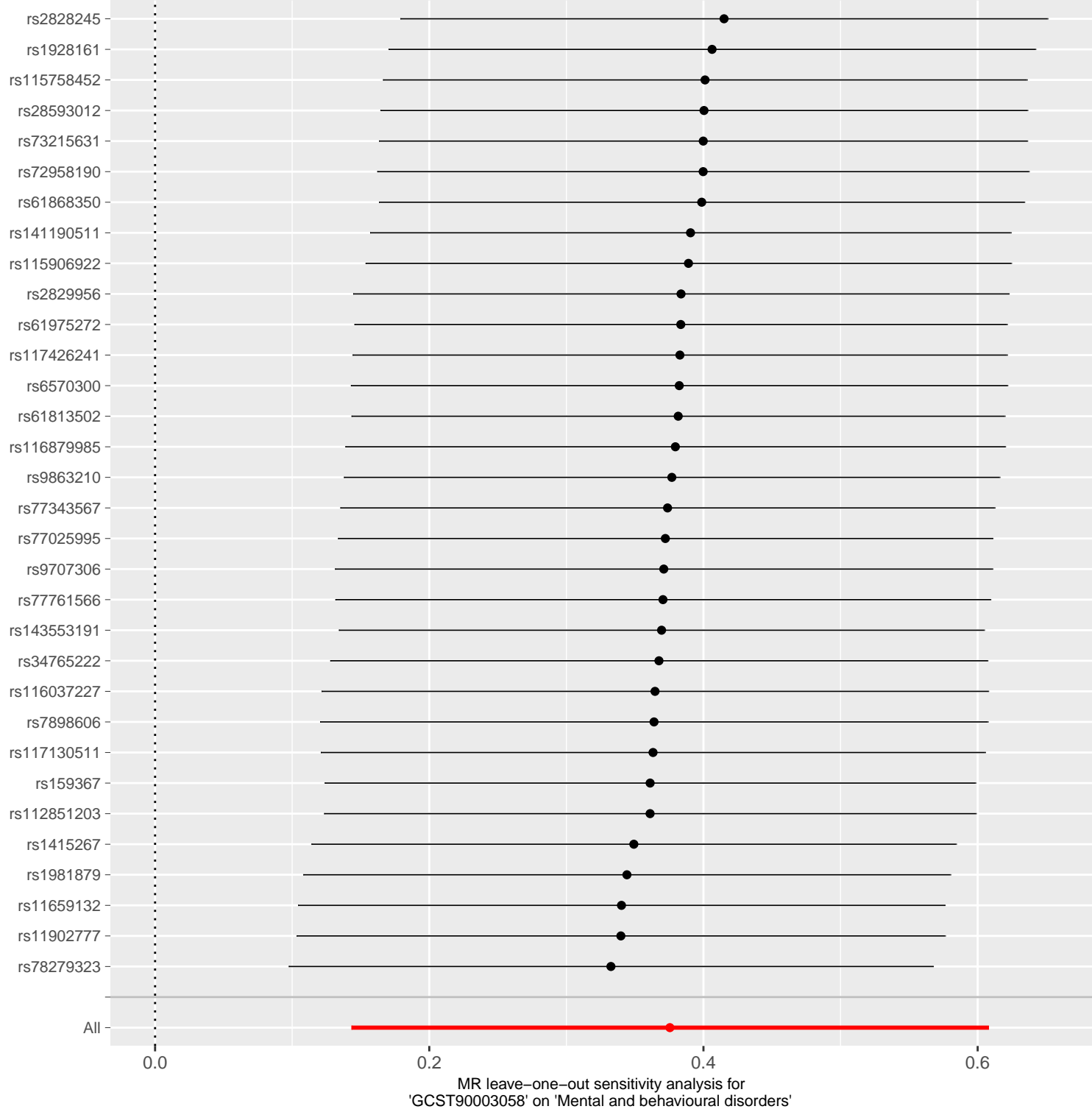

Supplement: Supplementary file 7 — Data S3: Supporting Information. [file ADB-31-e70160-s008.zip › Additional file3/Forward MR analysis/GCST90003058.pdf]

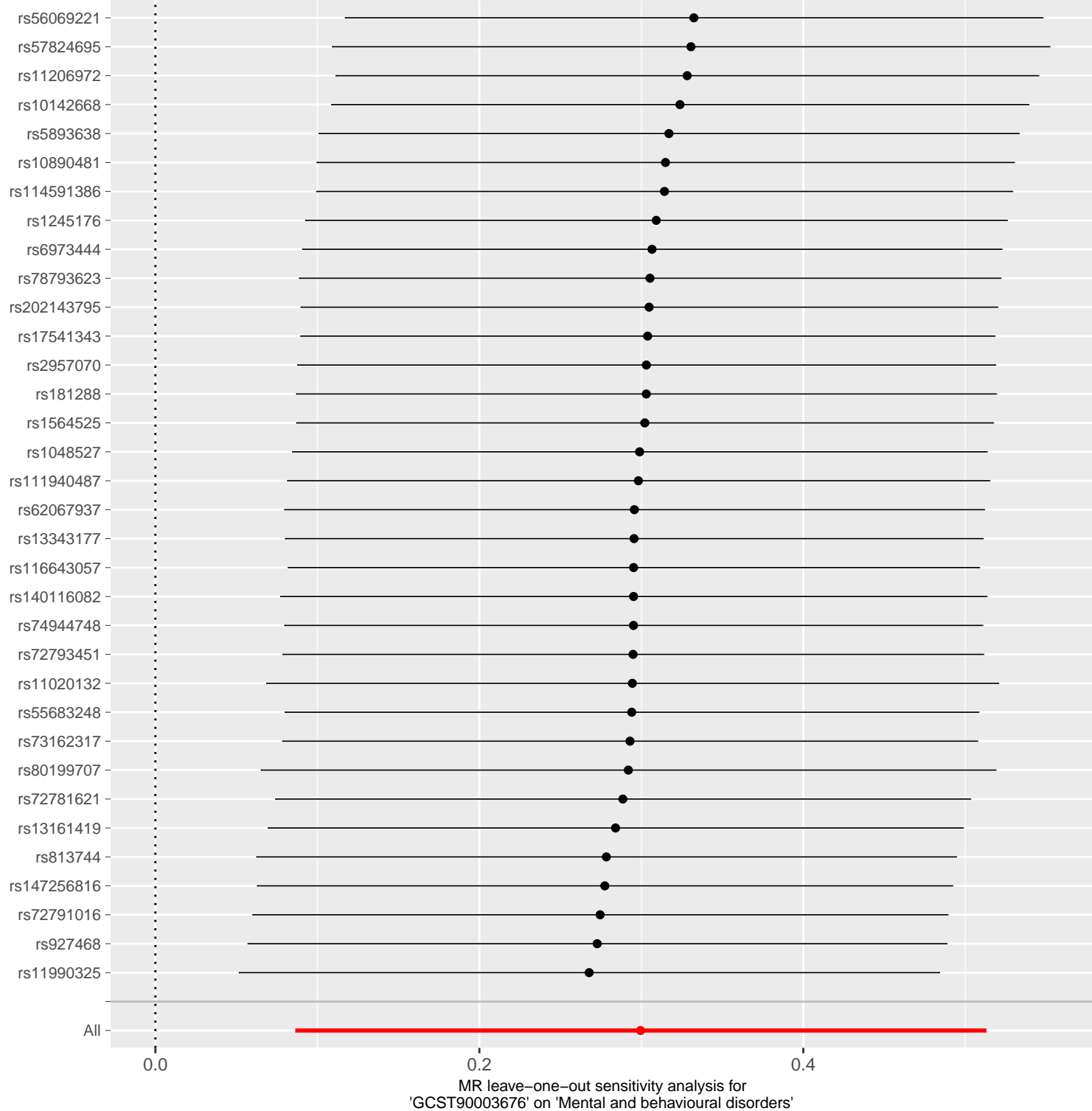

Supplement: Supplementary file 7 — Data S3: Supporting Information. [file ADB-31-e70160-s008.zip › Additional file3/Forward MR analysis/GCST90003676.pdf]

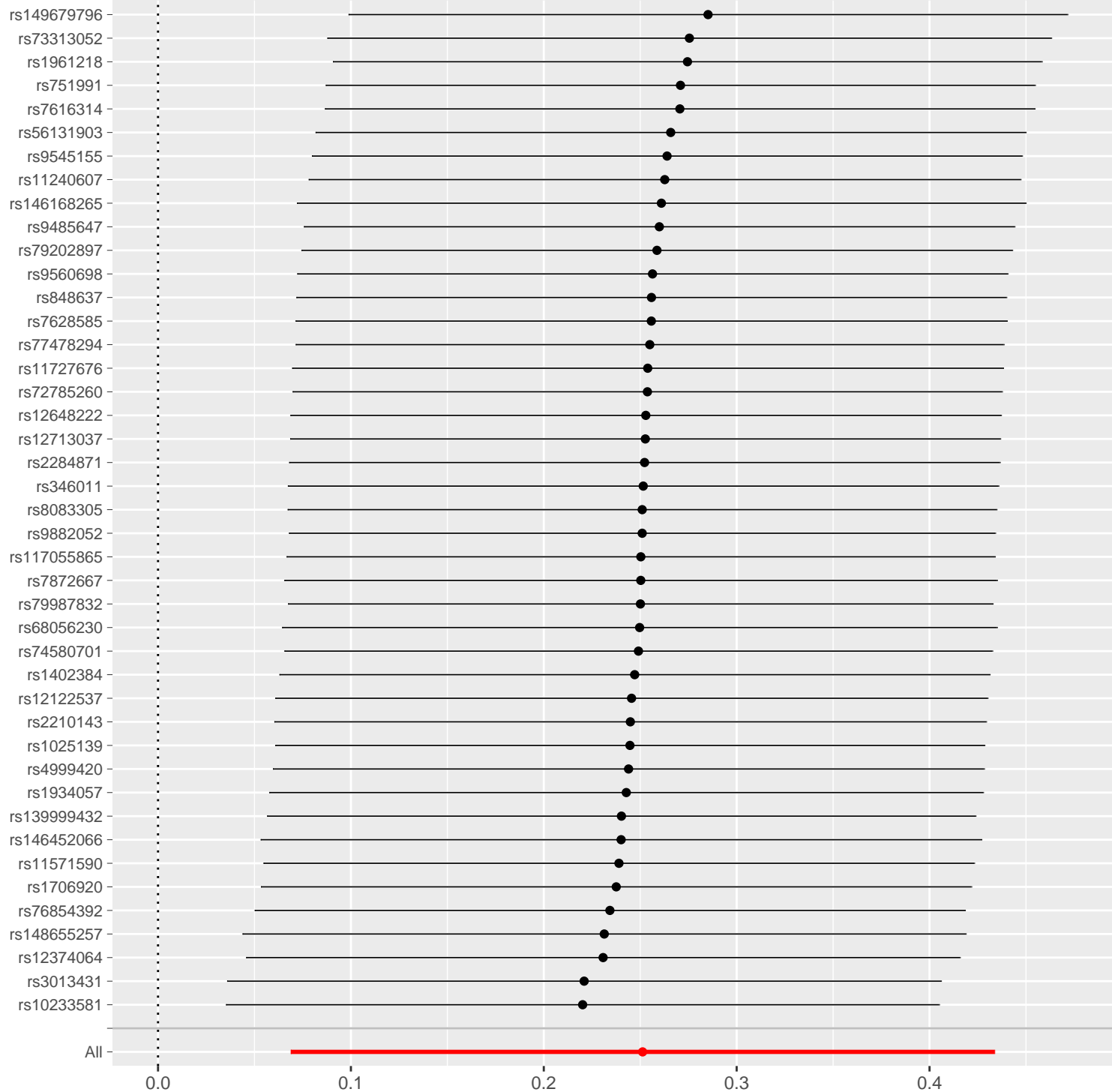

MR leave-one-out sensitivity analysis for  
'GCST90002780' on 'Mental and behavioural disorders'

Supplement: Supplementary file 7 — Data S3: Supporting Information. [file ADB-31-e70160-s008.zip › Additional file3/Forward MR analysis/GCST90002780.pdf]

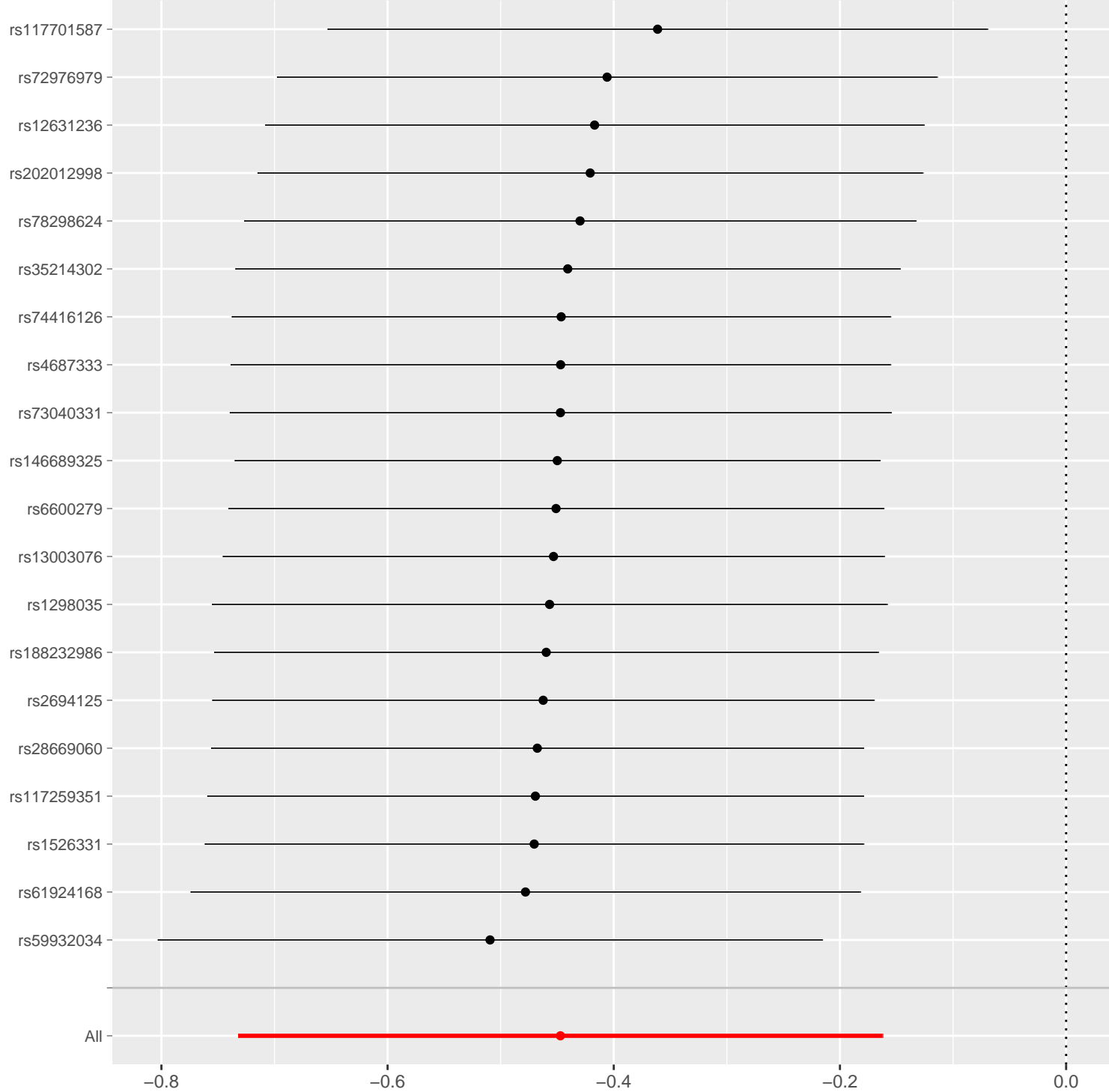

Supplement: Supplementary file 7 — Data S3: Supporting Information. [file ADB-31-e70160-s008.zip › Additional file3/Forward MR analysis/GCST90005372.pdf]

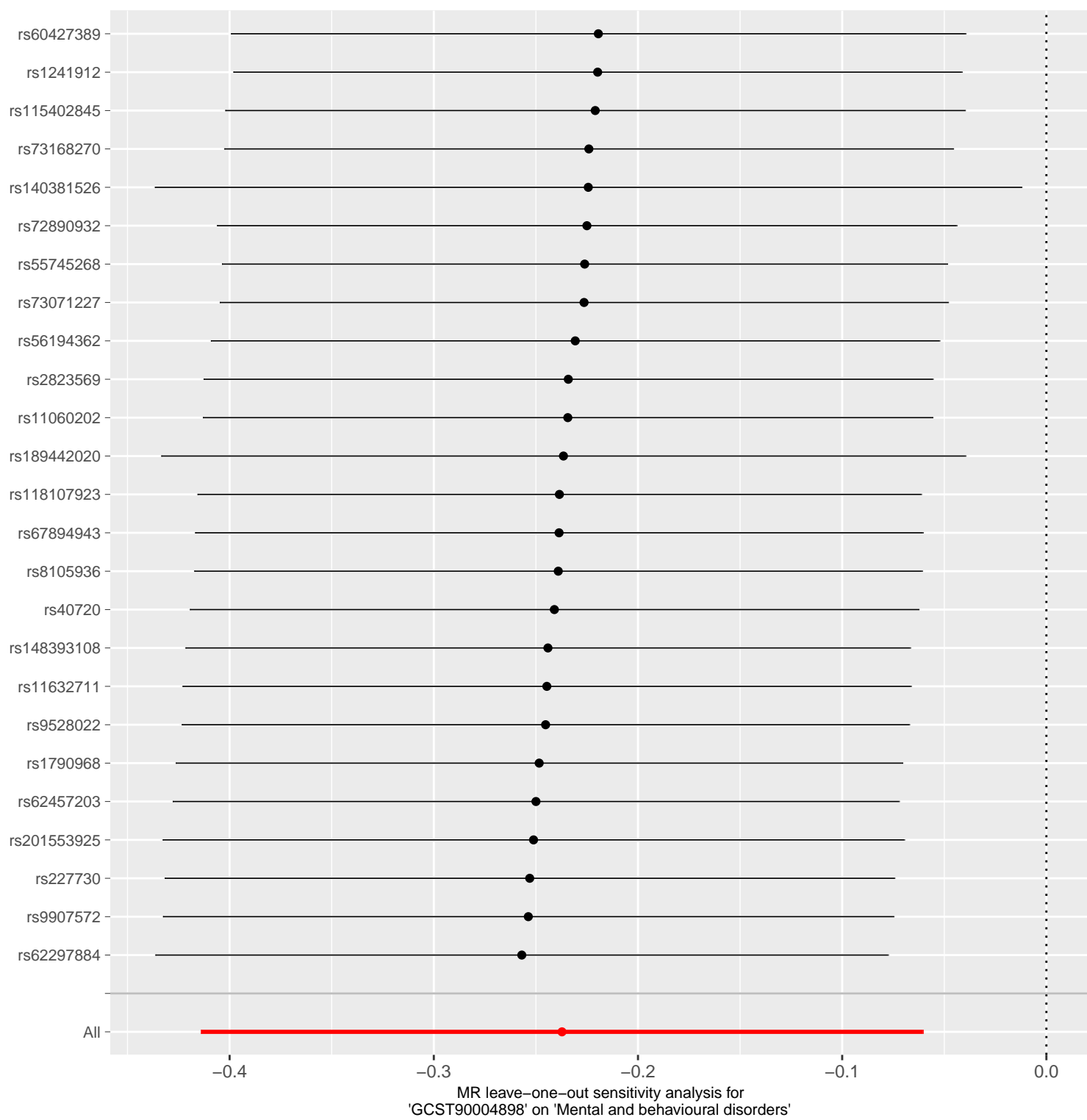

Supplement: Supplementary file 7 — Data S3: Supporting Information. [file ADB-31-e70160-s008.zip › Additional file3/Forward MR analysis/GCST90004898.pdf]

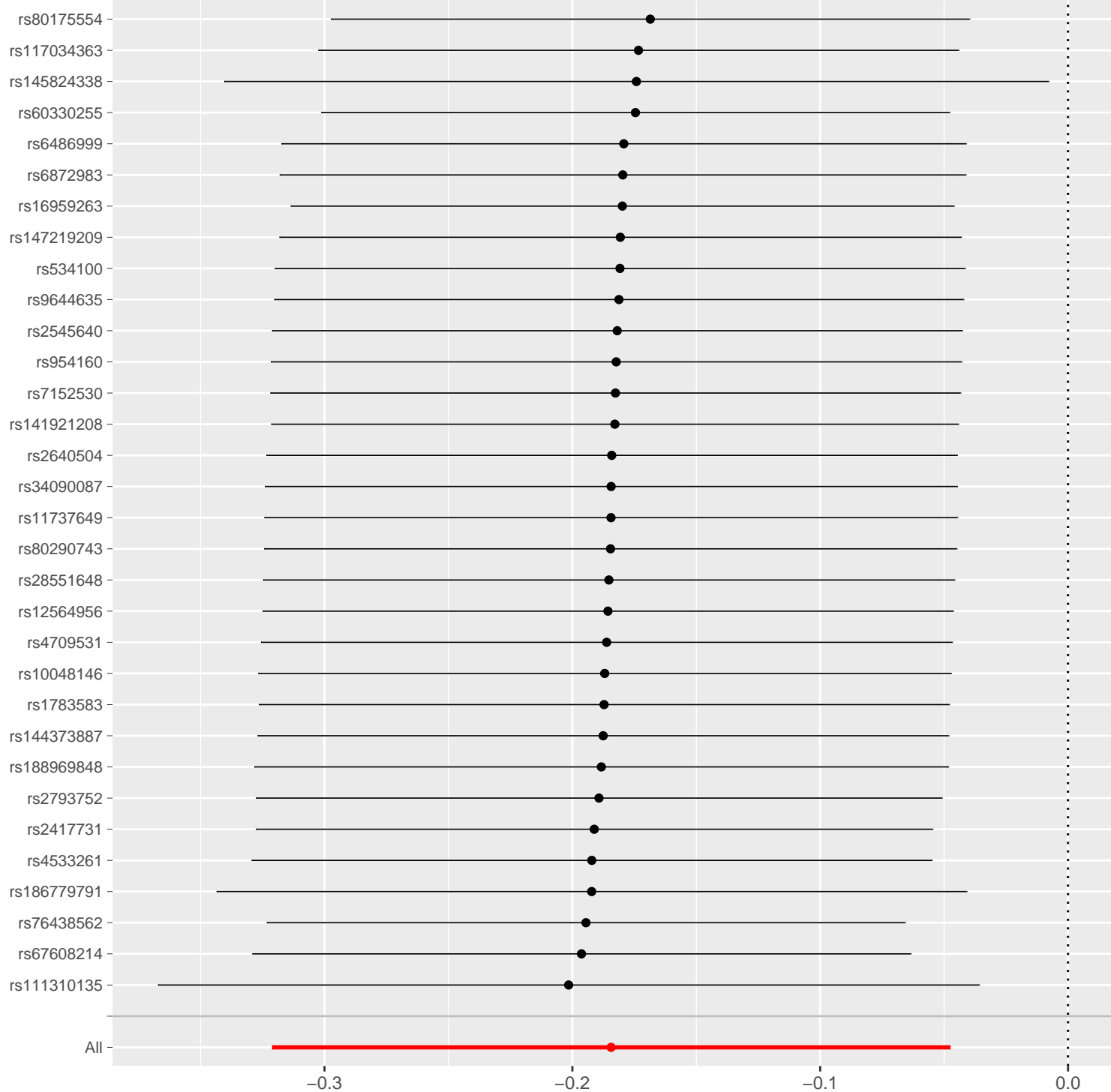

Supplement: Supplementary file 7 — Data S3: Supporting Information. [file ADB-31-e70160-s008.zip › Additional file3/Forward MR analysis/GCST90002542.pdf]

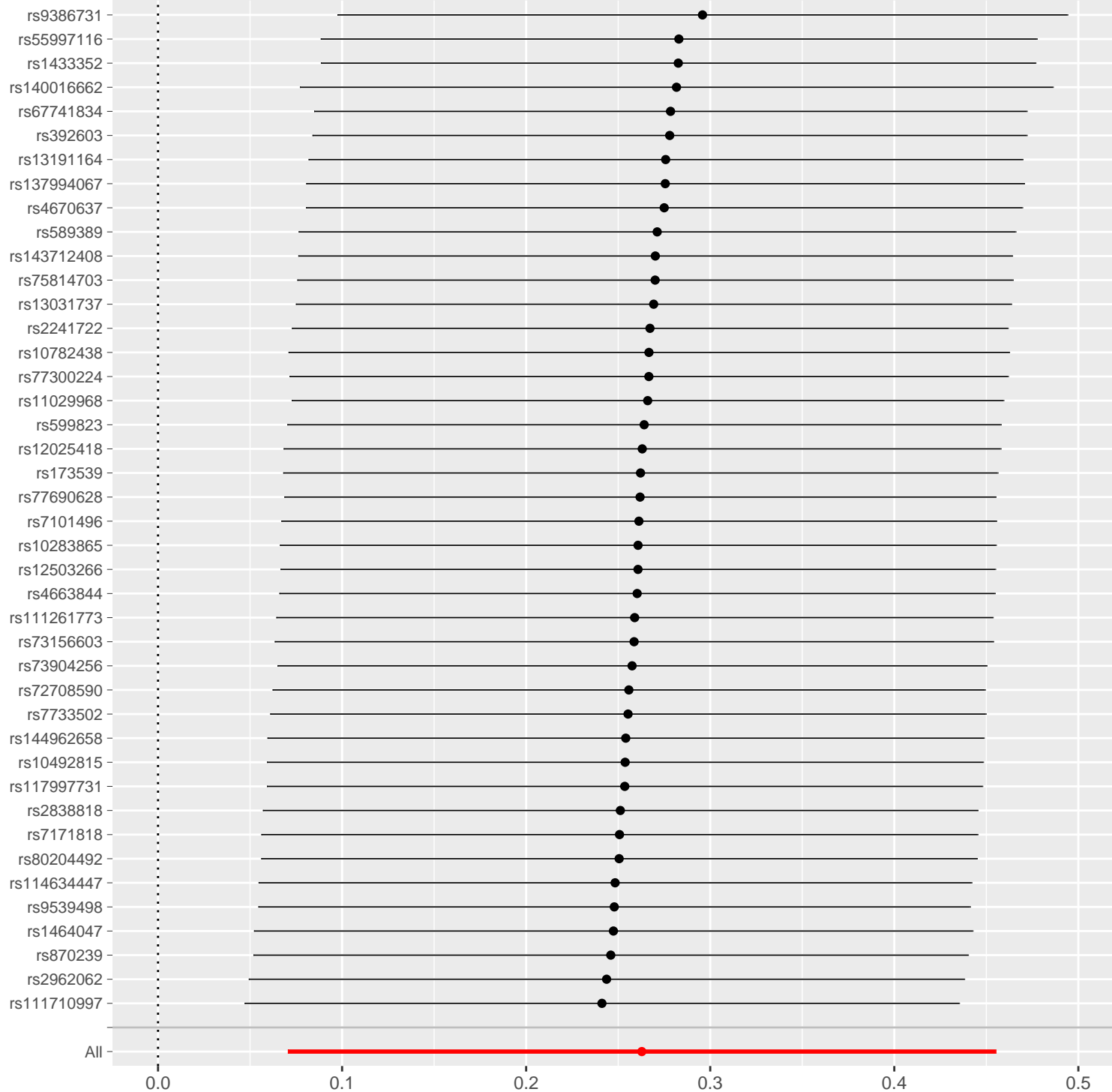

Supplement: Supplementary file 7 — Data S3: Supporting Information. [file ADB-31-e70160-s008.zip › Additional file3/Forward MR analysis/GCST90003714.pdf]

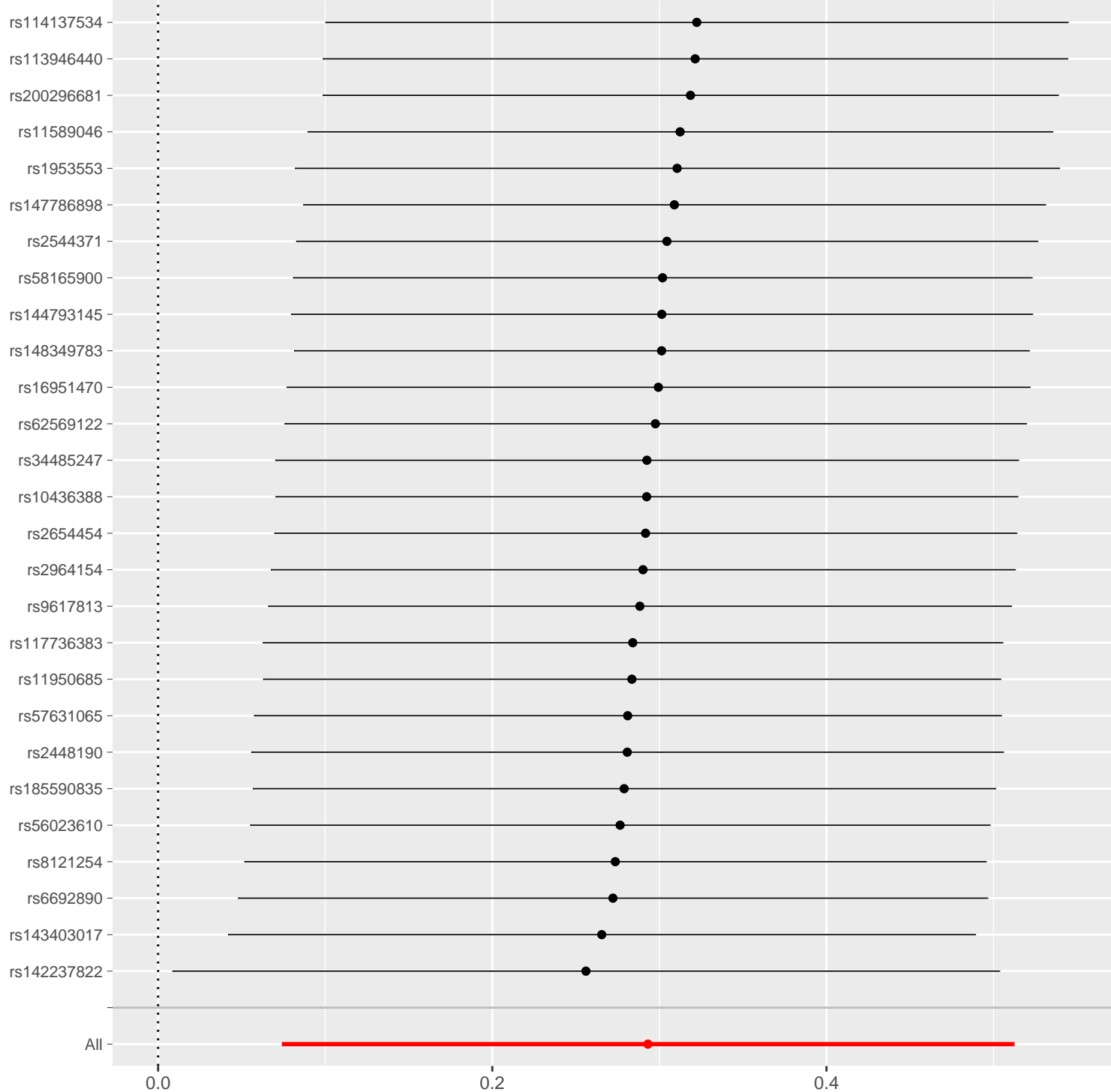

MR leave-one-out sensitivity analysis for  
'GCST90005629' on 'Mental and behavioural disorders'

Supplement: Supplementary file 7 — Data S3: Supporting Information. [file ADB-31-e70160-s008.zip › Additional file3/Forward MR analysis/GCST90005629.pdf]

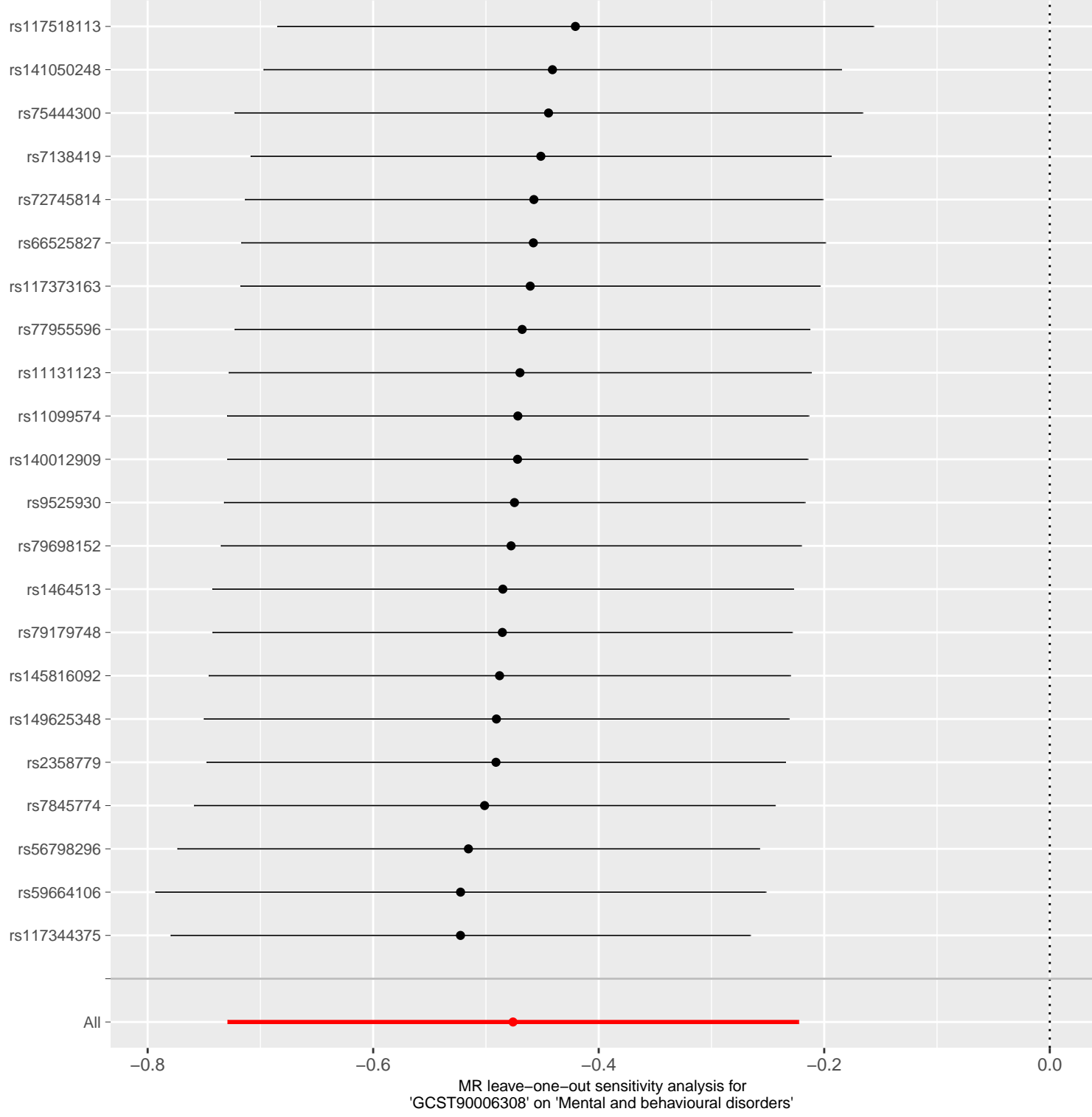

Supplement: Supplementary file 7 — Data S3: Supporting Information. [file ADB-31-e70160-s008.zip › Additional file3/Forward MR analysis/GCST90006308.pdf]

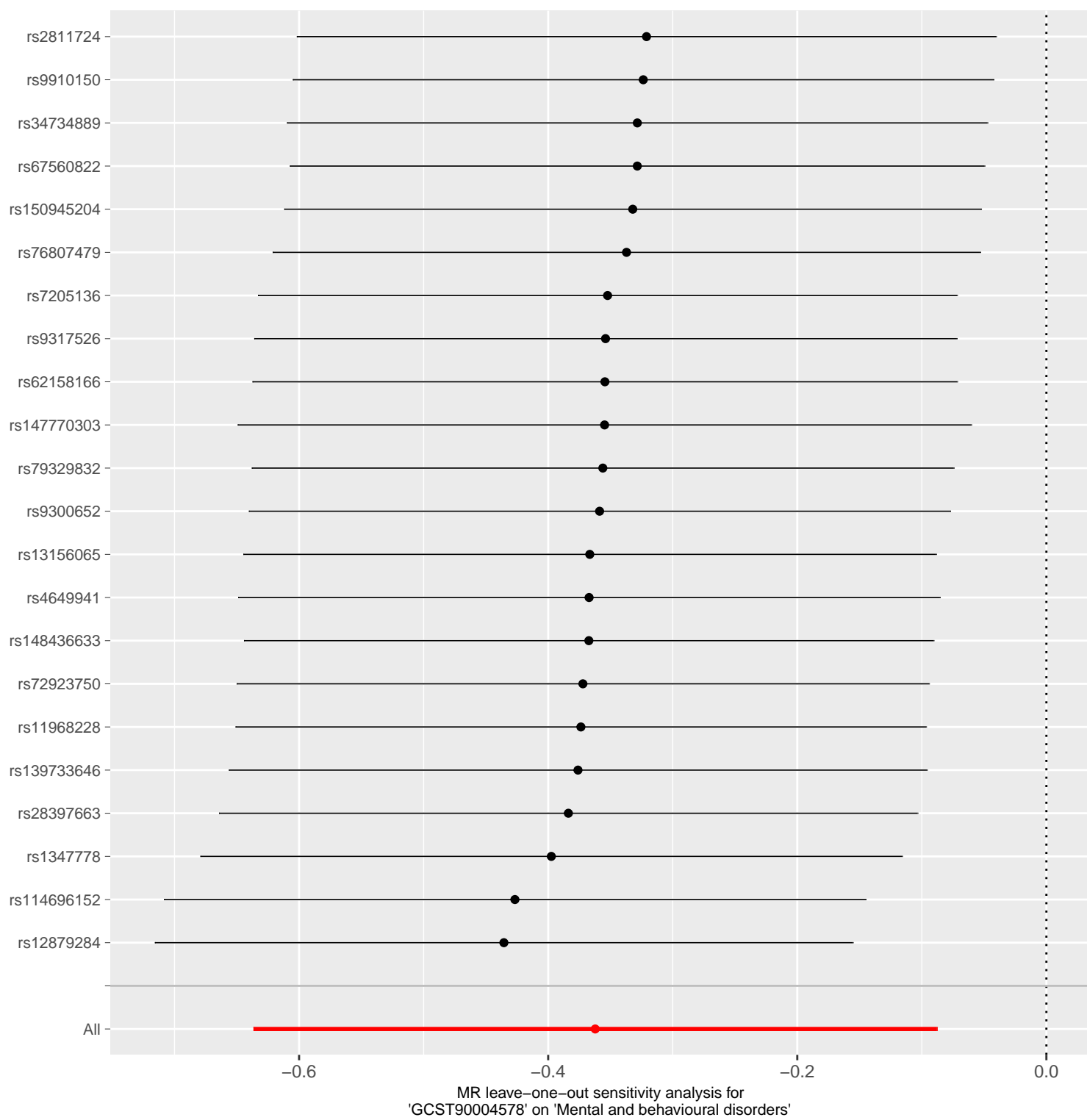

Supplement: Supplementary file 7 — Data S3: Supporting Information. [file ADB-31-e70160-s008.zip › Additional file3/Forward MR analysis/GCST90004578.pdf]

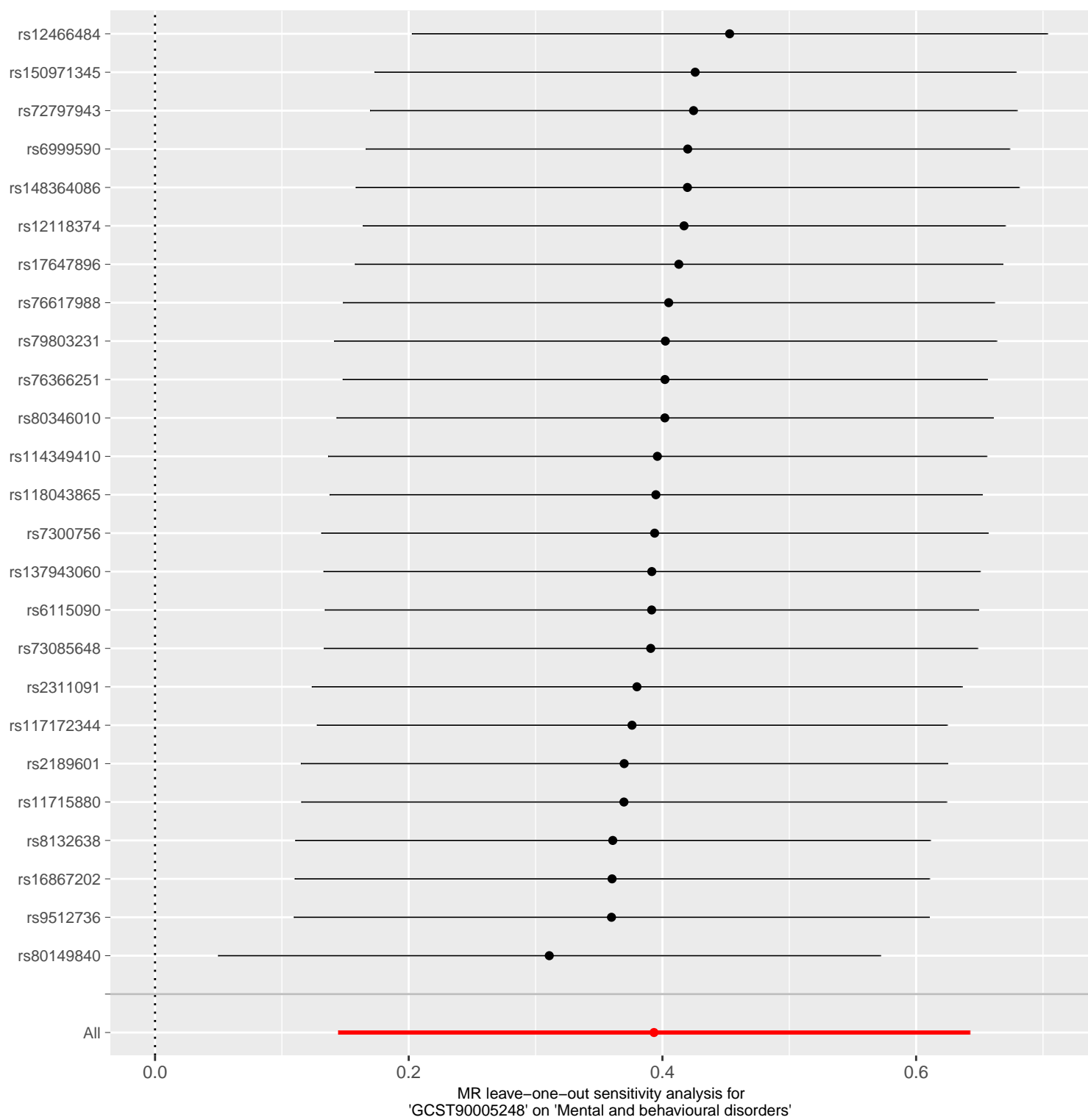

Supplement: Supplementary file 7 — Data S3: Supporting Information. [file ADB-31-e70160-s008.zip › Additional file3/Forward MR analysis/GCST90005248.pdf]

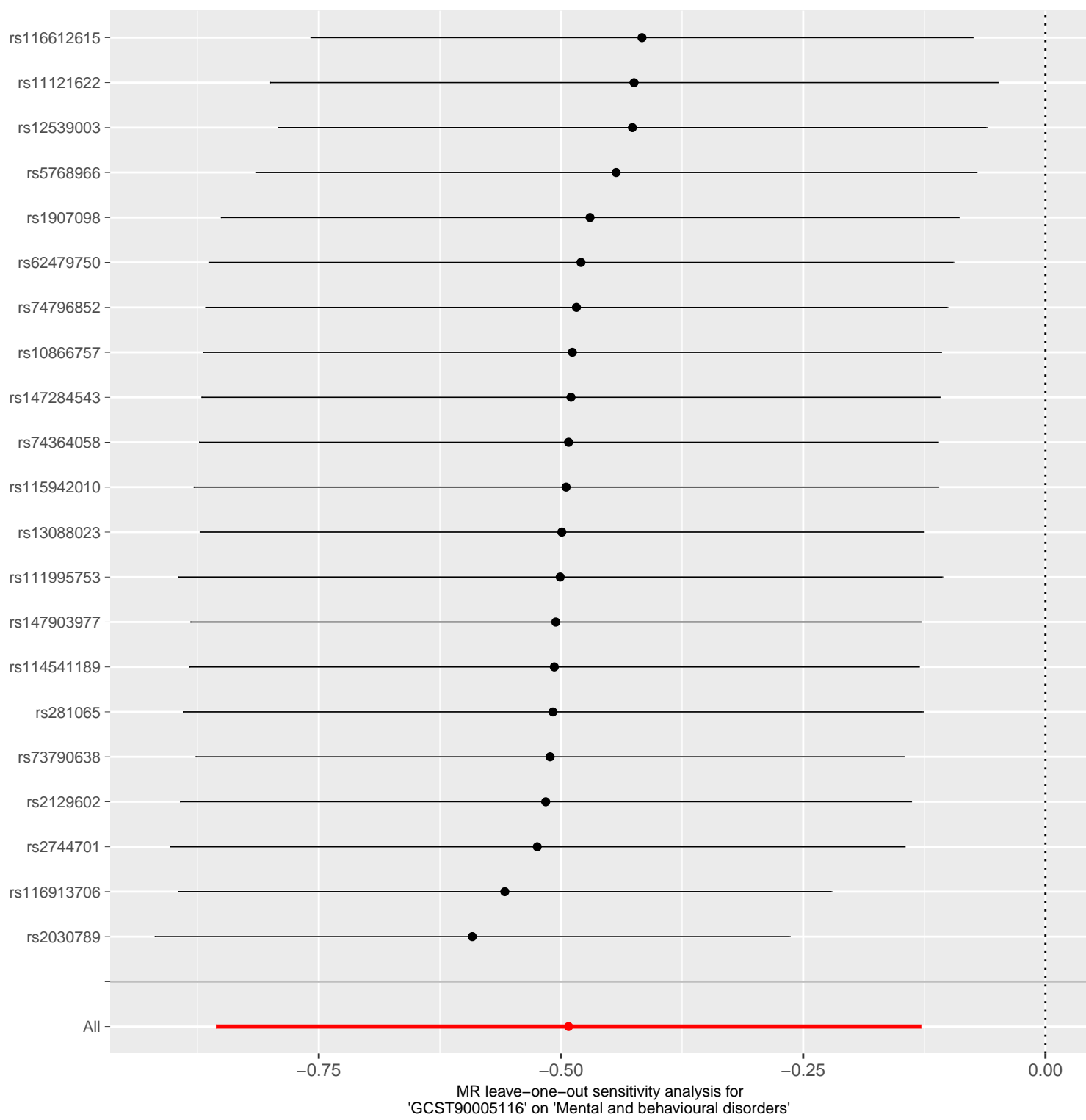

Supplement: Supplementary file 7 — Data S3: Supporting Information. [file ADB-31-e70160-s008.zip › Additional file3/Forward MR analysis/GCST90005116.pdf]

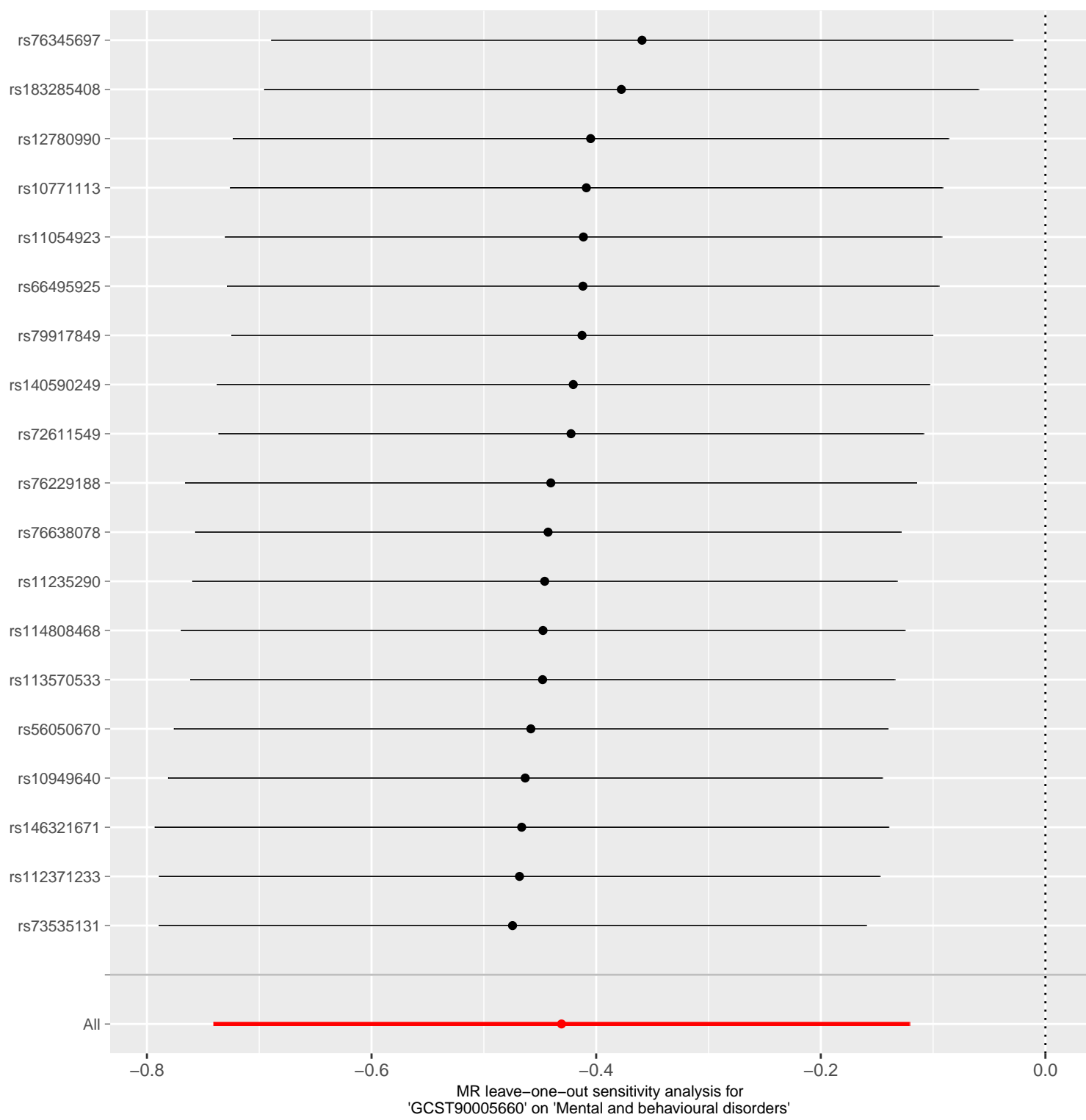

Supplement: Supplementary file 7 — Data S3: Supporting Information. [file ADB-31-e70160-s008.zip › Additional file3/Forward MR analysis/GCST90005660.pdf]

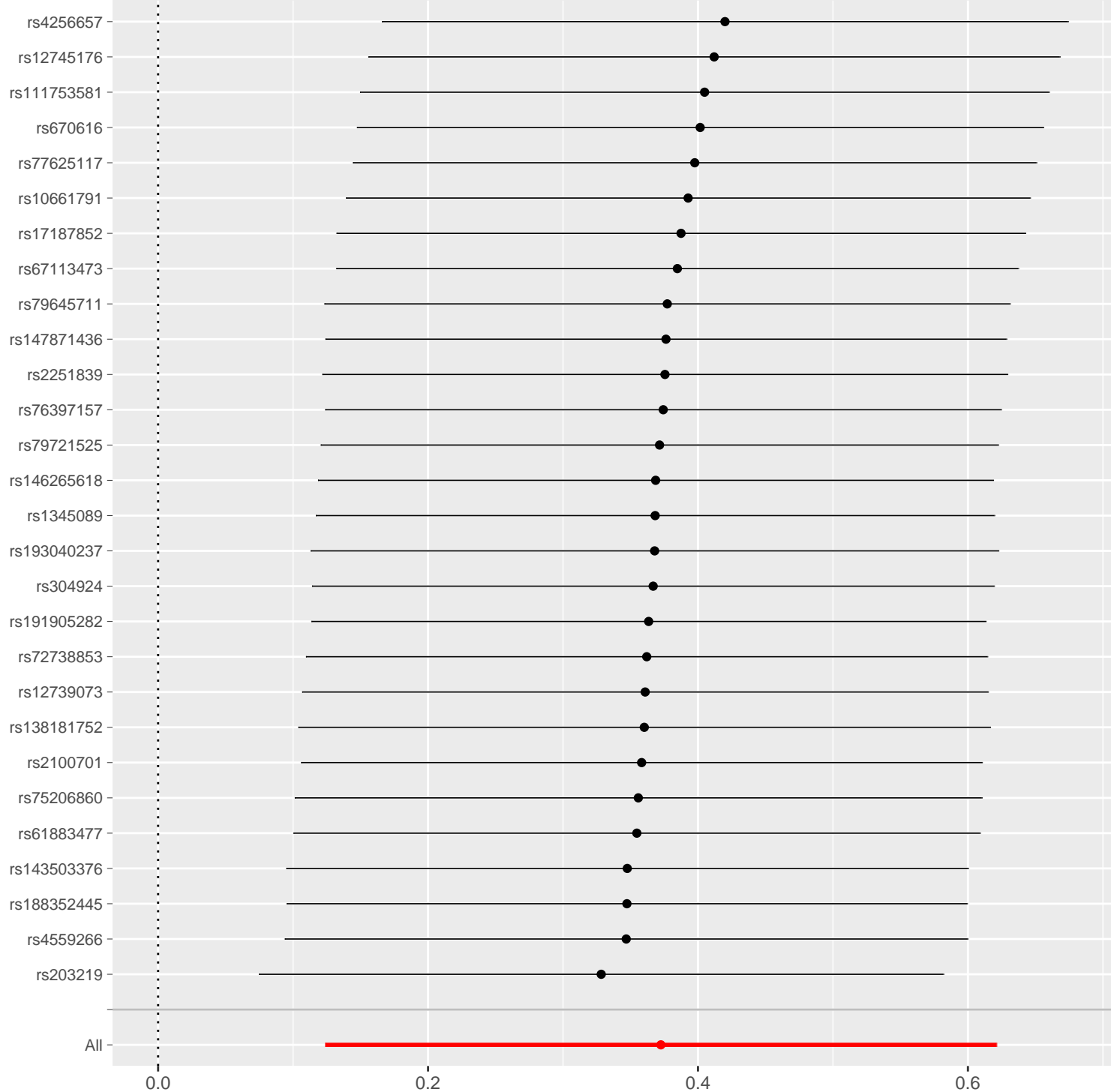

Supplement: Supplementary file 7 — Data S3: Supporting Information. [file ADB-31-e70160-s008.zip › Additional file3/Forward MR analysis/GCST90005715.pdf]

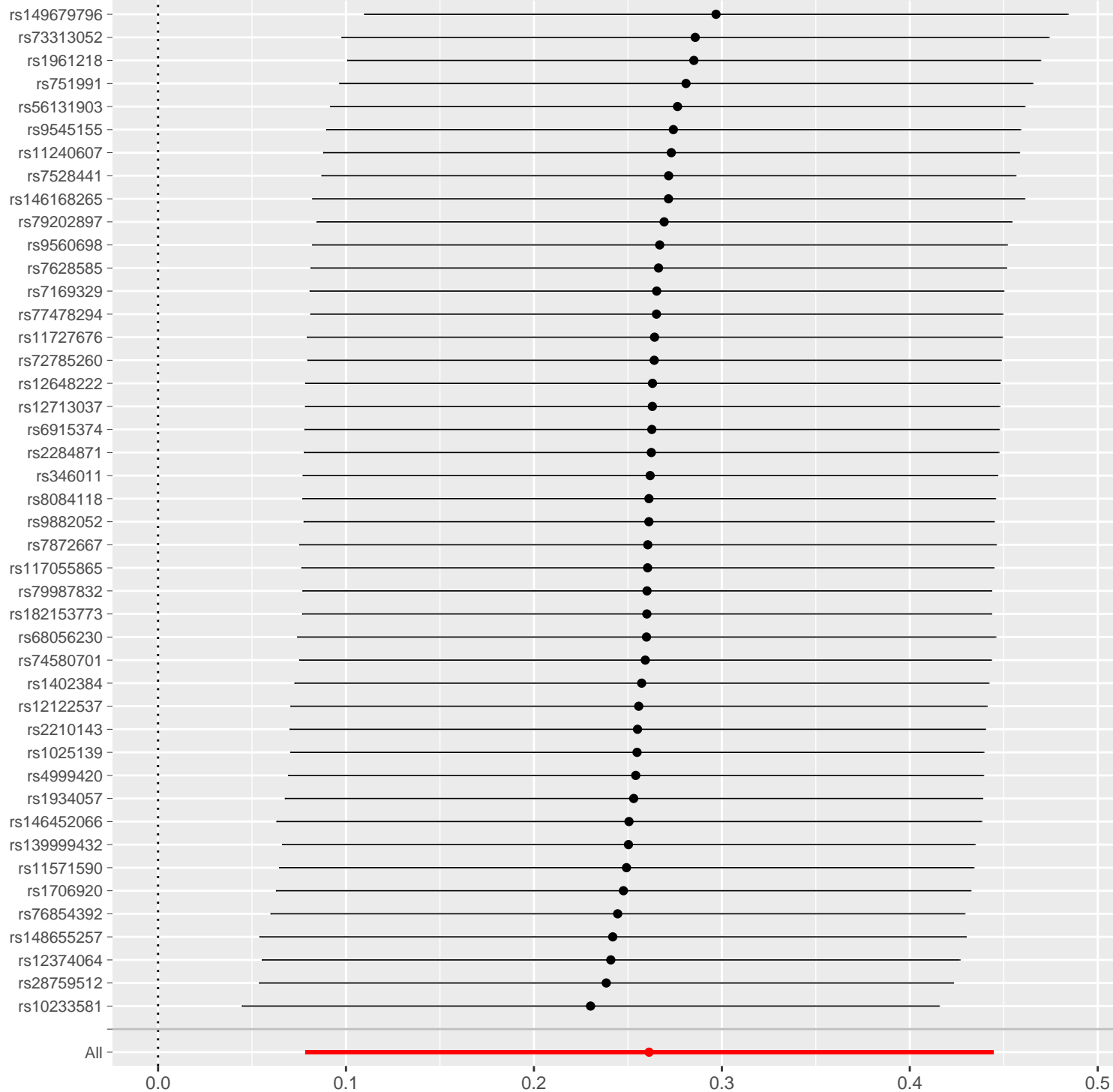

Supplement: Supplementary file 7 — Data S3: Supporting Information. [file ADB-31-e70160-s008.zip › Additional file3/Forward MR analysis/GCST90002873.pdf]

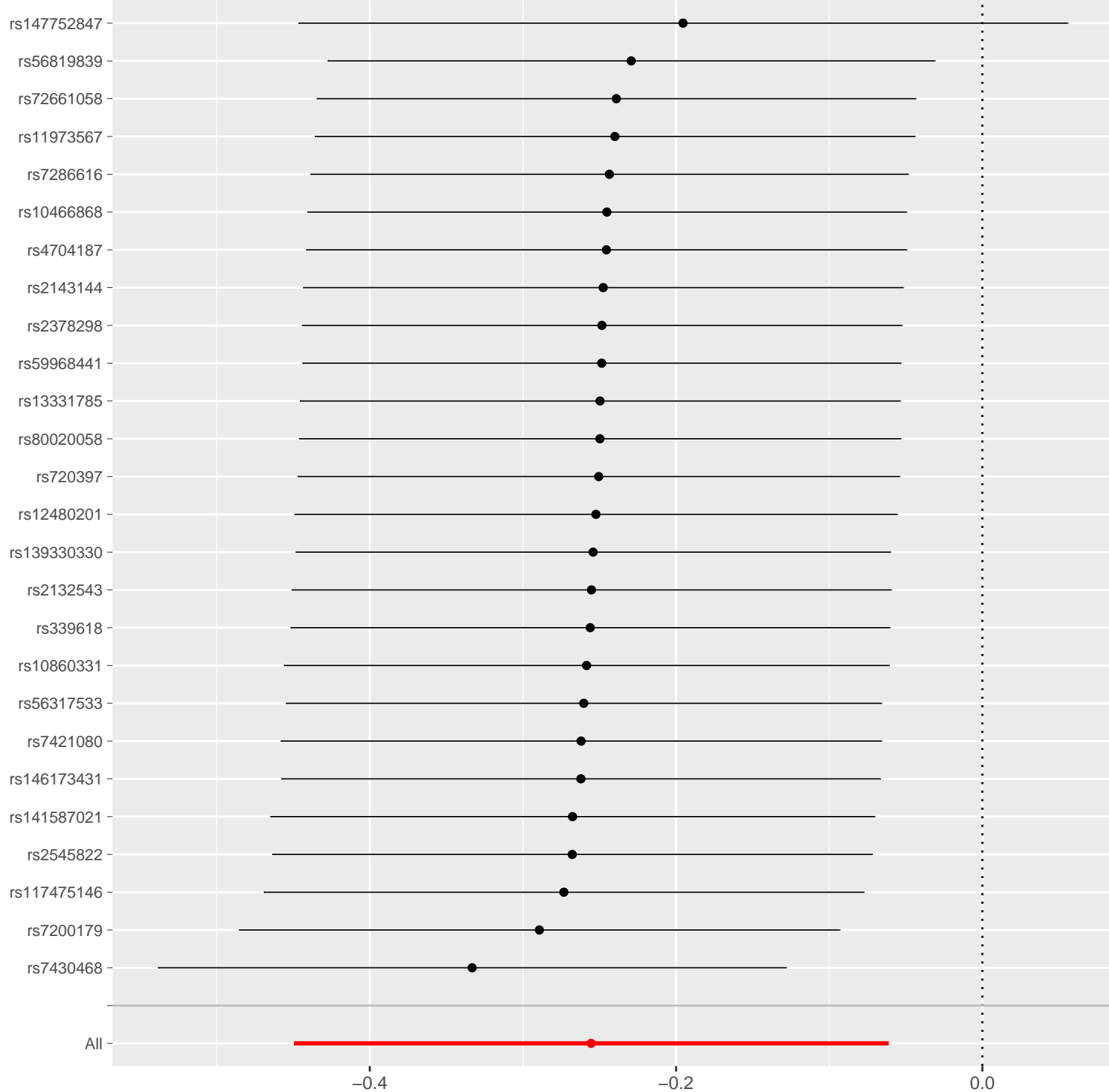

Supplement: Supplementary file 7 — Data S3: Supporting Information. [file ADB-31-e70160-s008.zip › Additional file3/Forward MR analysis/GCST90005844.pdf]

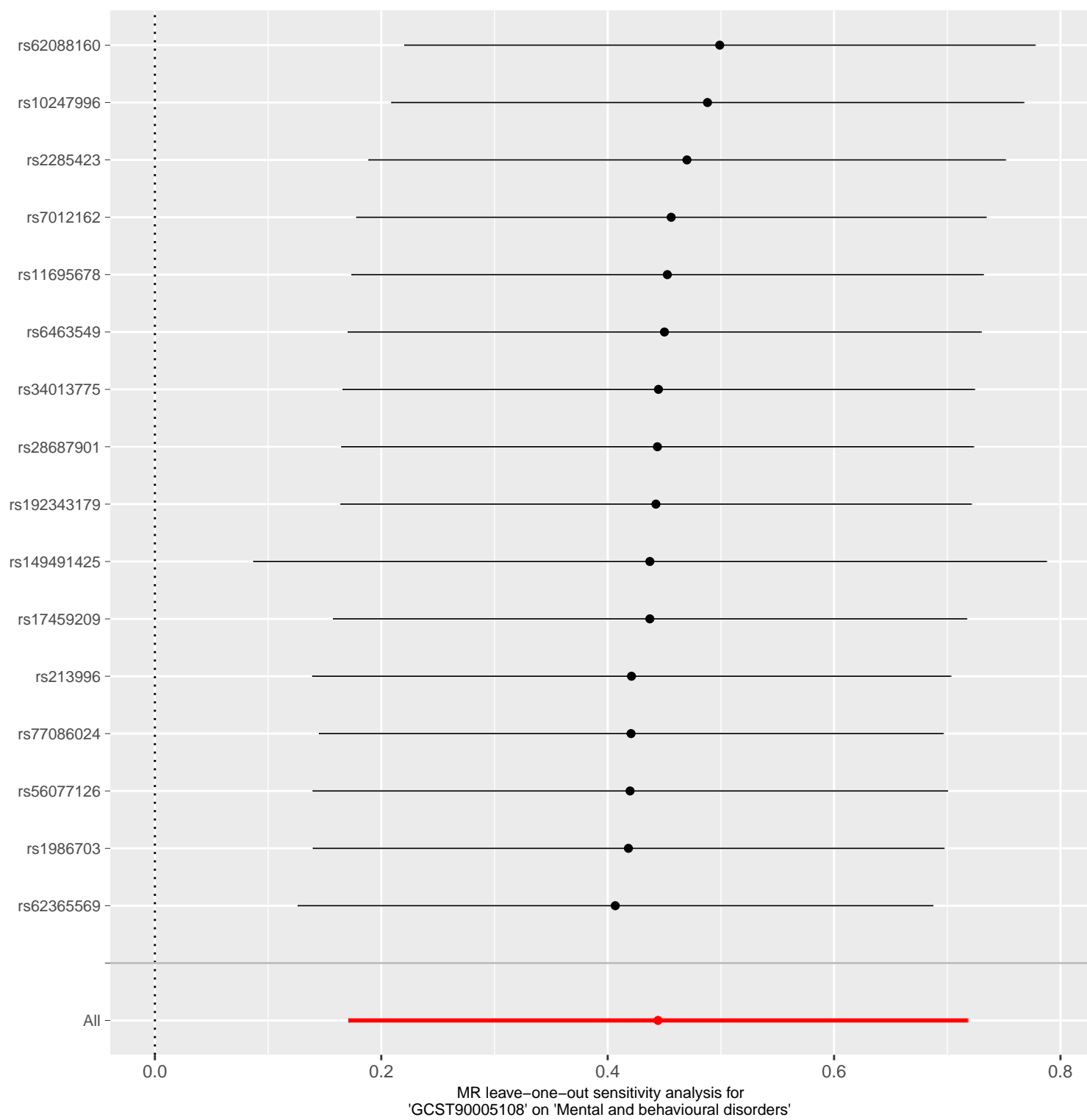

Supplement: Supplementary file 7 — Data S3: Supporting Information. [file ADB-31-e70160-s008.zip › Additional file3/Forward MR analysis/GCST90005108.pdf]

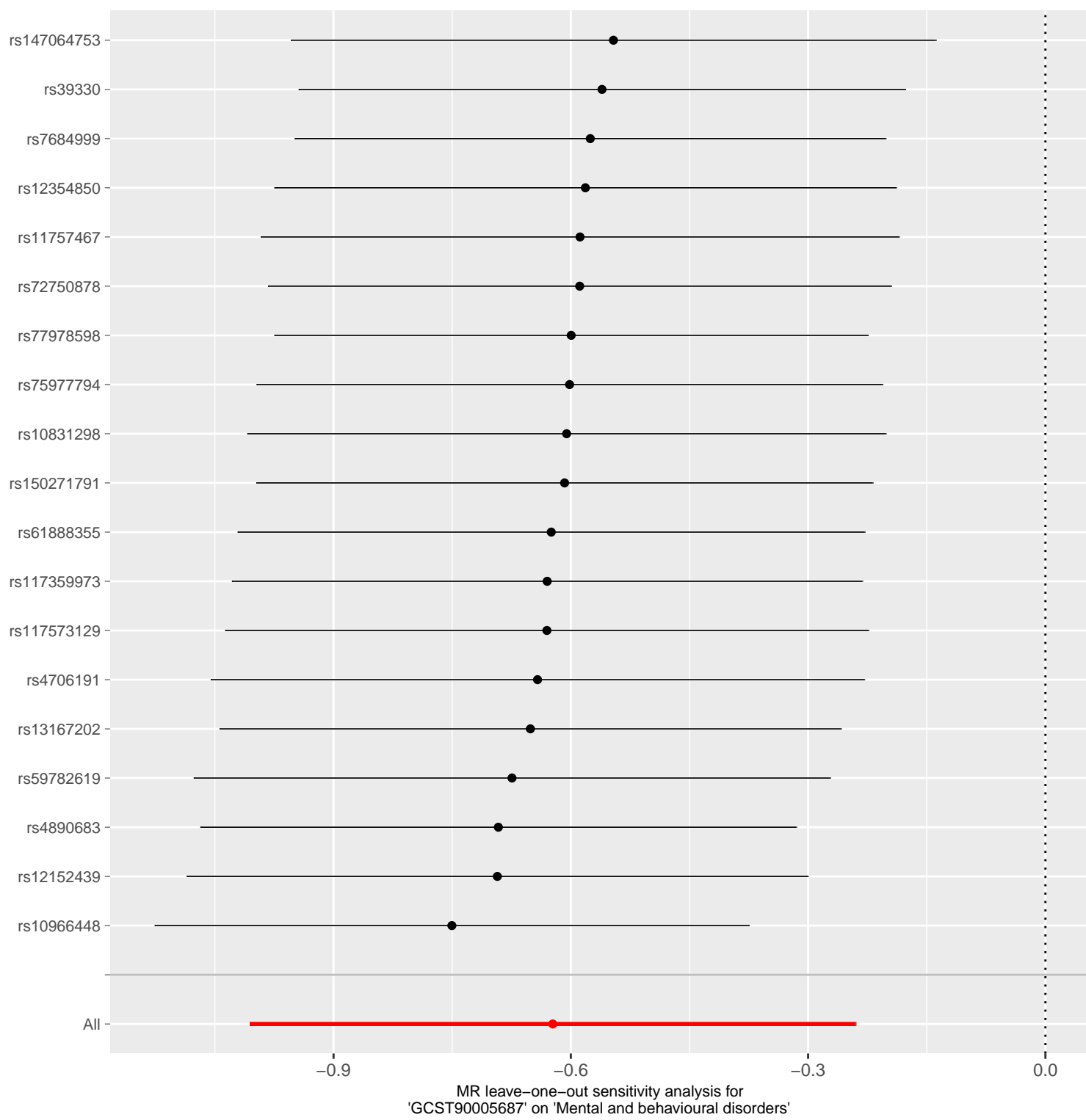

Supplement: Supplementary file 7 — Data S3: Supporting Information. [file ADB-31-e70160-s008.zip › Additional file3/Forward MR analysis/GCST90005687.pdf]

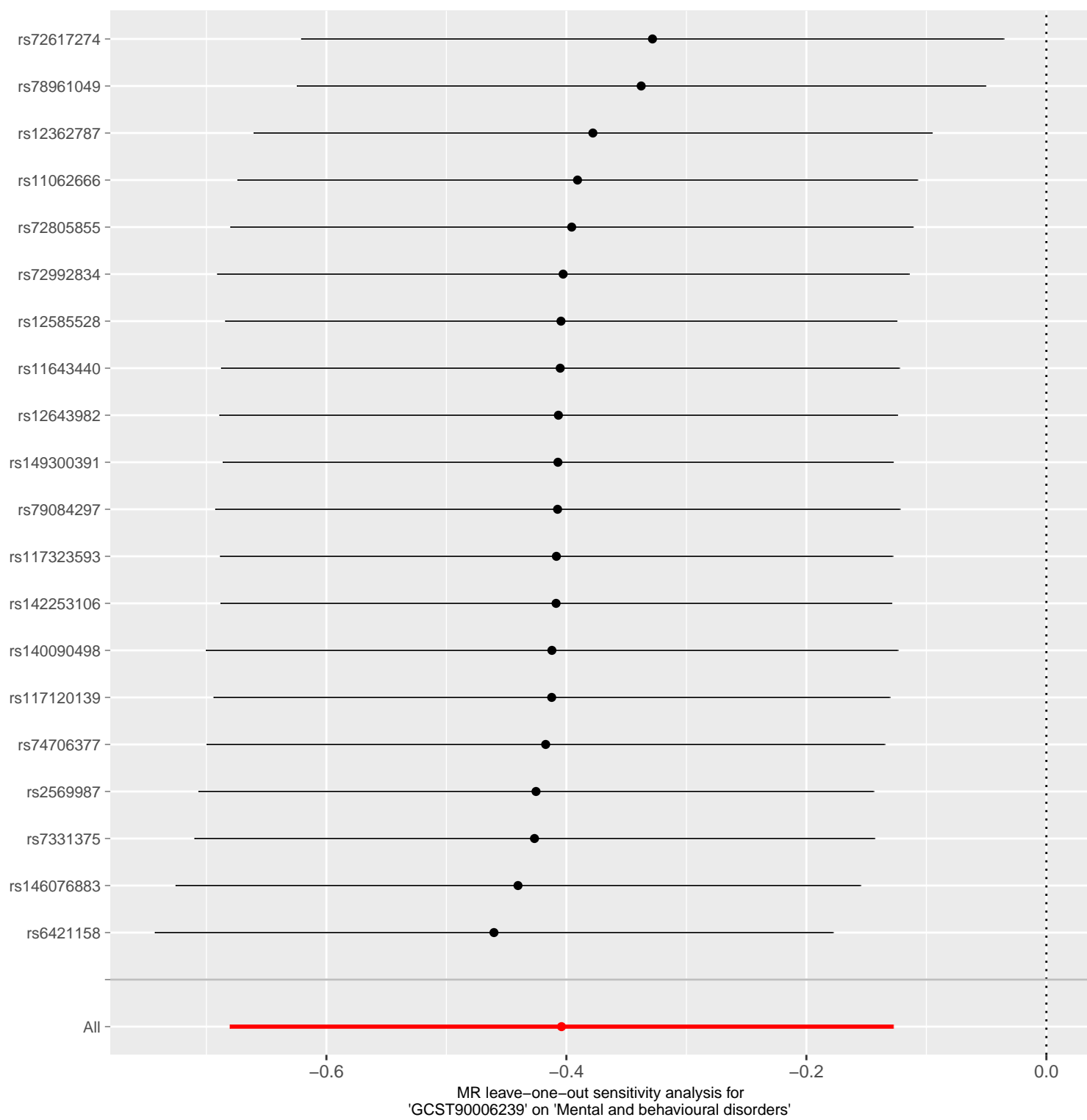

Supplement: Supplementary file 7 — Data S3: Supporting Information. [file ADB-31-e70160-s008.zip › Additional file3/Forward MR analysis/GCST90006239.pdf]

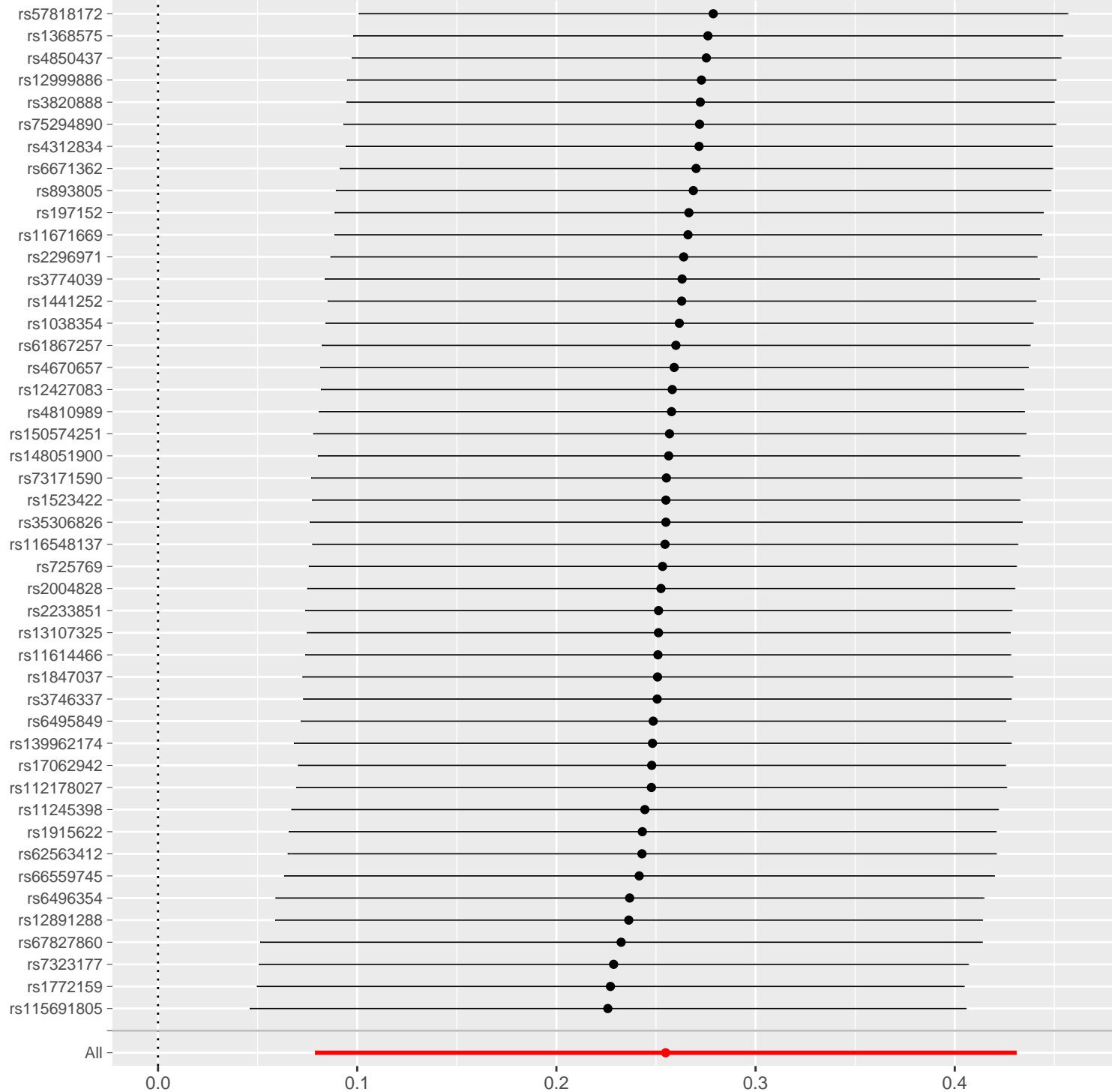

Supplement: Supplementary file 7 — Data S3: Supporting Information. [file ADB-31-e70160-s008.zip › Additional file3/Forward MR analysis/GCST90003807.pdf]

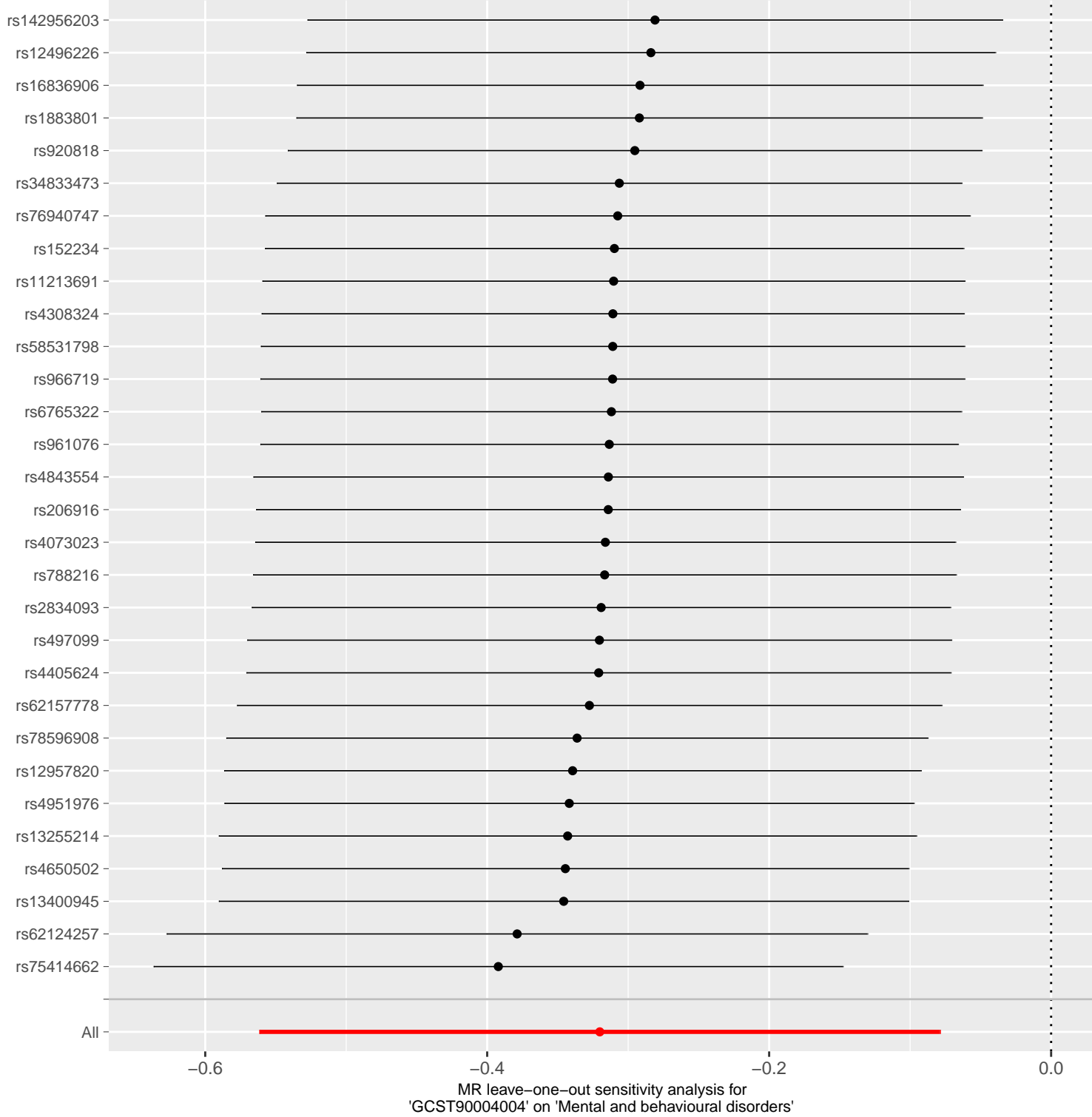

Supplement: Supplementary file 7 — Data S3: Supporting Information. [file ADB-31-e70160-s008.zip › Additional file3/Forward MR analysis/GCST90004004.pdf]

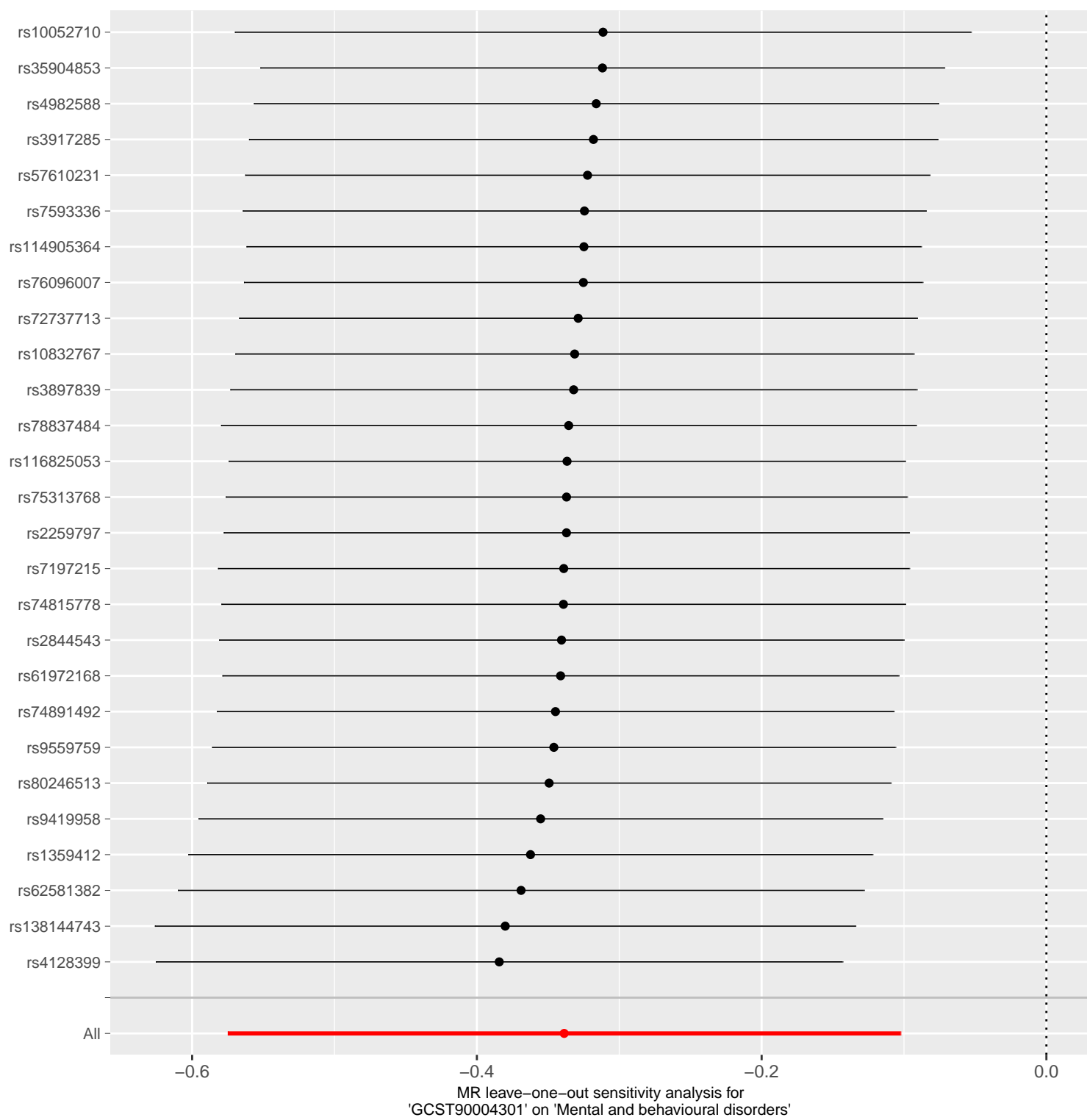

Supplement: Supplementary file 7 — Data S3: Supporting Information. [file ADB-31-e70160-s008.zip › Additional file3/Forward MR analysis/GCST90004301.pdf]

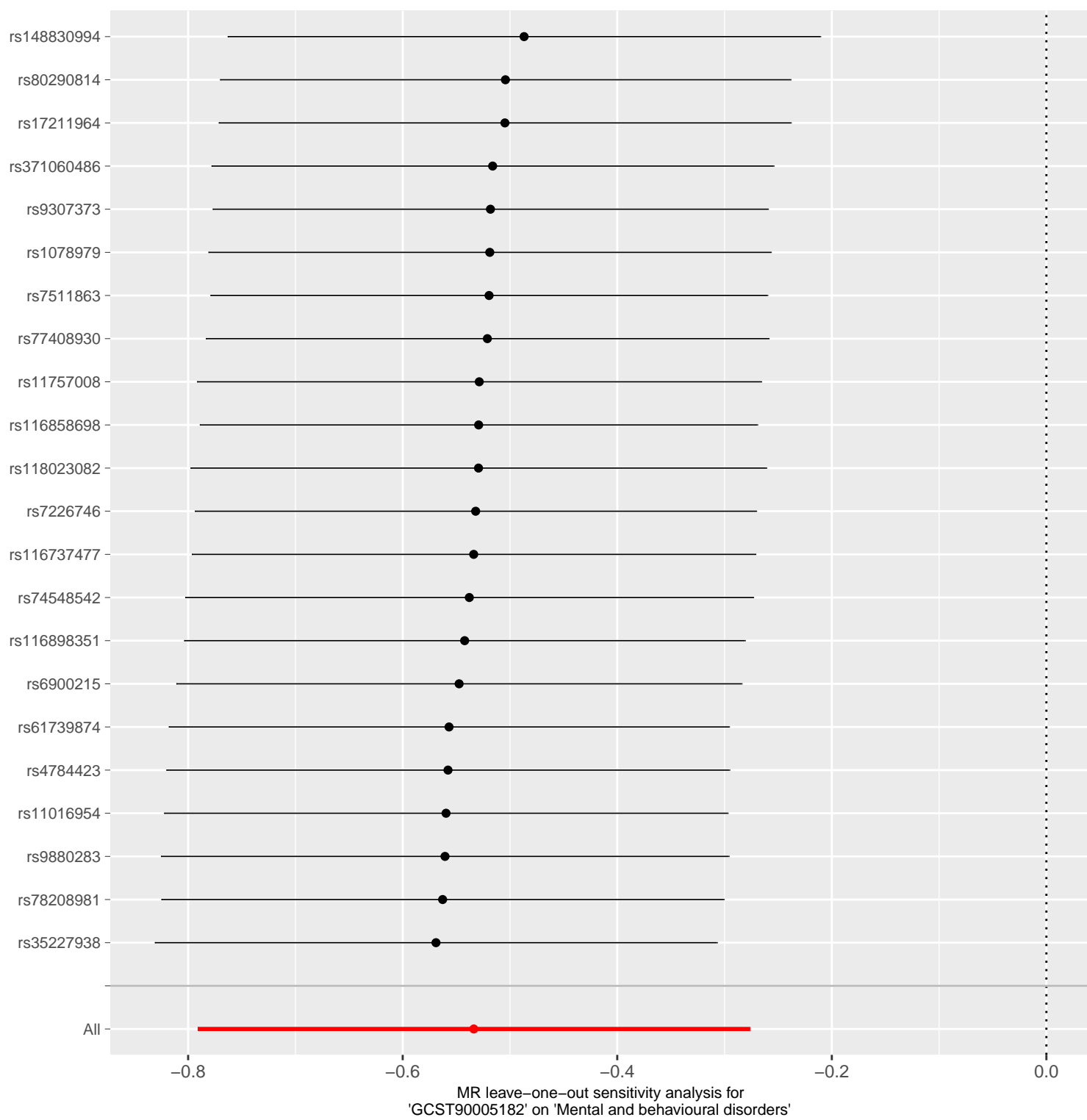

Supplement: Supplementary file 7 — Data S3: Supporting Information. [file ADB-31-e70160-s008.zip › Additional file3/Forward MR analysis/GCST90005182.pdf]

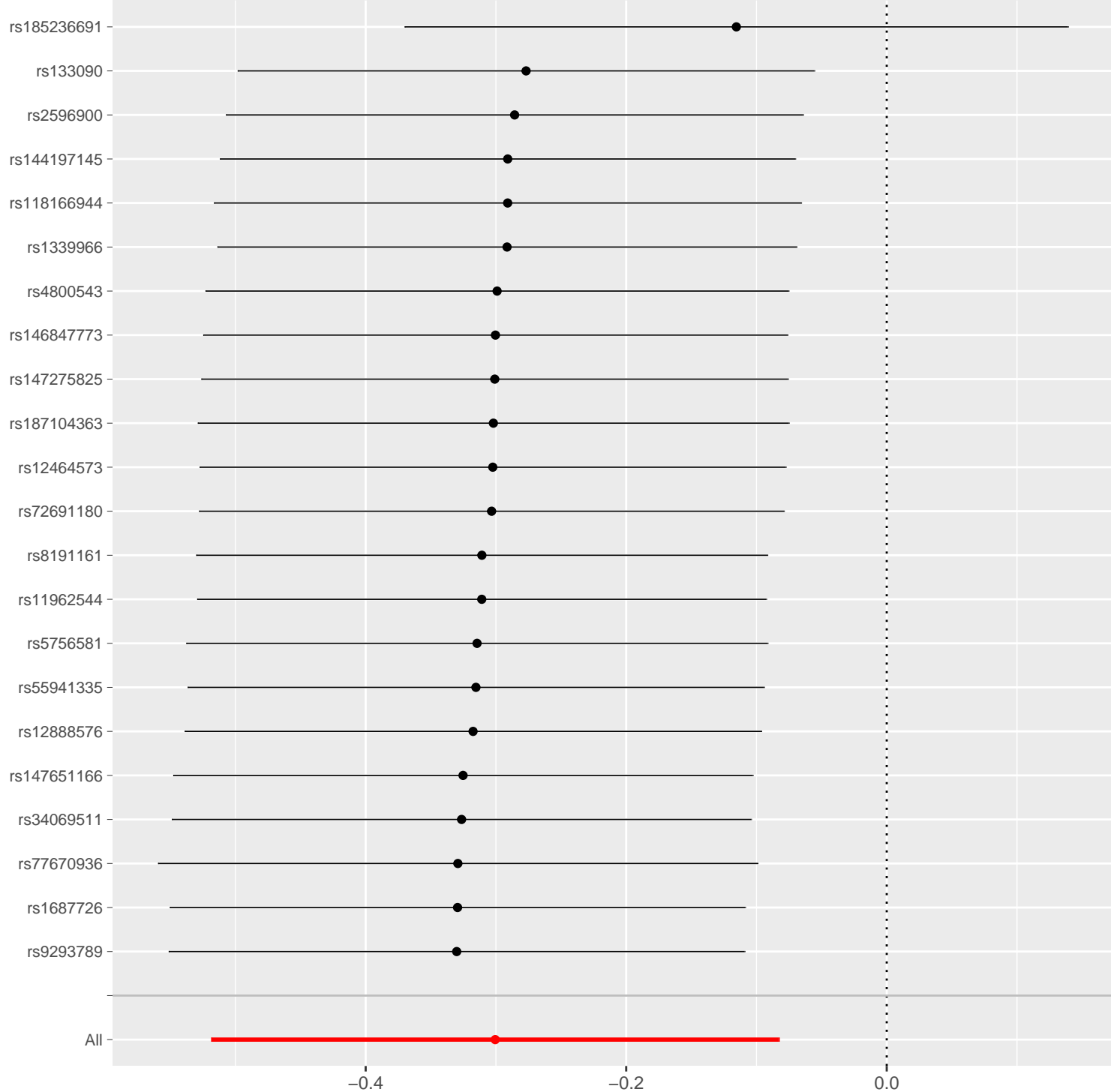

Supplement: Supplementary file 7 — Data S3: Supporting Information. [file ADB-31-e70160-s008.zip › Additional file3/Forward MR analysis/GCST90006306.pdf]

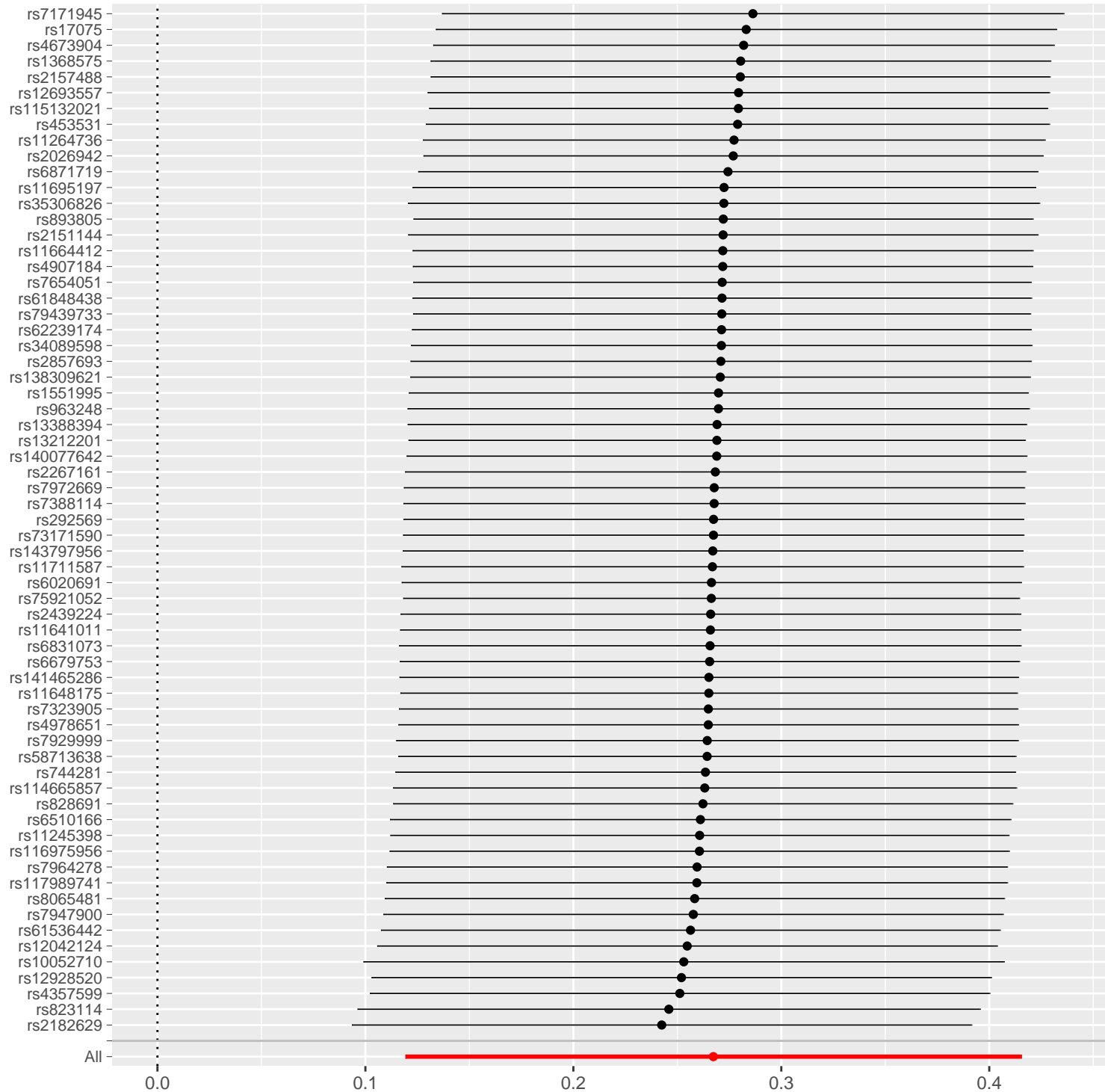

Supplement: Supplementary file 7 — Data S3: Supporting Information. [file ADB-31-e70160-s008.zip › Additional file3/Forward MR analysis/GCST90003861.pdf]

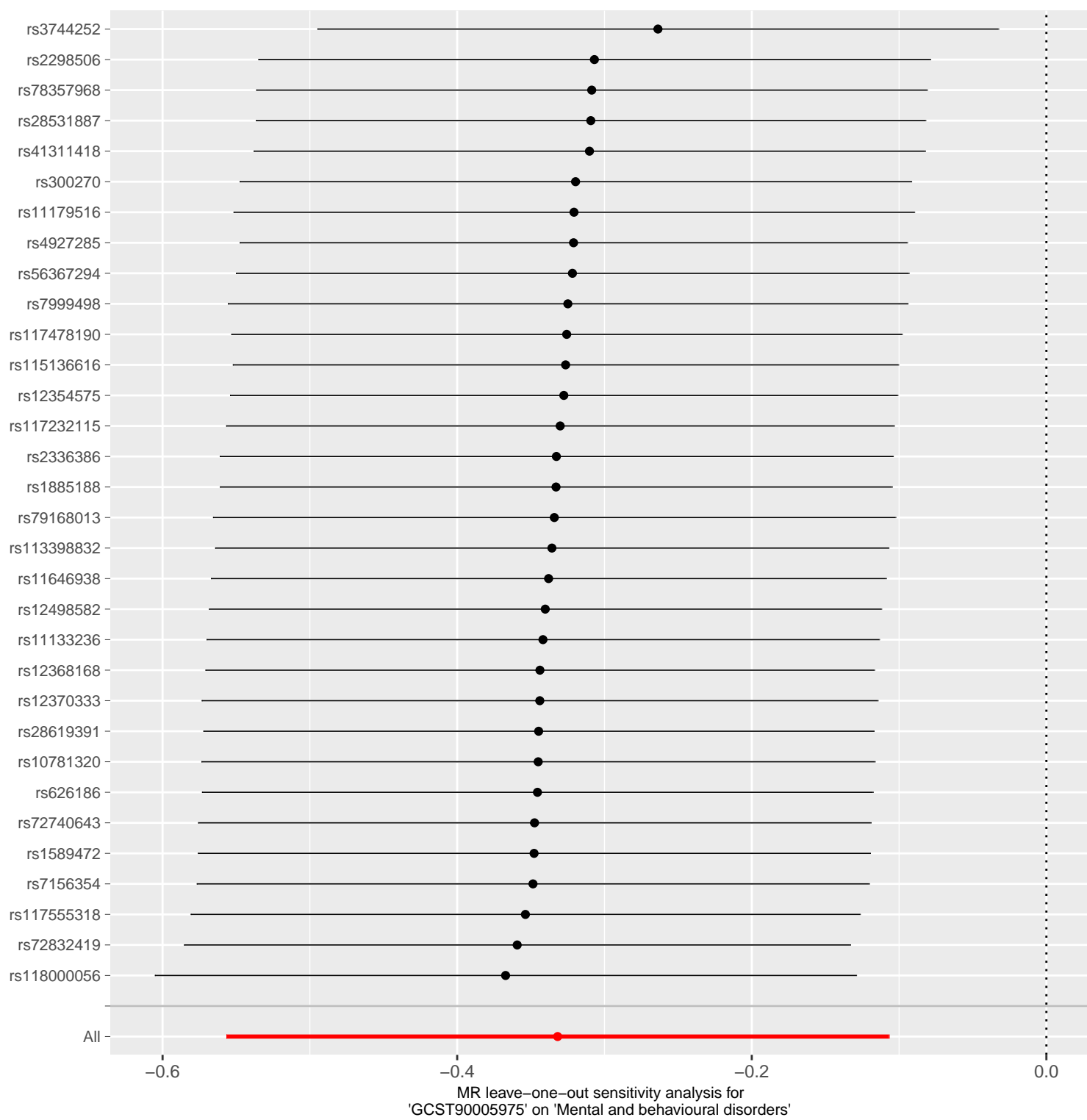

Supplement: Supplementary file 7 — Data S3: Supporting Information. [file ADB-31-e70160-s008.zip › Additional file3/Forward MR analysis/GCST90005975.pdf]

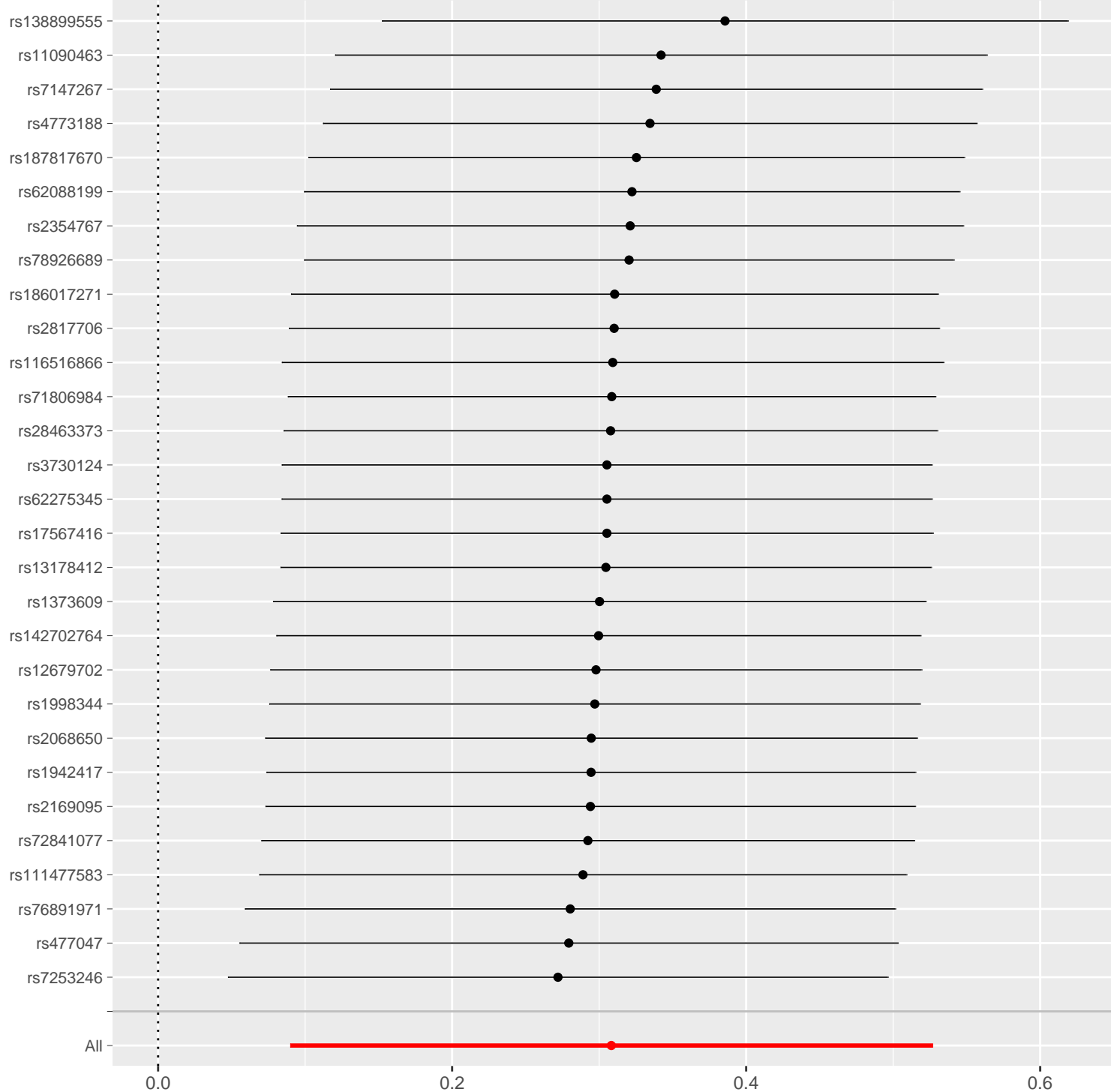

MR leave-one-out sensitivity analysis for  
'GCST90003733' on 'Mental and behavioural disorders'

Supplement: Supplementary file 7 — Data S3: Supporting Information. [file ADB-31-e70160-s008.zip › Additional file3/Forward MR analysis/GCST90003733.pdf]

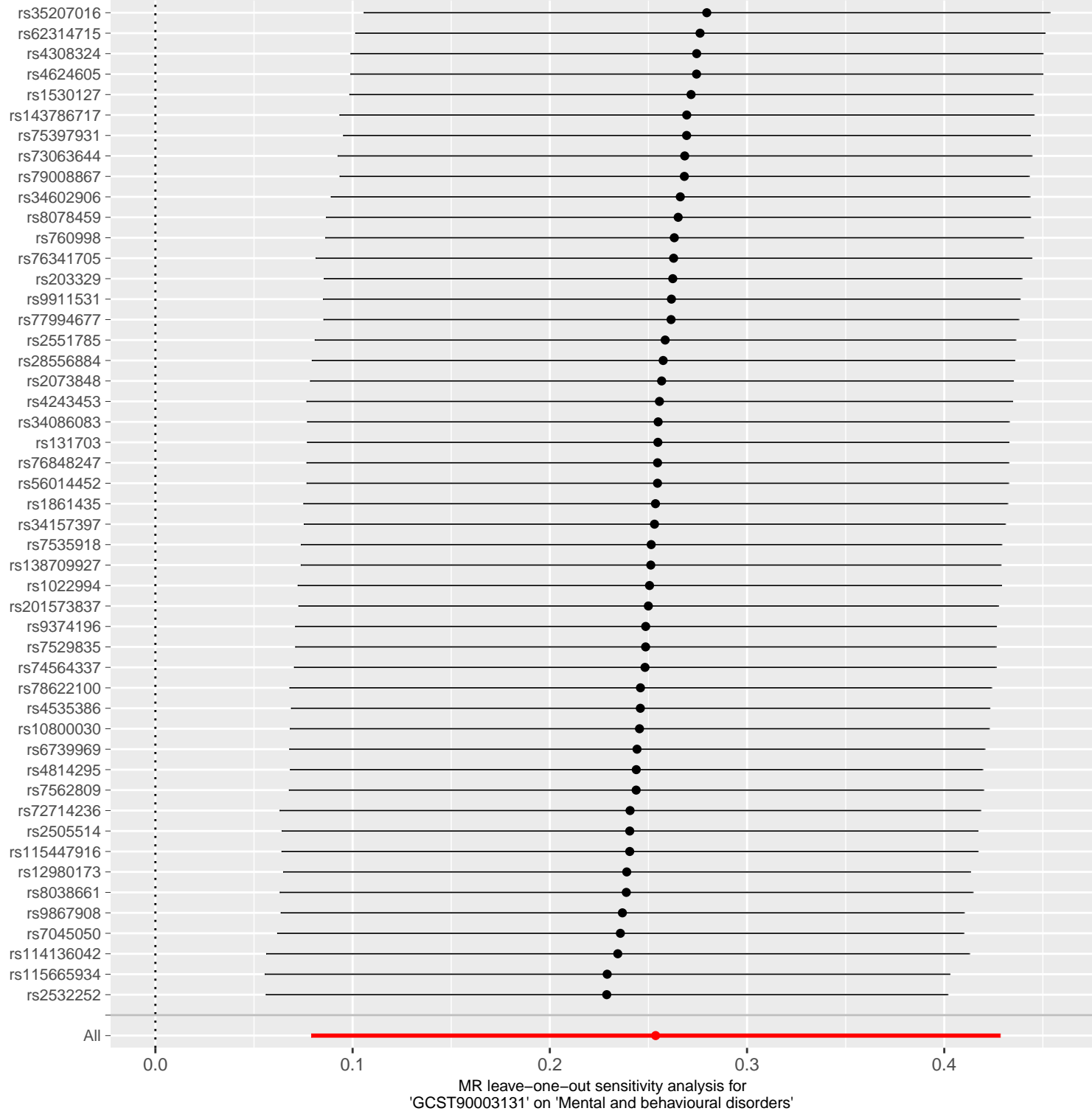

Supplement: Supplementary file 7 — Data S3: Supporting Information. [file ADB-31-e70160-s008.zip › Additional file3/Forward MR analysis/GCST90003131.pdf]

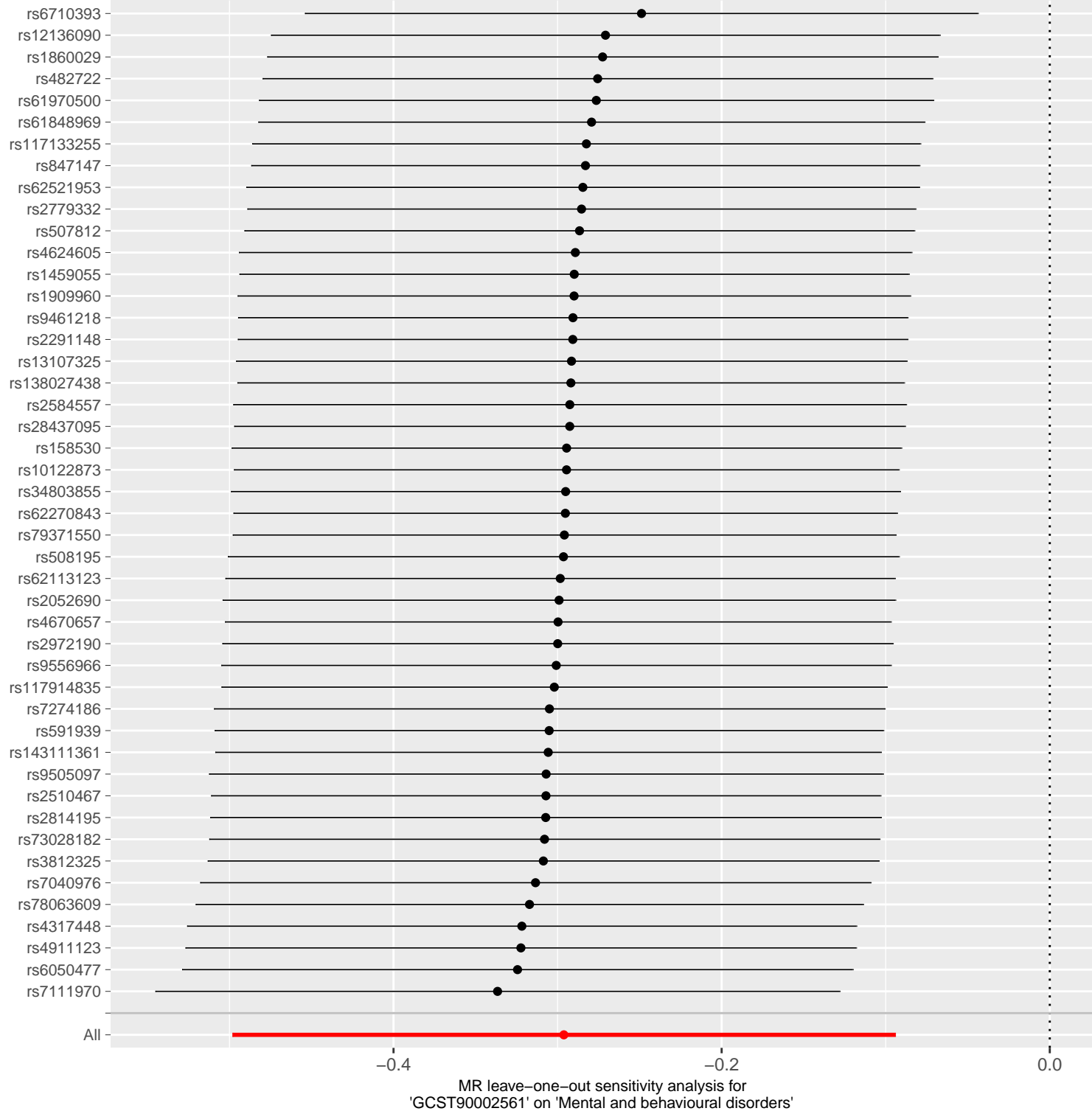

Supplement: Supplementary file 7 — Data S3: Supporting Information. [file ADB-31-e70160-s008.zip › Additional file3/Forward MR analysis/GCST90002561.pdf]

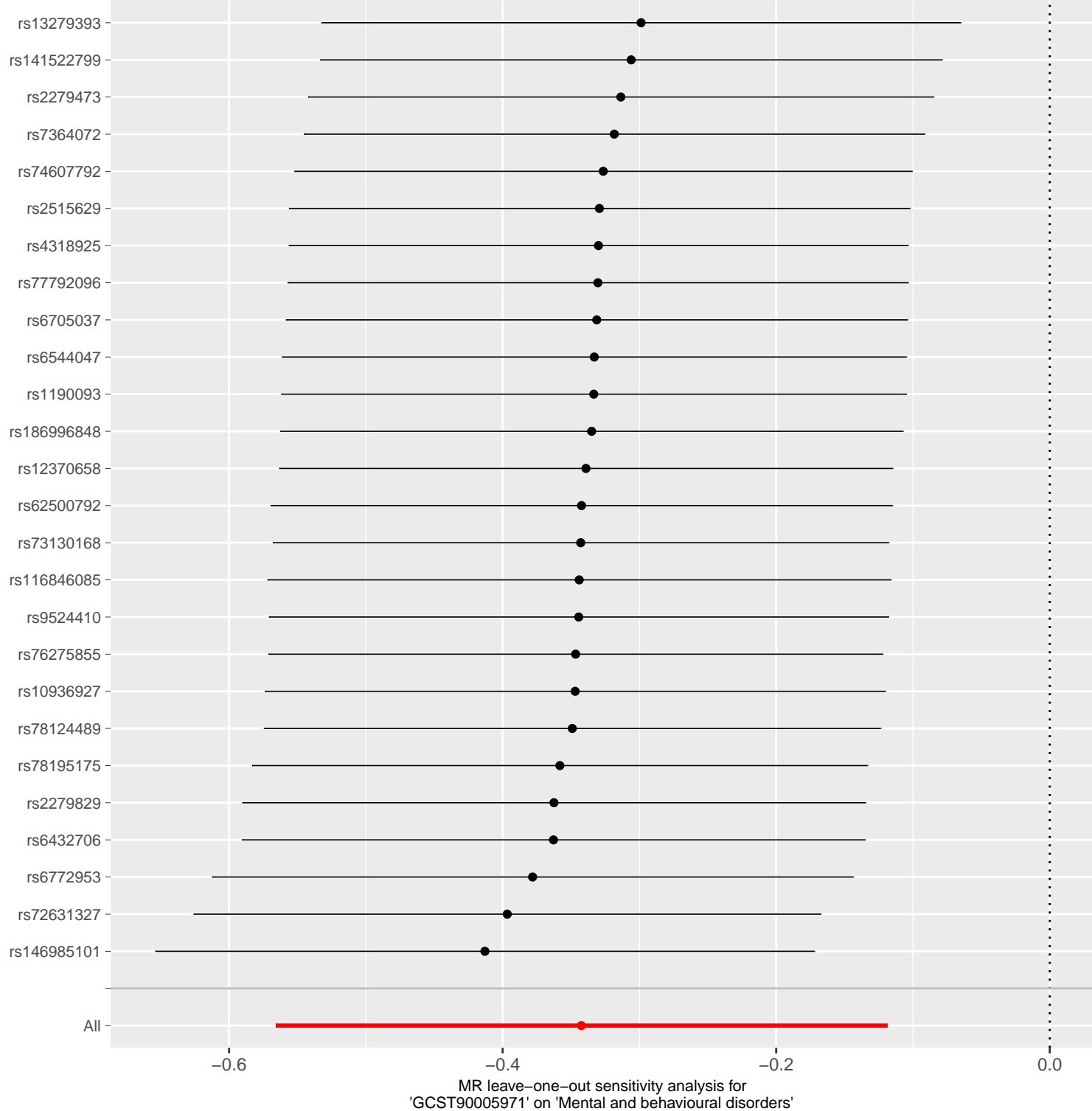

Supplement: Supplementary file 7 — Data S3: Supporting Information. [file ADB-31-e70160-s008.zip › Additional file3/Forward MR analysis/GCST90005971.pdf]

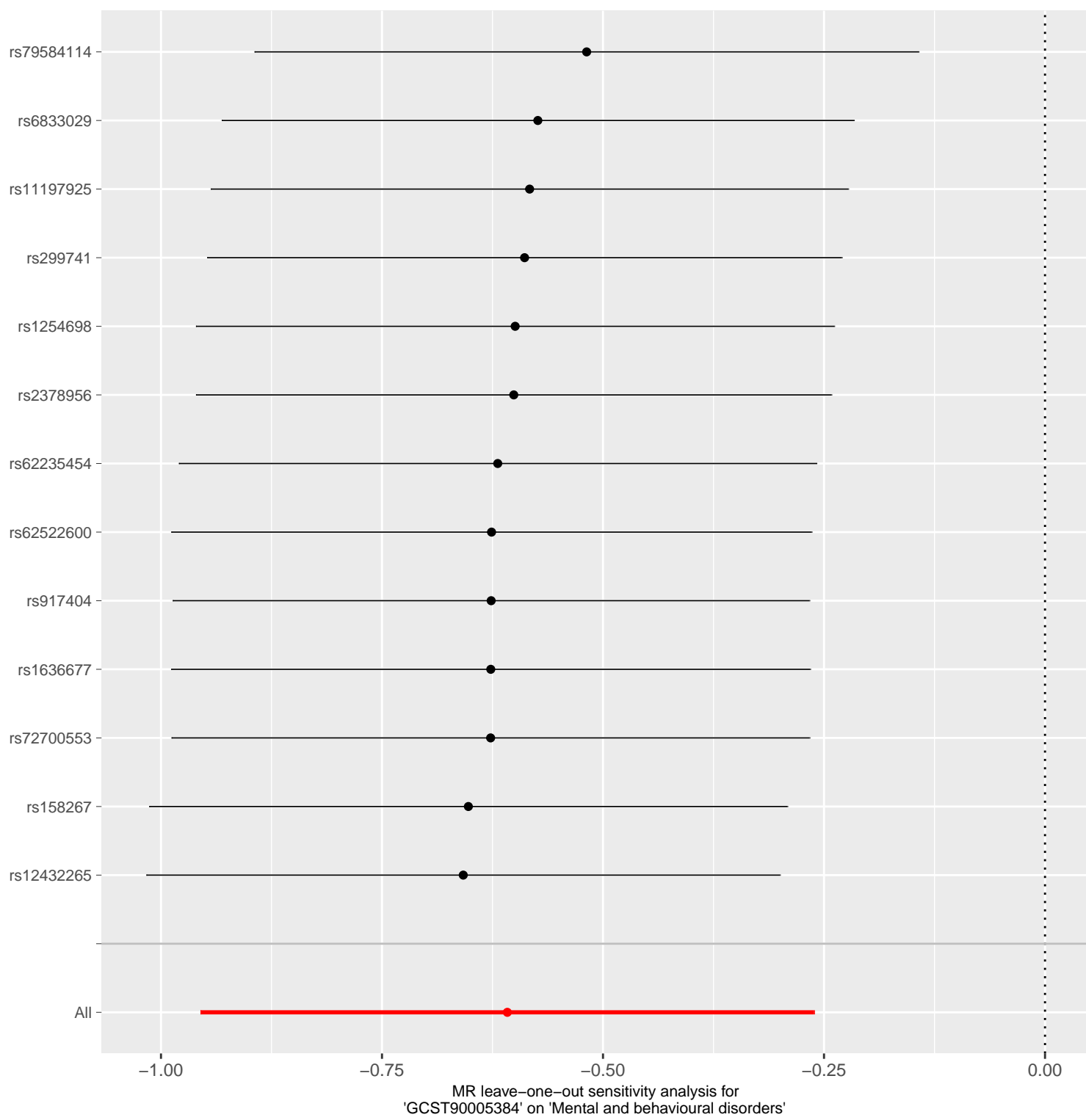

Supplement: Supplementary file 7 — Data S3: Supporting Information. [file ADB-31-e70160-s008.zip › Additional file3/Forward MR analysis/GCST90005384.pdf]

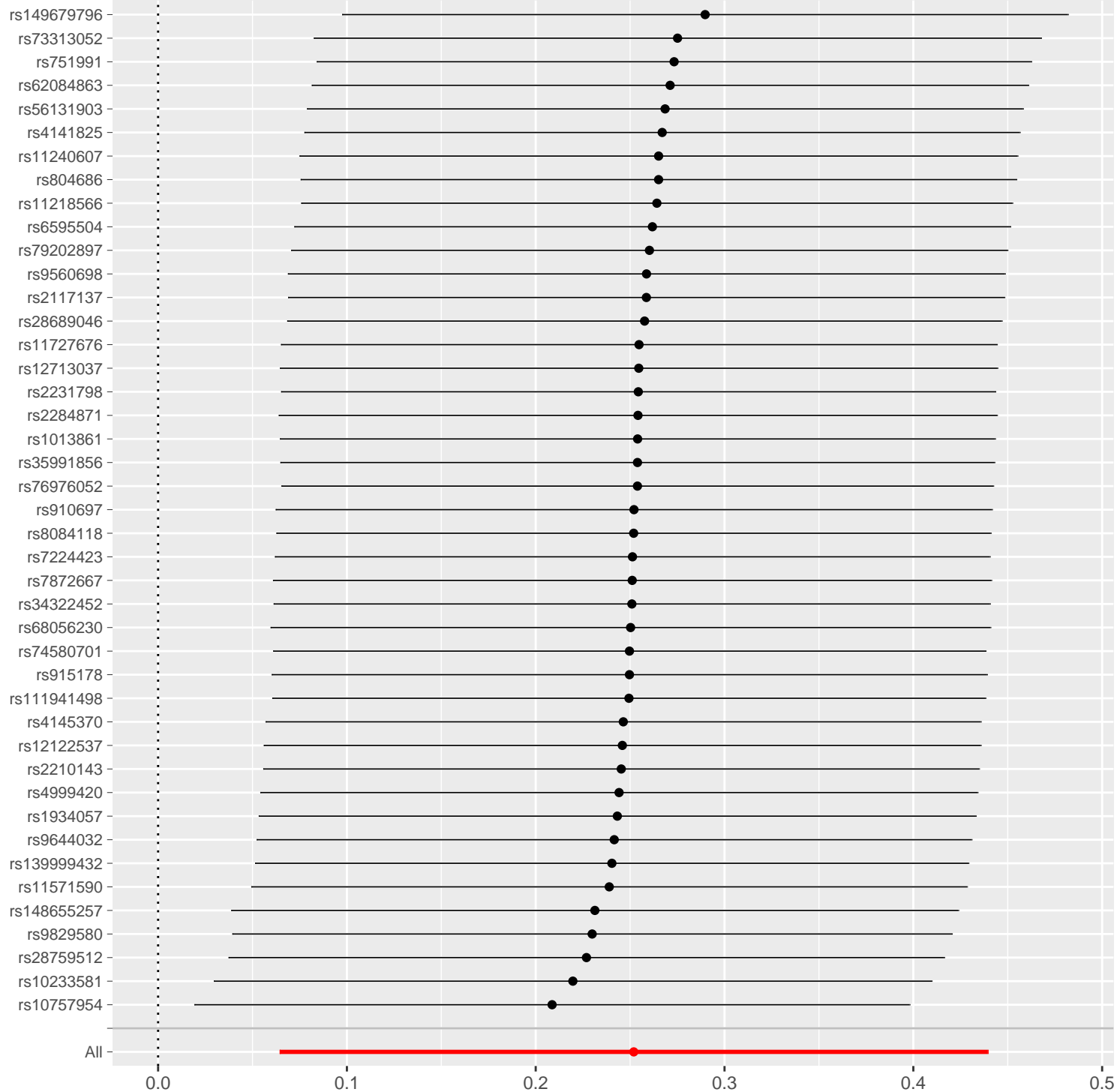

MR leave-one-out sensitivity analysis for  
'GCST90002946' on 'Mental and behavioural disorders'

Supplement: Supplementary file 7 — Data S3: Supporting Information. [file ADB-31-e70160-s008.zip › Additional file3/Forward MR analysis/GCST90002946.pdf]

# MR Method

- Inverse variance weighted
- MR Egger

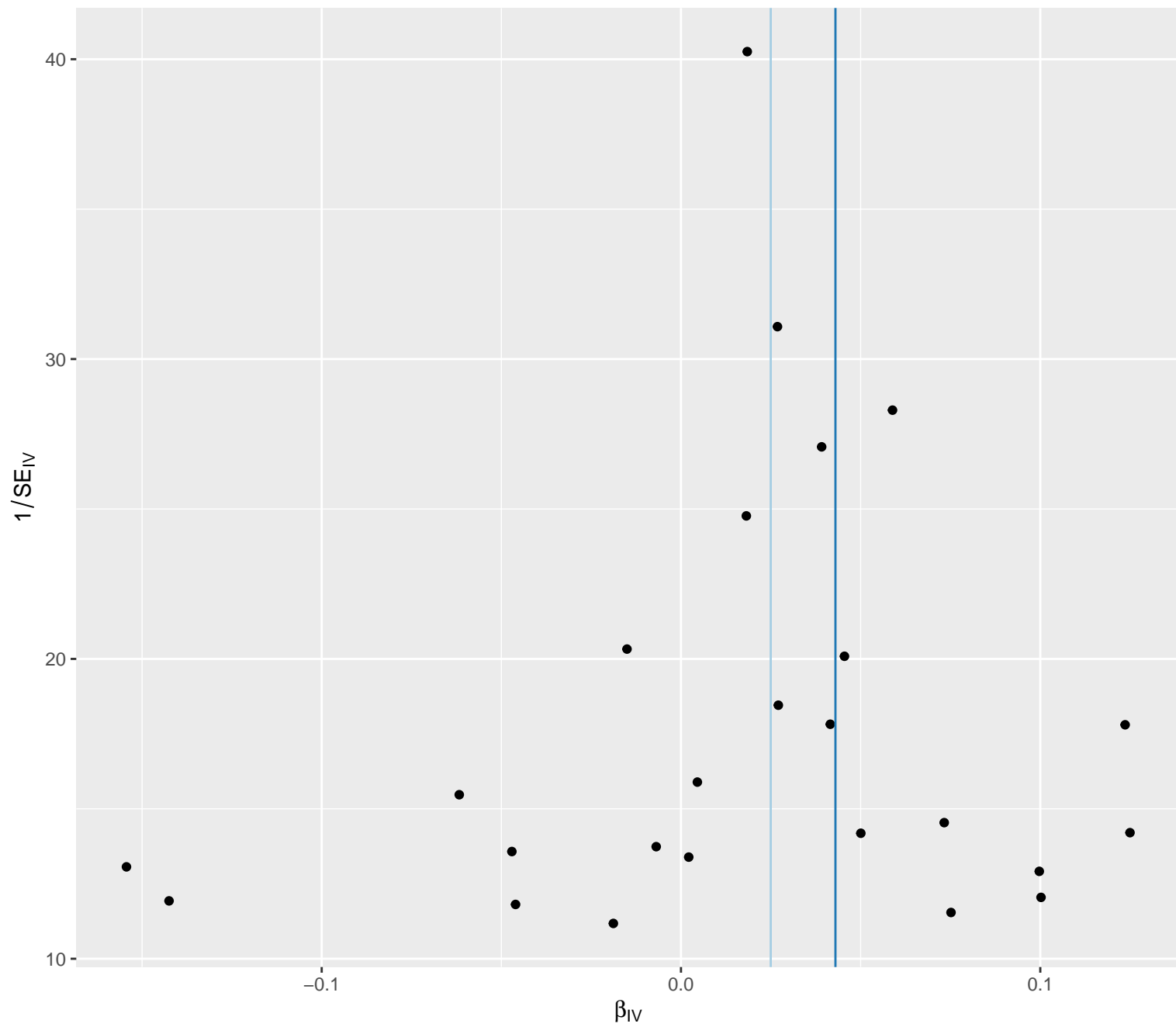

Supplement: Supplementary file 8 — Data S4: Supporting Information. [file ADB-31-e70160-s001.zip › Additional file4/Reverse MR analysis.pdf]

# MR Method

- Inverse variance weighted
- MR Egger

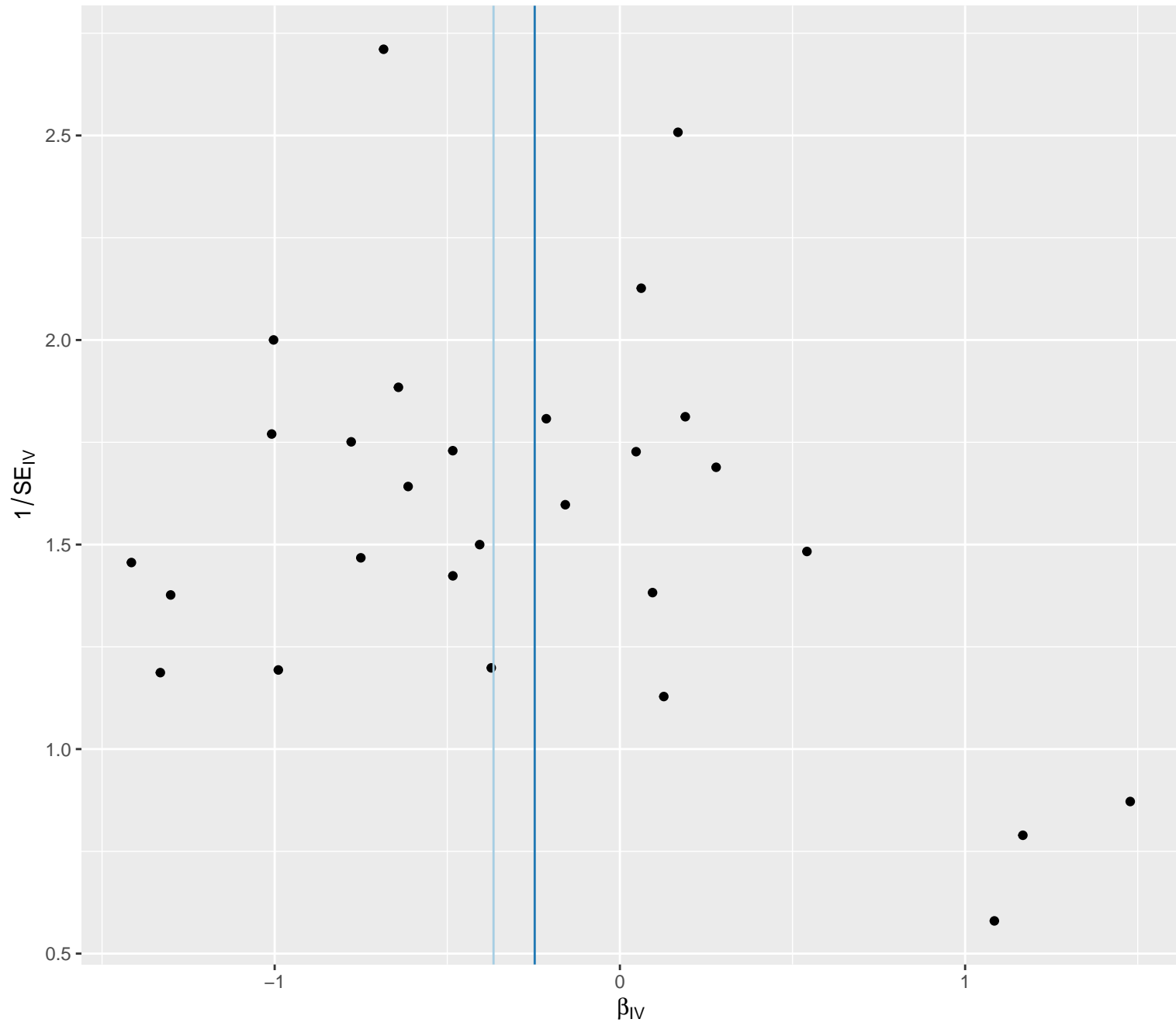

Supplement: Supplementary file 8 — Data S4: Supporting Information. [file ADB-31-e70160-s001.zip › Additional file4/Forward MR analysis/GCST90004862.pdf]

# MR Method

- Inverse variance weighted
- MR Egger

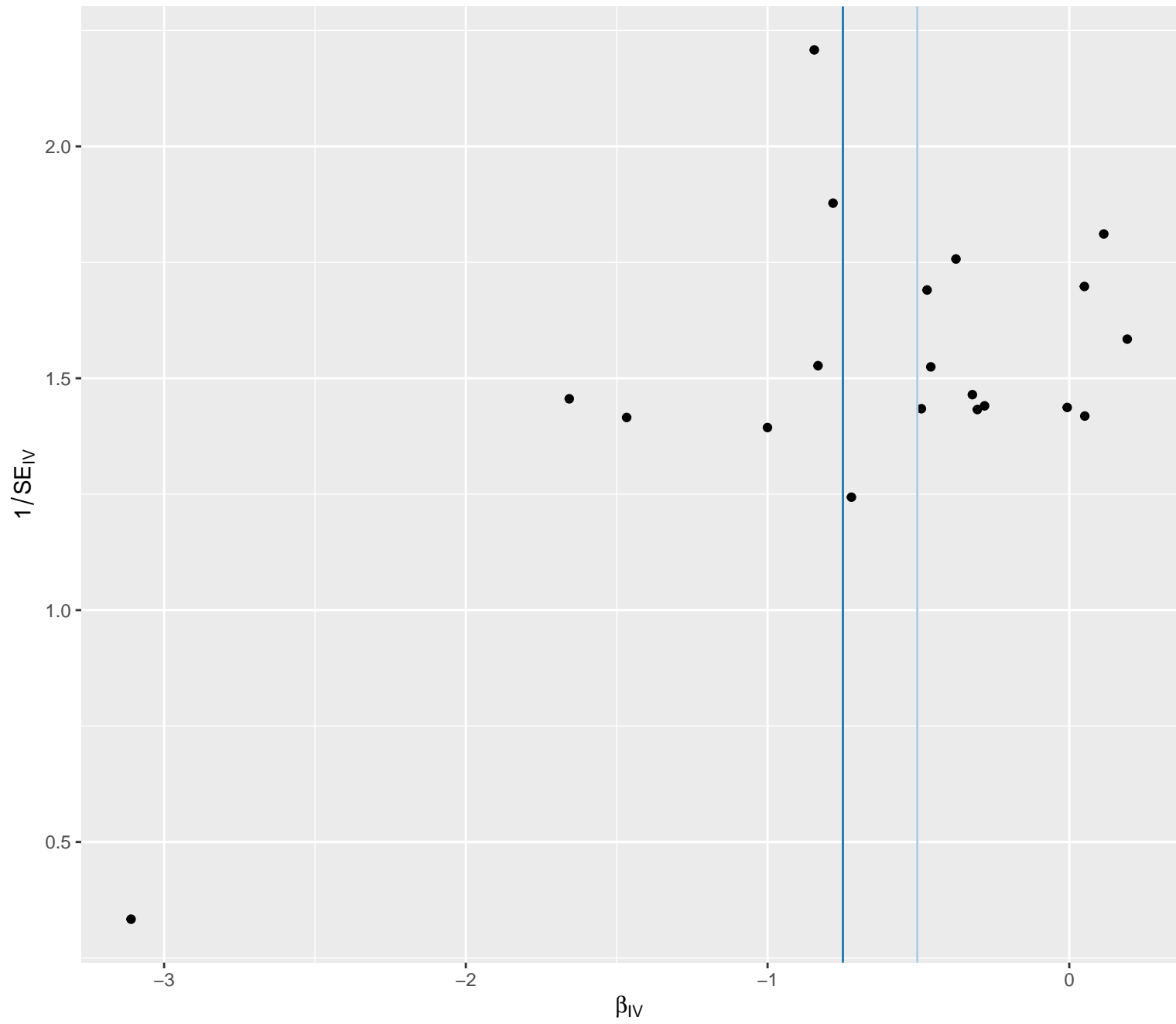

Supplement: Supplementary file 8 — Data S4: Supporting Information. [file ADB-31-e70160-s001.zip › Additional file4/Forward MR analysis/GCST90006319.pdf]

# MR Method

Inverse variance weighted

MR Egger

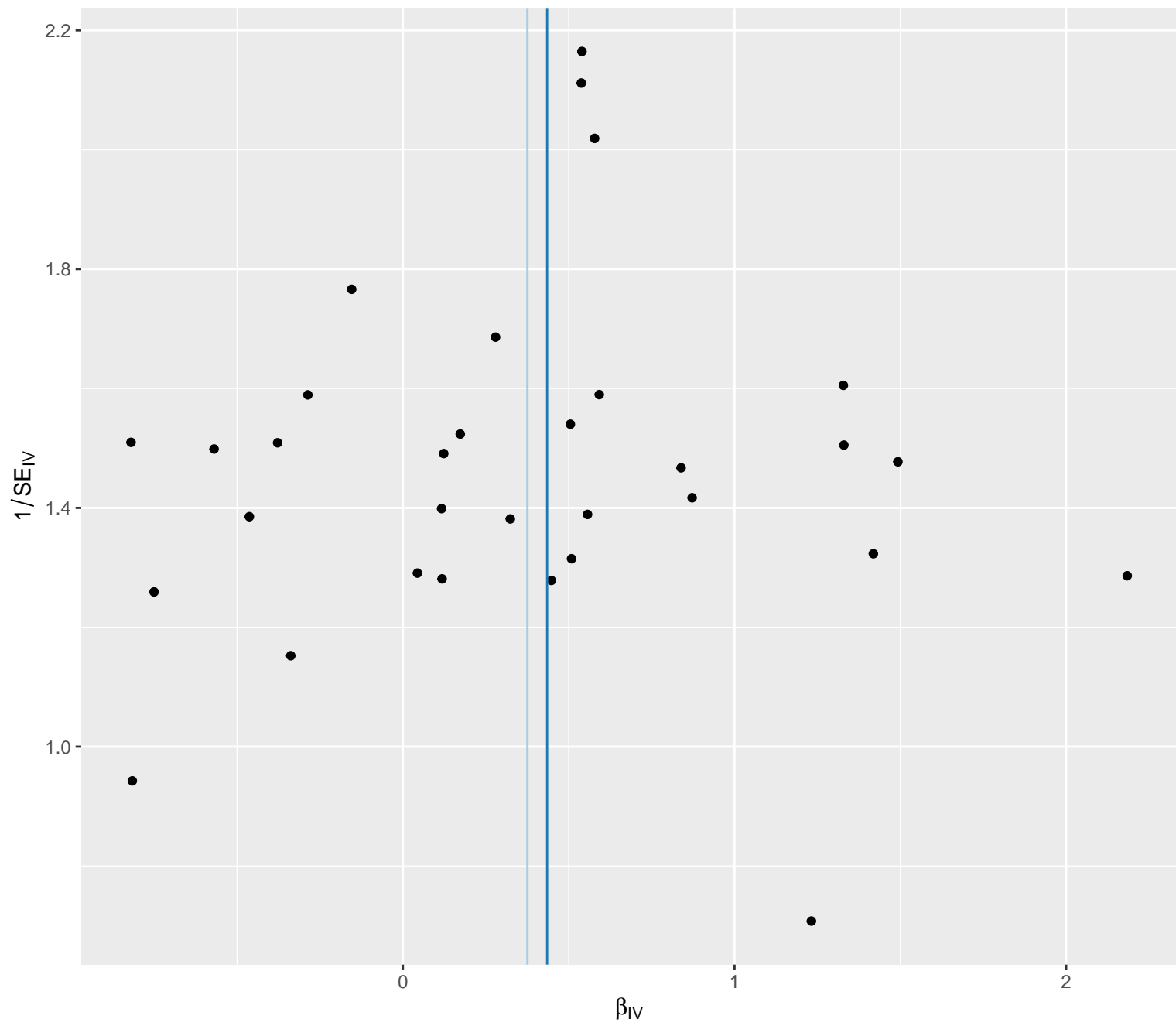

Supplement: Supplementary file 8 — Data S4: Supporting Information. [file ADB-31-e70160-s001.zip › Additional file4/Forward MR analysis/GCST90003058.pdf]

# MR Method

- Inverse variance weighted
- MR Egger

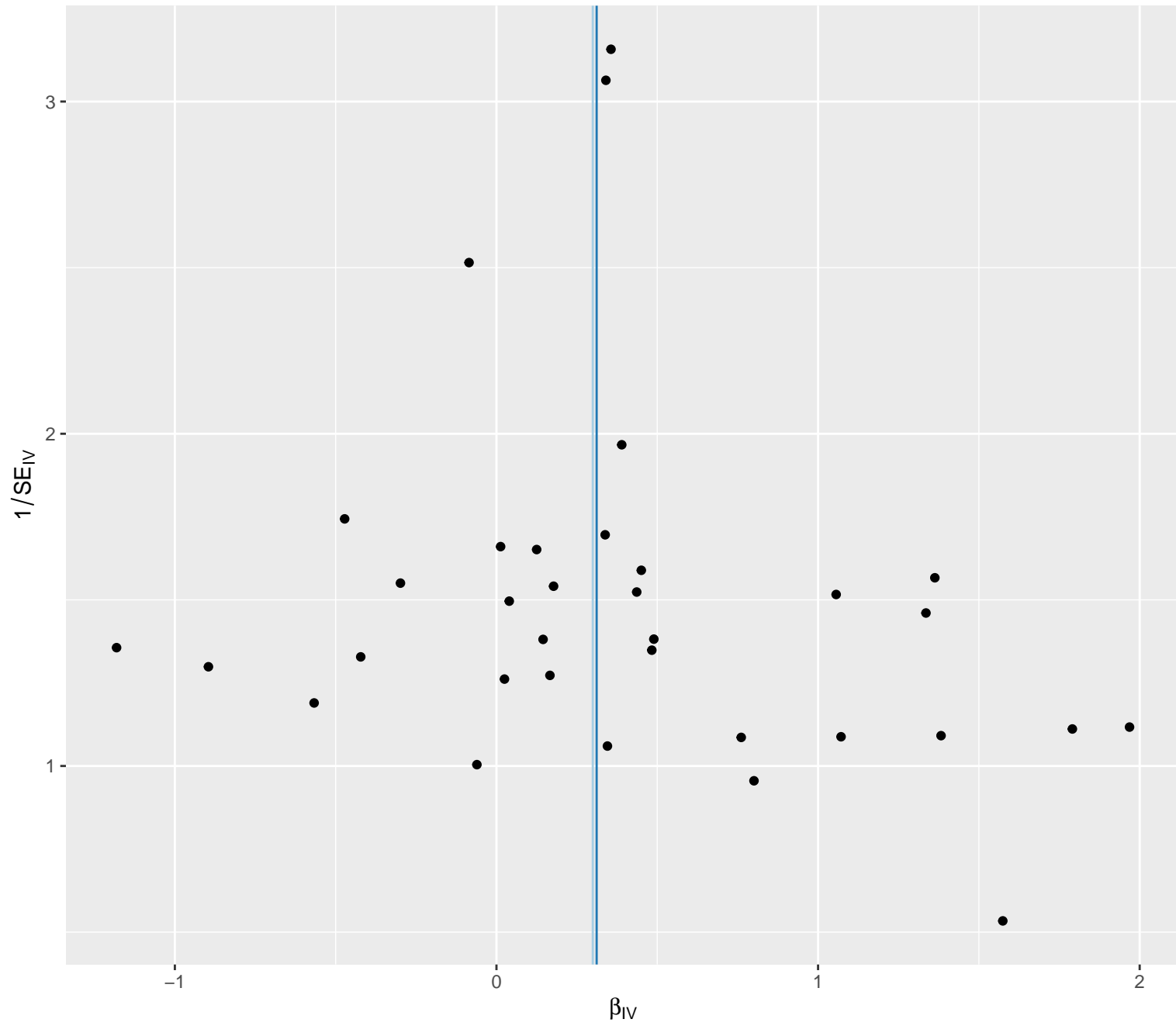

Supplement: Supplementary file 8 — Data S4: Supporting Information. [file ADB-31-e70160-s001.zip › Additional file4/Forward MR analysis/GCST90003676.pdf]

# MR Method

- Inverse variance weighted
- MR Egger

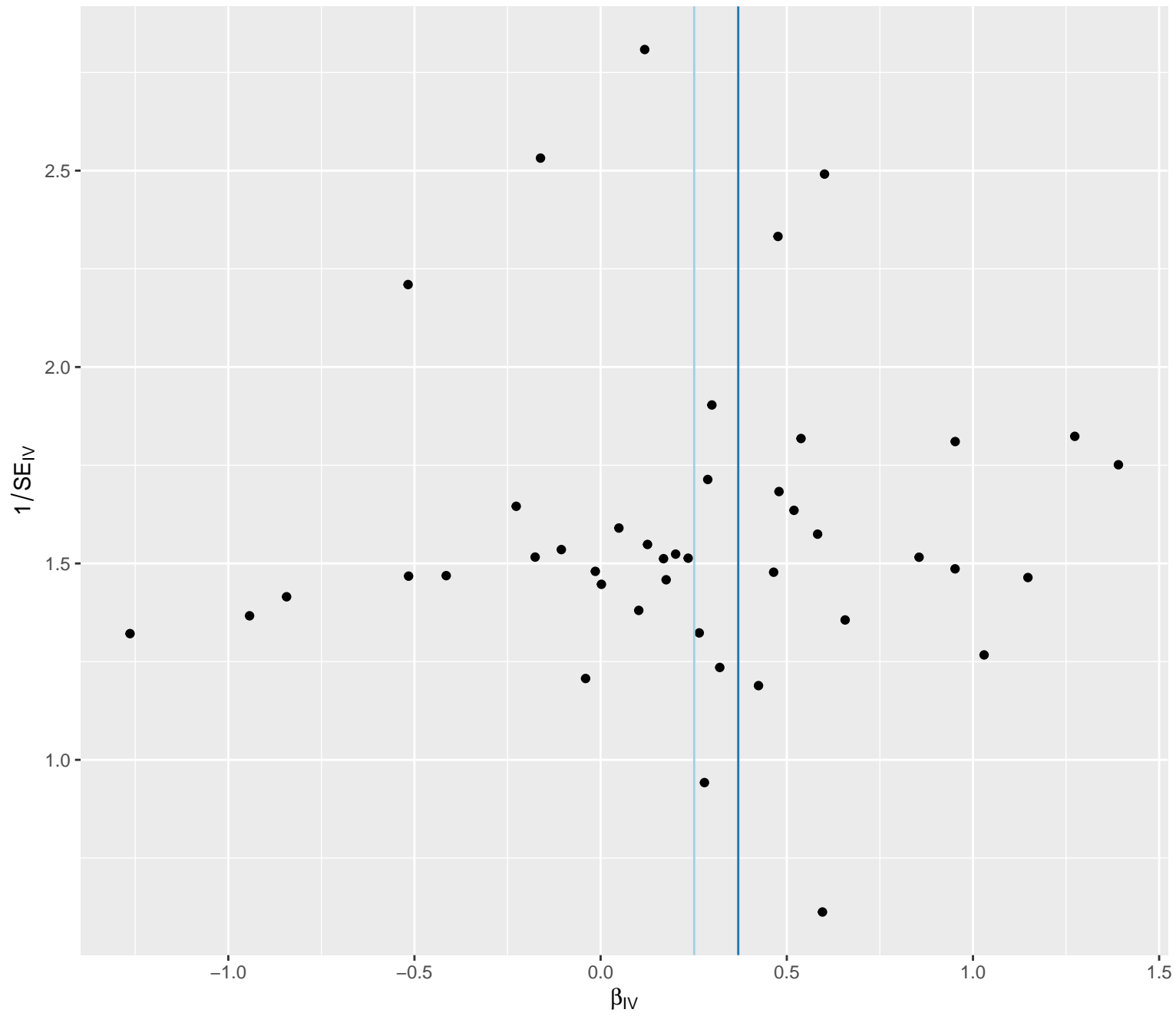

Supplement: Supplementary file 8 — Data S4: Supporting Information. [file ADB-31-e70160-s001.zip › Additional file4/Forward MR analysis/GCST90002780.pdf]

# MR Method

- Inverse variance weighted
- MR Egger

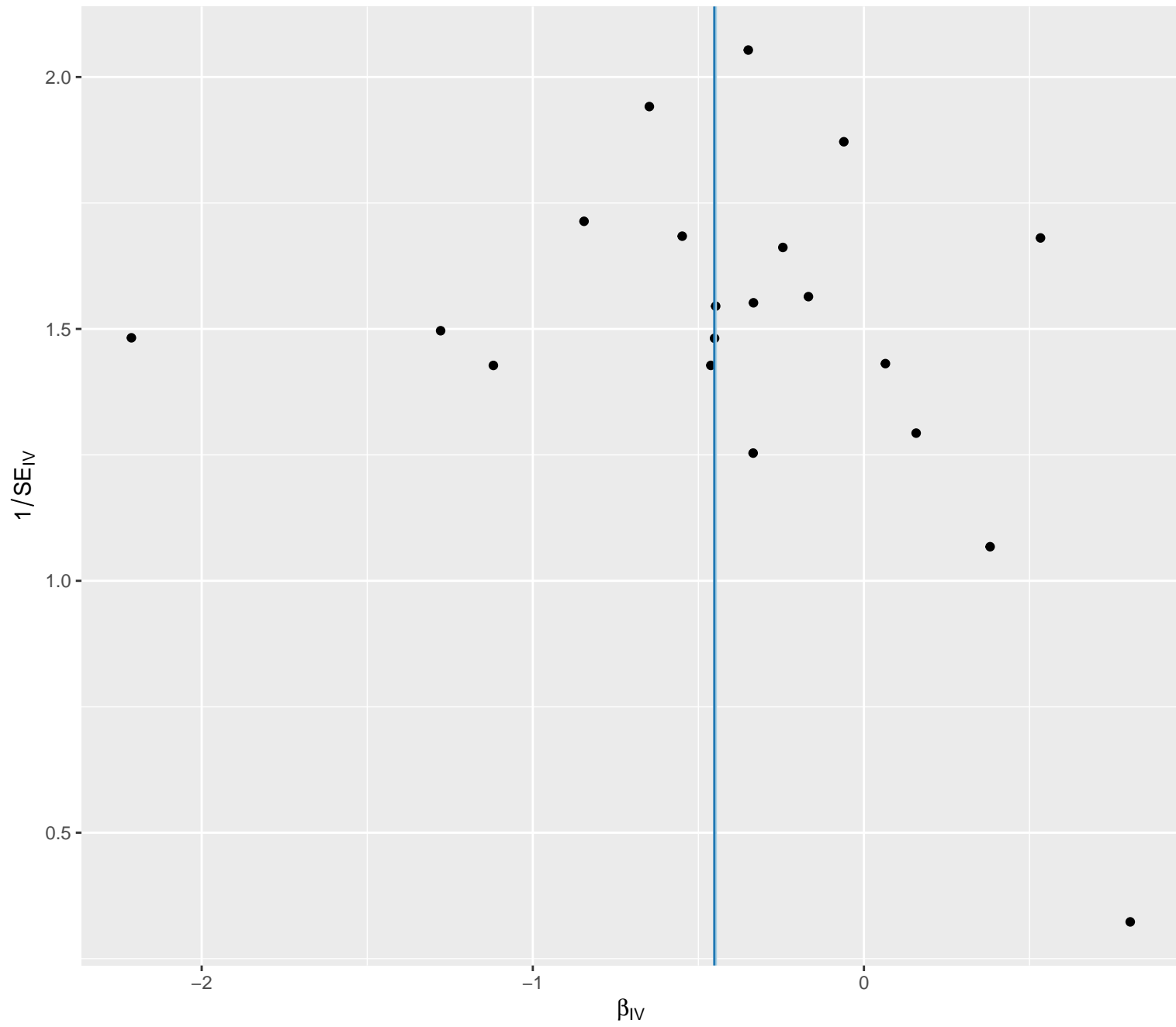

Supplement: Supplementary file 8 — Data S4: Supporting Information. [file ADB-31-e70160-s001.zip › Additional file4/Forward MR analysis/GCST90005372.pdf]

# MR Method

- Inverse variance weighted
- MR Egger

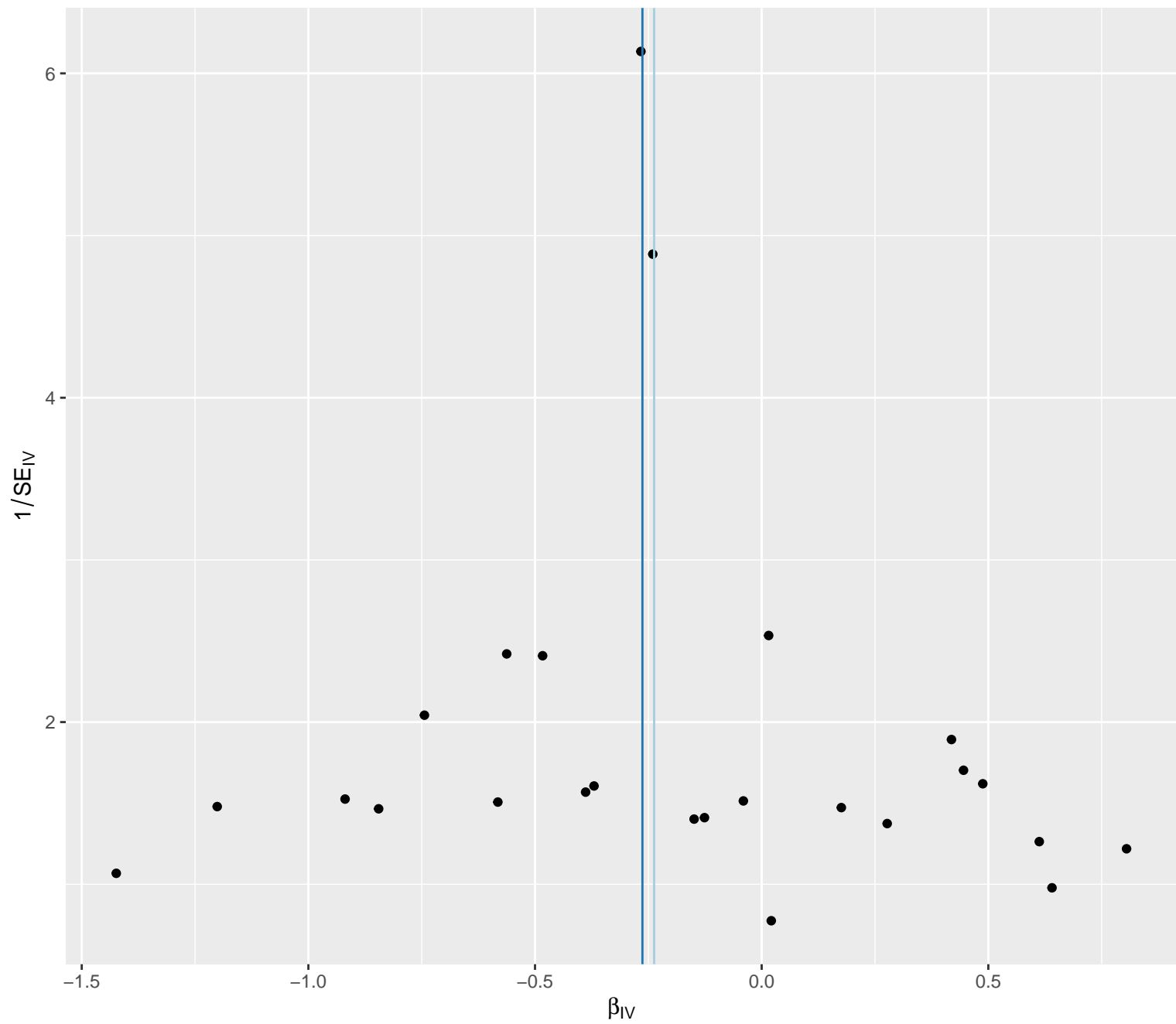

Supplement: Supplementary file 8 — Data S4: Supporting Information. [file ADB-31-e70160-s001.zip › Additional file4/Forward MR analysis/GCST90004898.pdf]

# MR Method

- Inverse variance weighted
- MR Egger

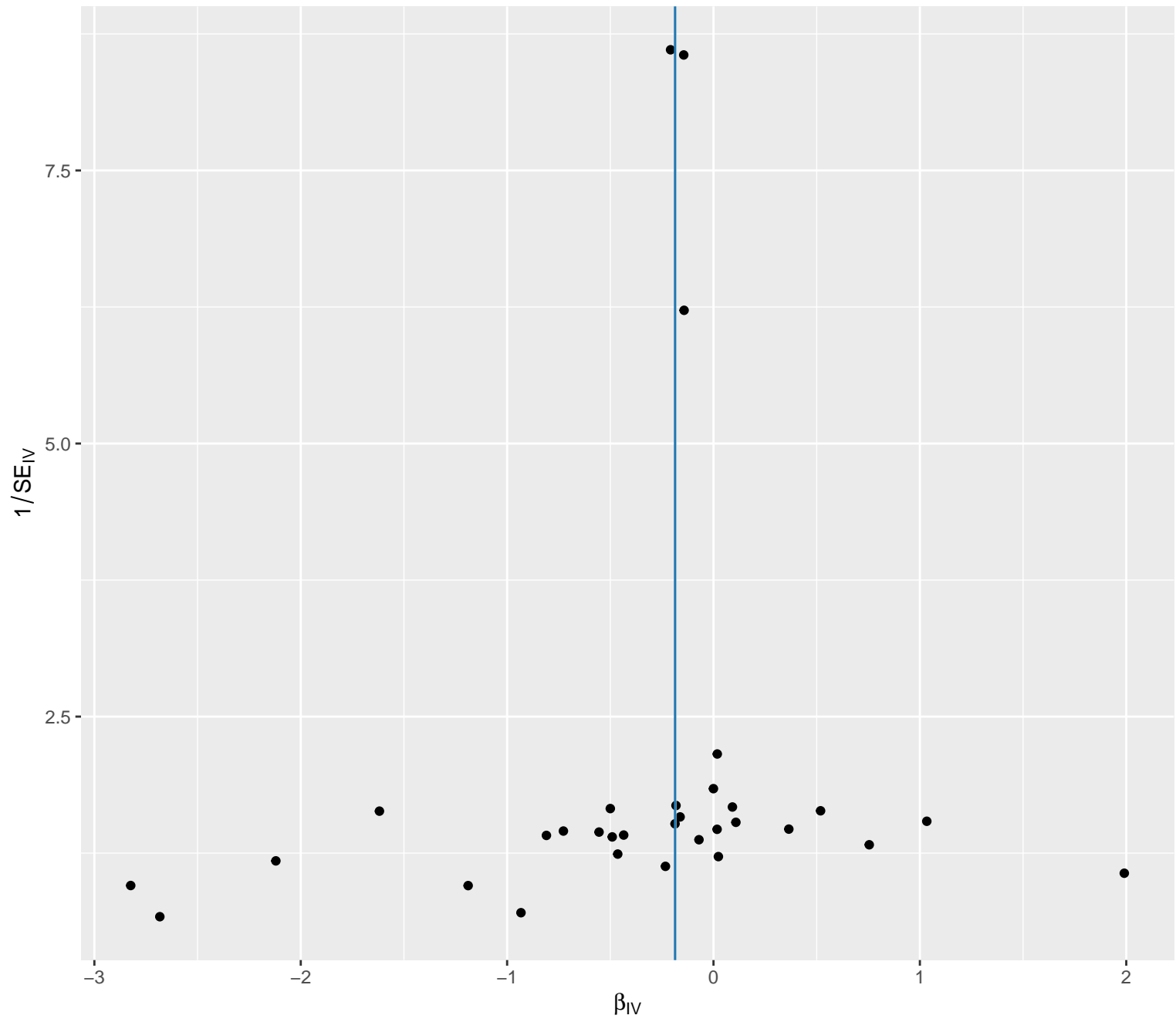

Supplement: Supplementary file 8 — Data S4: Supporting Information. [file ADB-31-e70160-s001.zip › Additional file4/Forward MR analysis/GCST90002542.pdf]

# MR Method

- Inverse variance weighted
- MR Egger

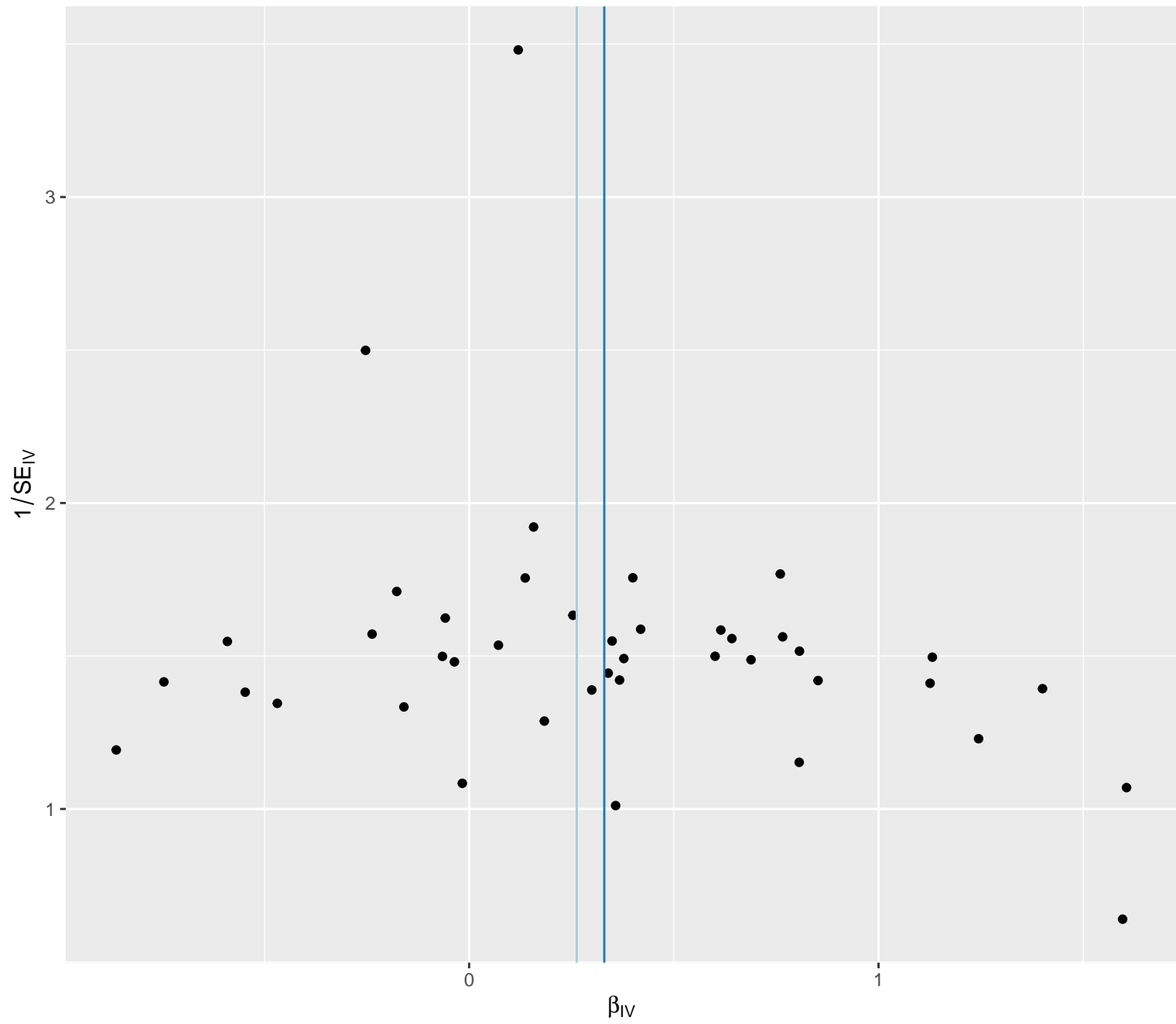

Supplement: Supplementary file 8 — Data S4: Supporting Information. [file ADB-31-e70160-s001.zip › Additional file4/Forward MR analysis/GCST90003714.pdf]

# MR Method

- Inverse variance weighted
- MR Egger

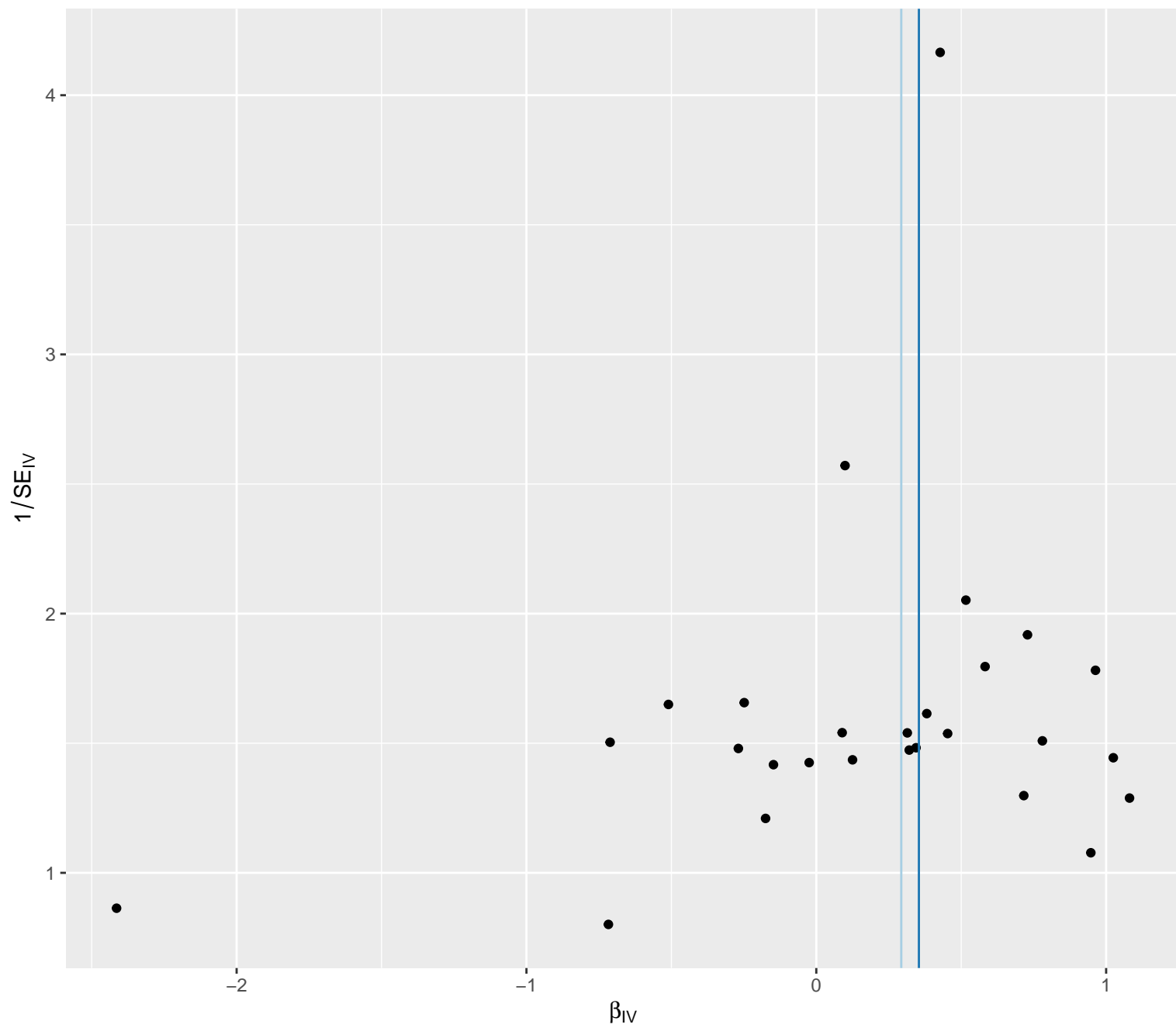

Supplement: Supplementary file 8 — Data S4: Supporting Information. [file ADB-31-e70160-s001.zip › Additional file4/Forward MR analysis/GCST90005629.pdf]

# MR Method

- Inverse variance weighted
- MR Egger

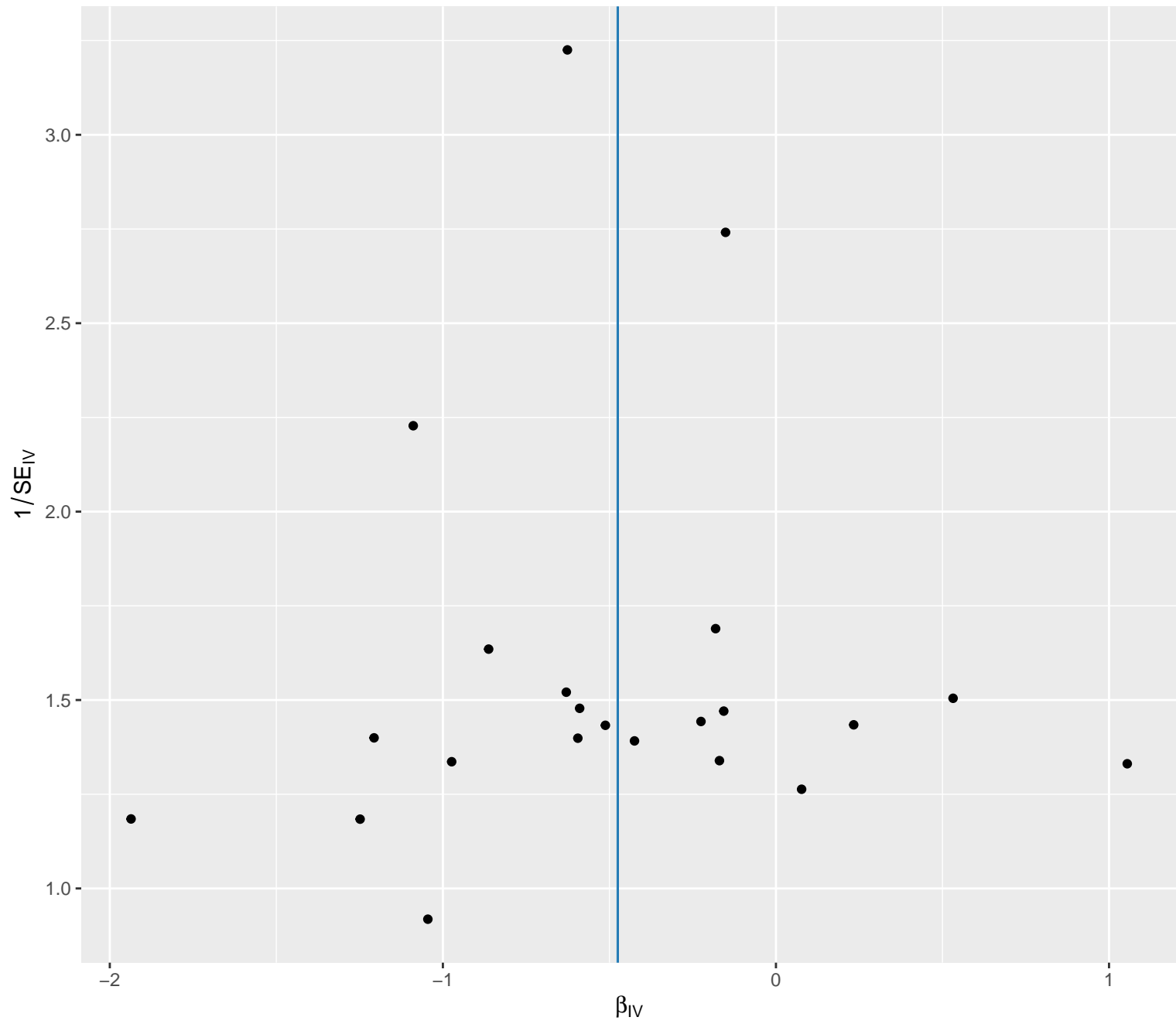

Supplement: Supplementary file 8 — Data S4: Supporting Information. [file ADB-31-e70160-s001.zip › Additional file4/Forward MR analysis/GCST90006308.pdf]

# MR Method

- Inverse variance weighted
- MR Egger

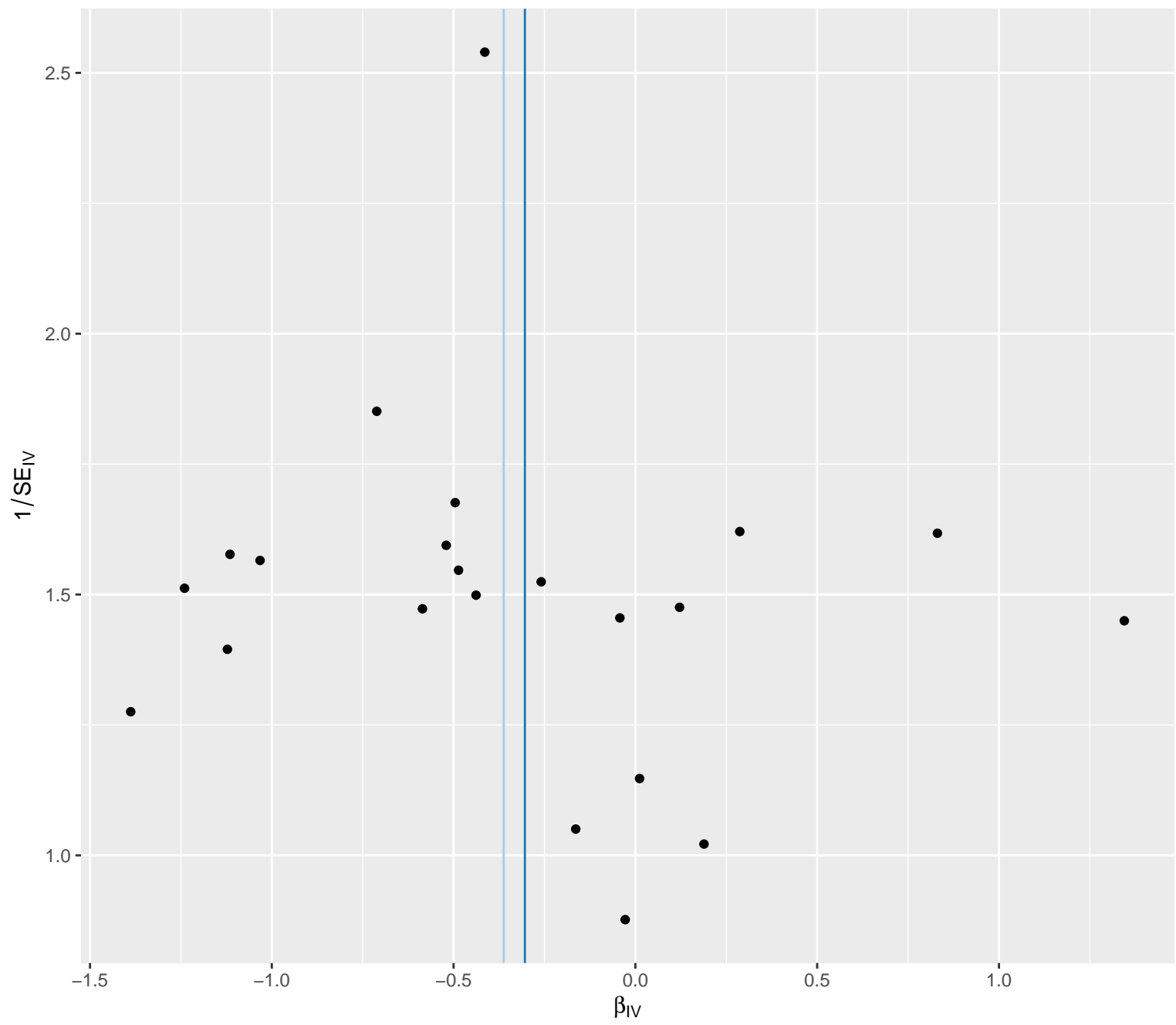

Supplement: Supplementary file 8 — Data S4: Supporting Information. [file ADB-31-e70160-s001.zip › Additional file4/Forward MR analysis/GCST90004578.pdf]

# MR Method

- Inverse variance weighted
- MR Egger

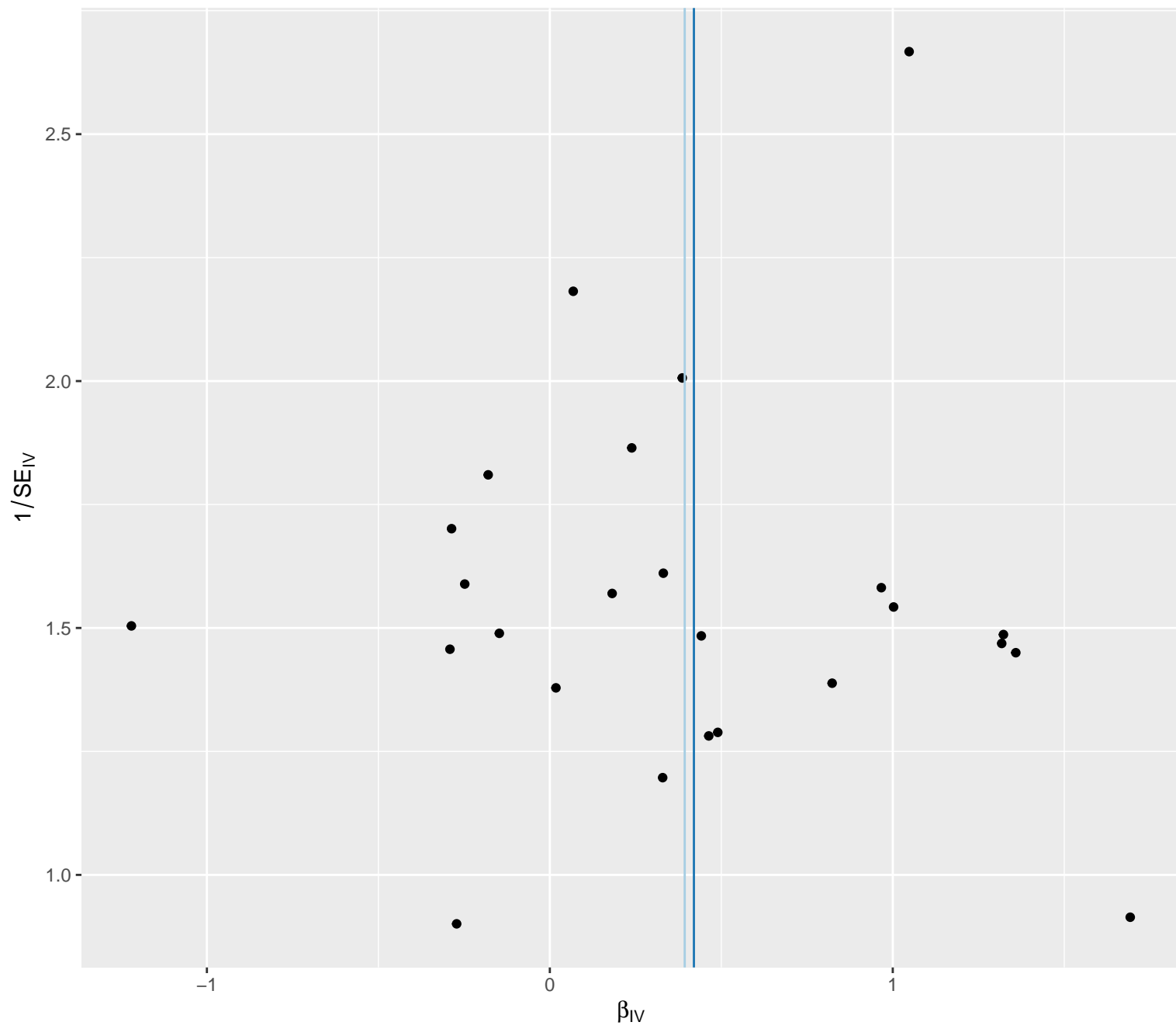

Supplement: Supplementary file 8 — Data S4: Supporting Information. [file ADB-31-e70160-s001.zip › Additional file4/Forward MR analysis/GCST90005248.pdf]

# MR Method

- Inverse variance weighted
- MR Egger

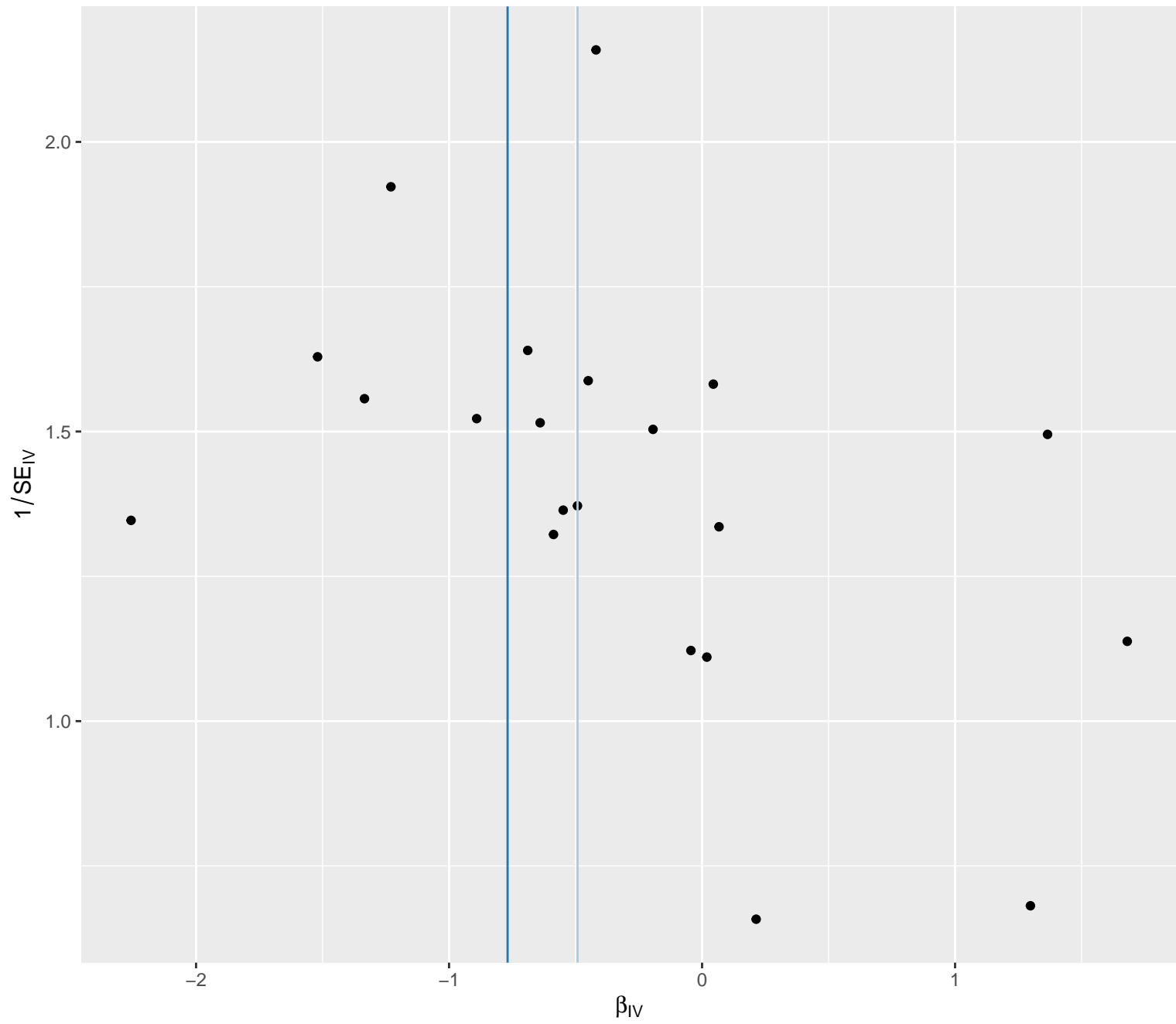

Supplement: Supplementary file 8 — Data S4: Supporting Information. [file ADB-31-e70160-s001.zip › Additional file4/Forward MR analysis/GCST90005116.pdf]

# MR Method

- Inverse variance weighted
- MR Egger

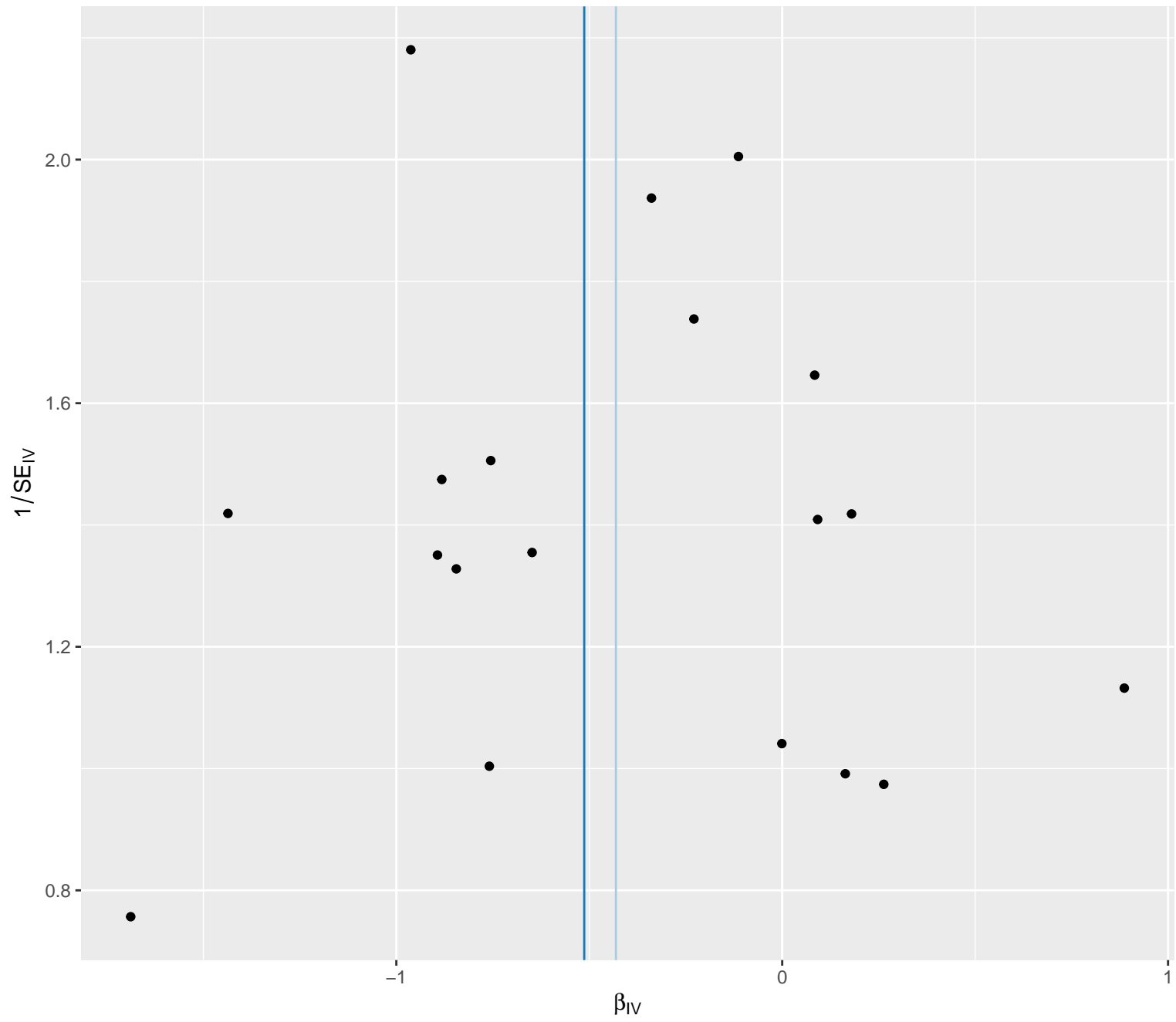

Supplement: Supplementary file 8 — Data S4: Supporting Information. [file ADB-31-e70160-s001.zip › Additional file4/Forward MR analysis/GCST90005660.pdf]

# MR Method

- Inverse variance weighted
- MR Egger

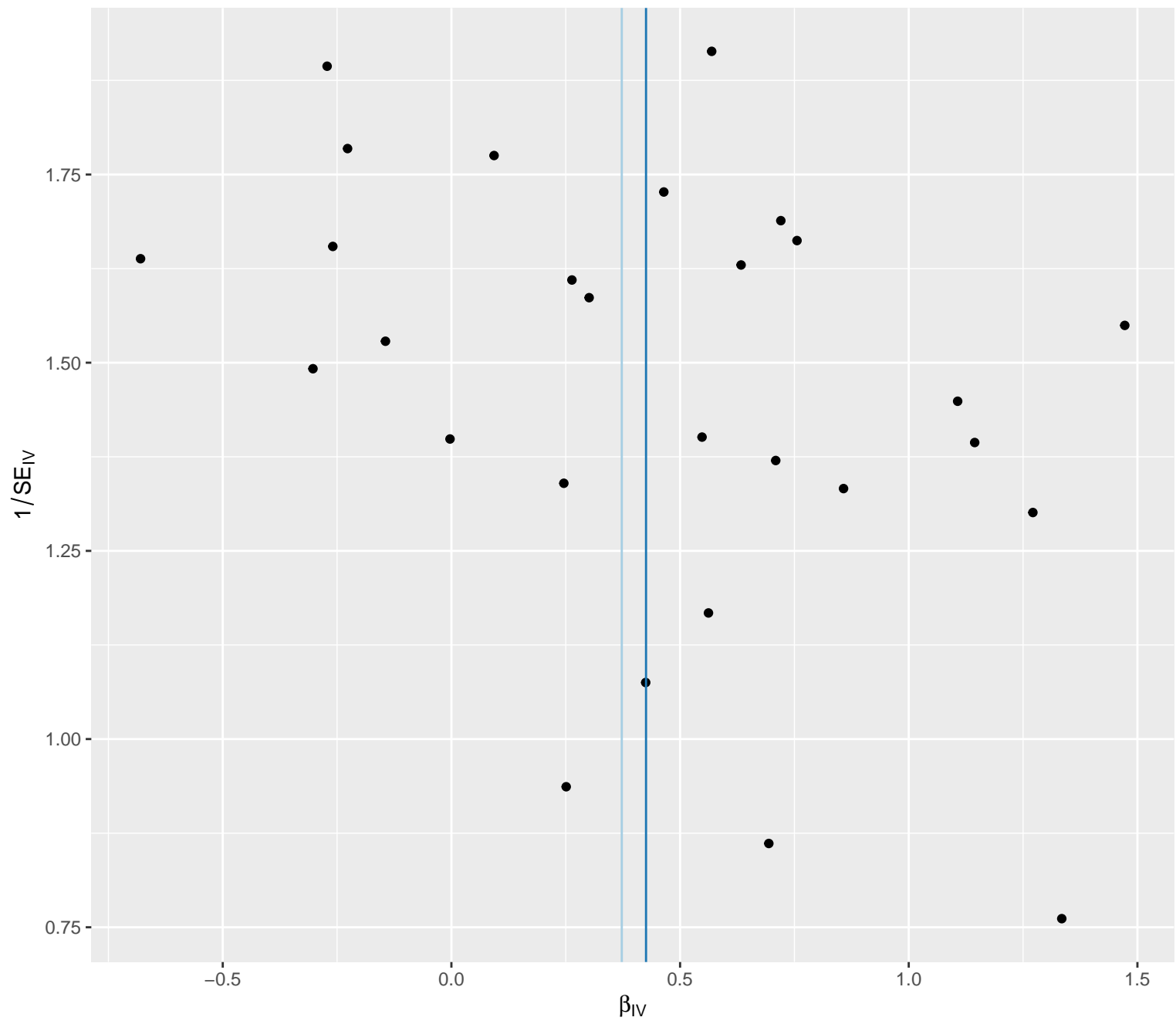

Supplement: Supplementary file 8 — Data S4: Supporting Information. [file ADB-31-e70160-s001.zip › Additional file4/Forward MR analysis/GCST90005715.pdf]

# MR Method

- Inverse variance weighted
- MR Egger

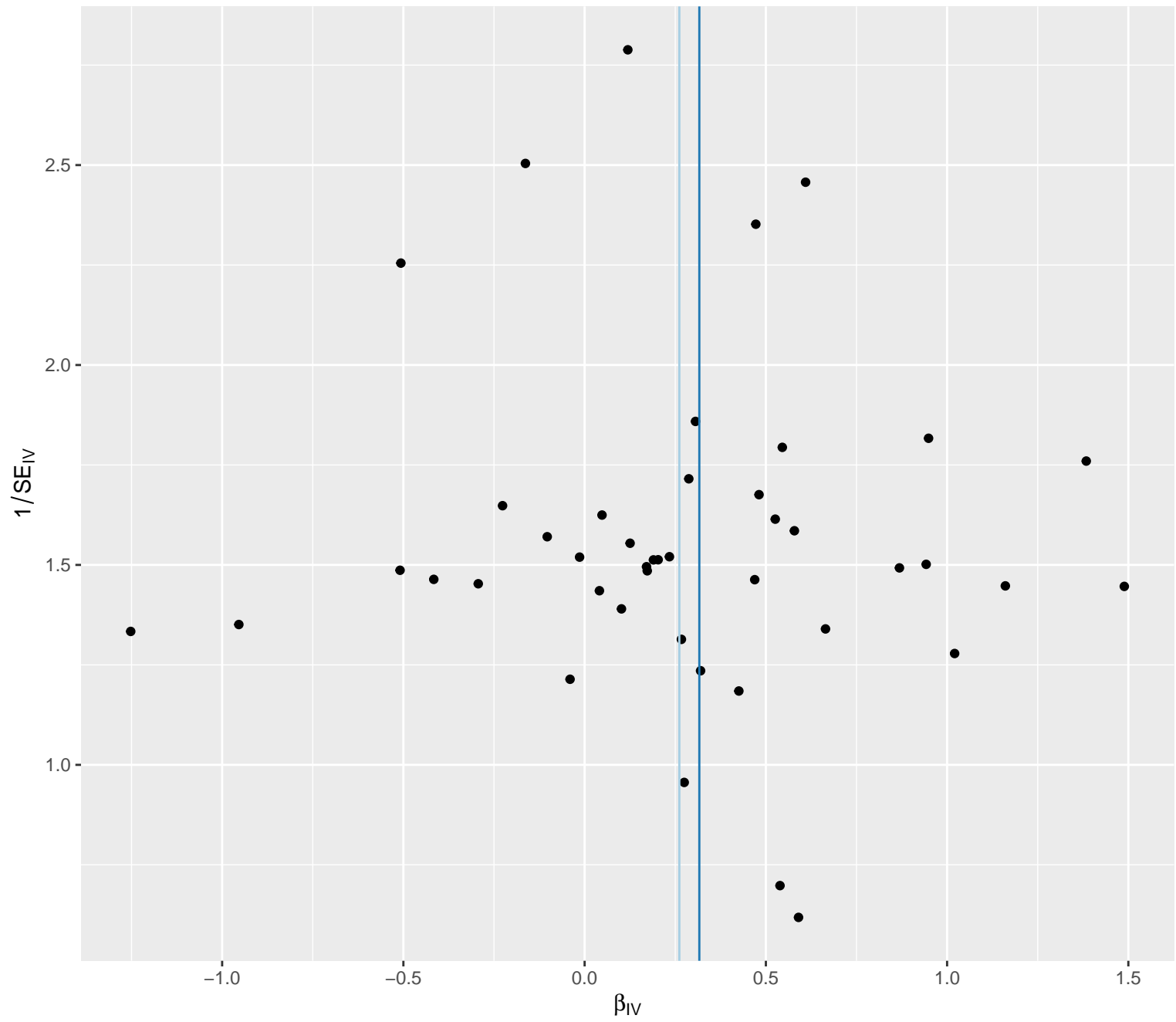

Supplement: Supplementary file 8 — Data S4: Supporting Information. [file ADB-31-e70160-s001.zip › Additional file4/Forward MR analysis/GCST90002873.pdf]

# MR Method

- Inverse variance weighted
- MR Egger

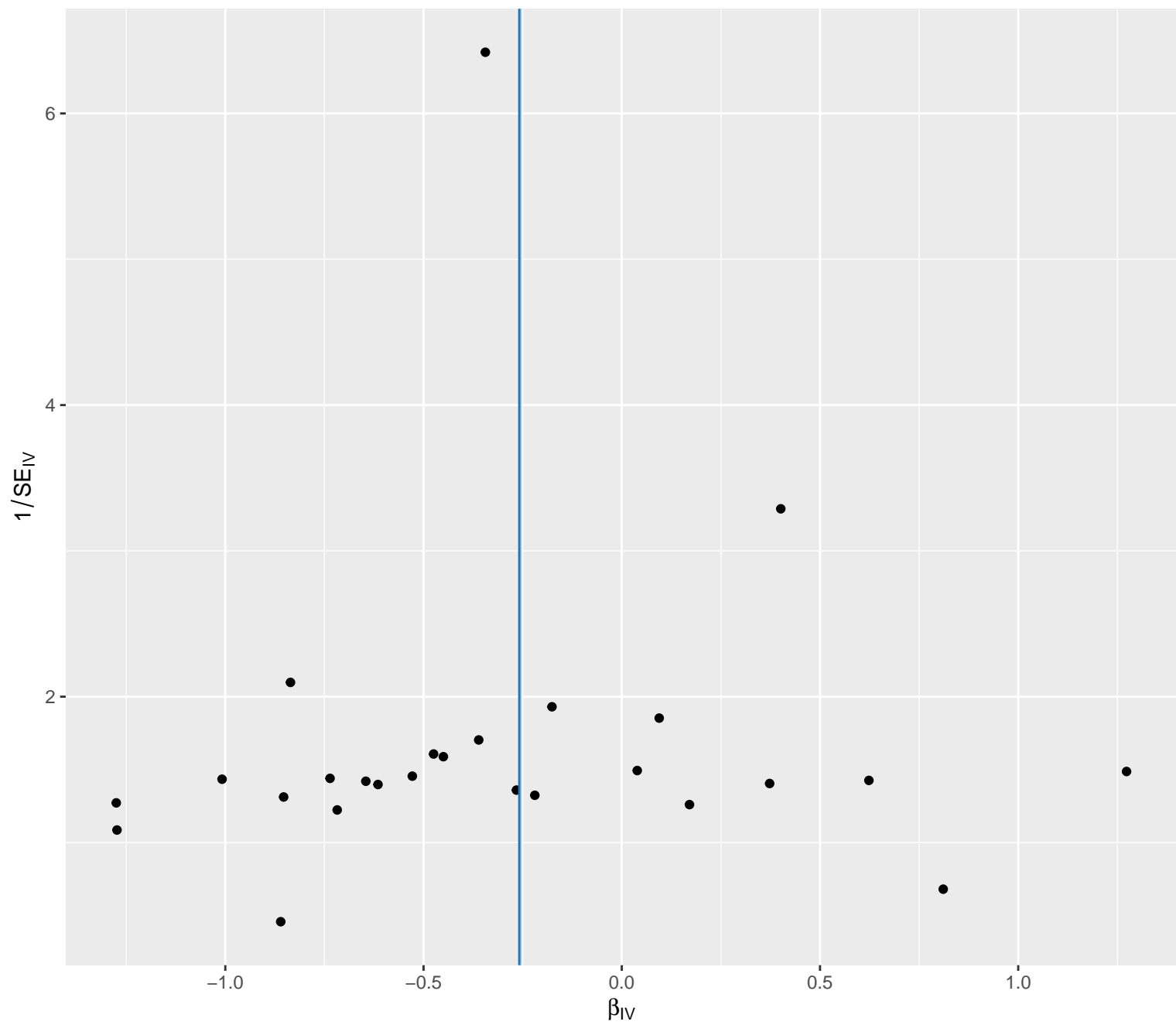

Supplement: Supplementary file 8 — Data S4: Supporting Information. [file ADB-31-e70160-s001.zip › Additional file4/Forward MR analysis/GCST90005844.pdf]

# MR Method

- Inverse variance weighted
- MR Egger

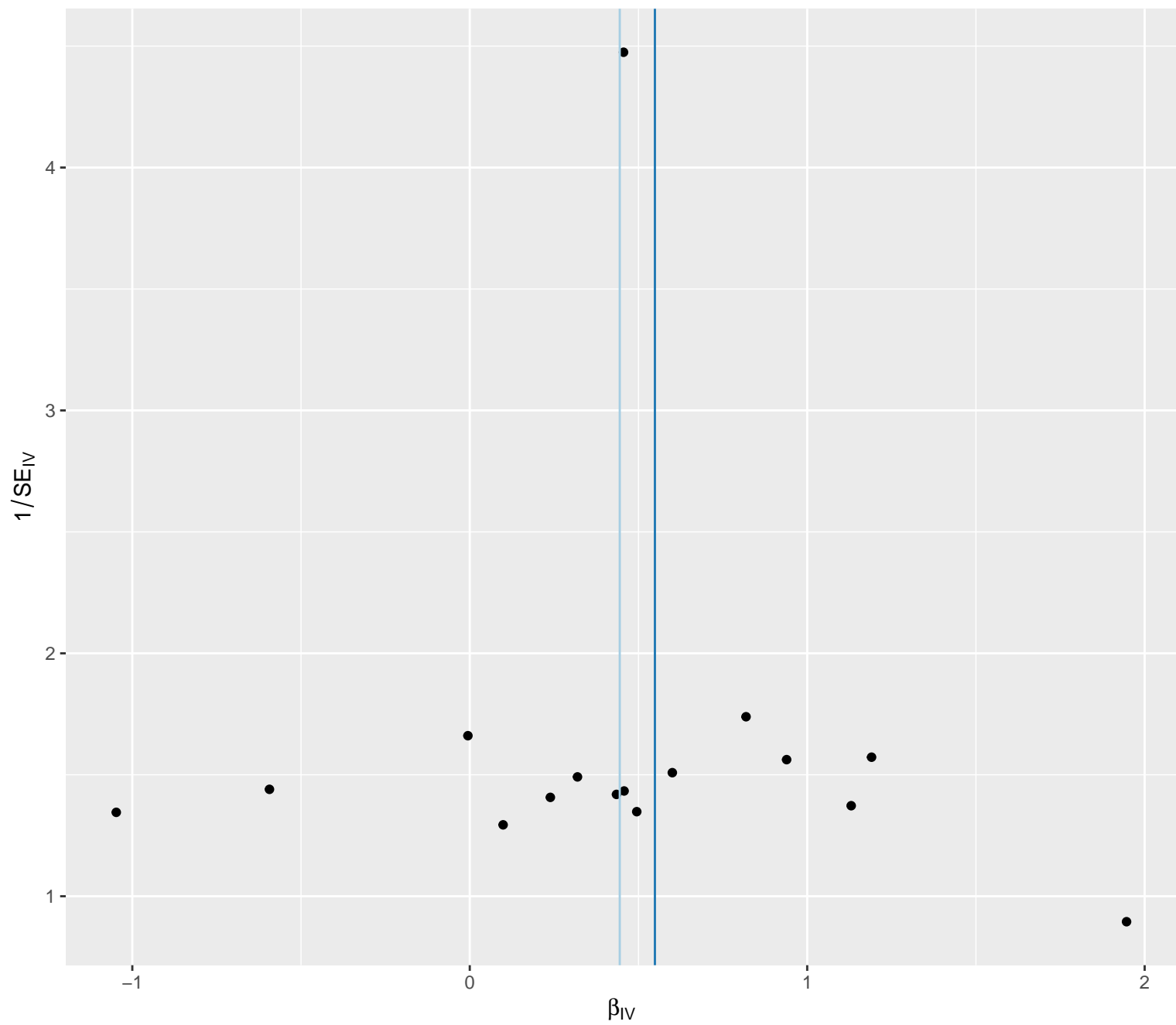

Supplement: Supplementary file 8 — Data S4: Supporting Information. [file ADB-31-e70160-s001.zip › Additional file4/Forward MR analysis/GCST90005108.pdf]

# MR Method

- Inverse variance weighted
- MR Egger

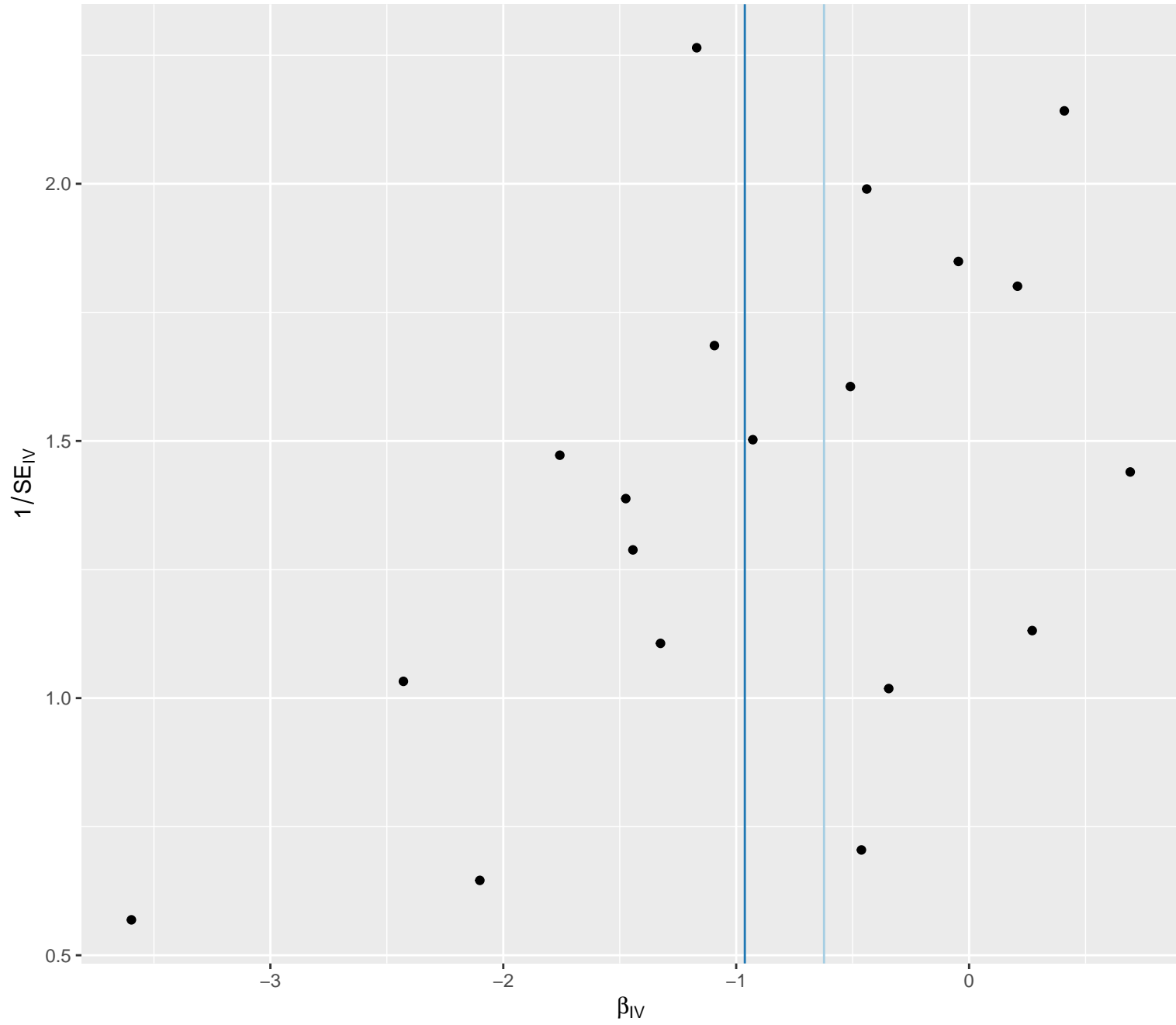

Supplement: Supplementary file 8 — Data S4: Supporting Information. [file ADB-31-e70160-s001.zip › Additional file4/Forward MR analysis/GCST90005687.pdf]

# MR Method

- Inverse variance weighted
- MR Egger

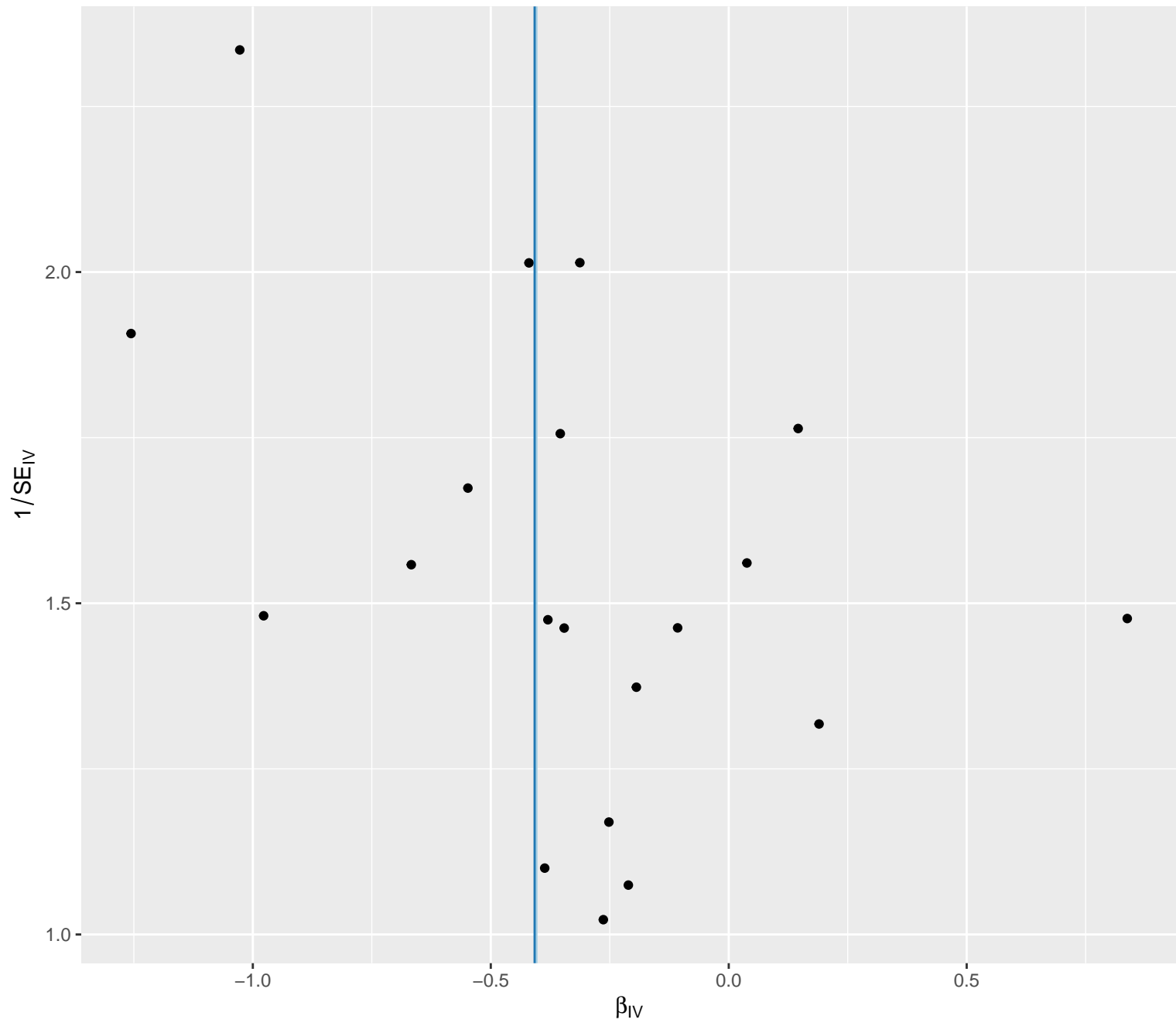

Supplement: Supplementary file 8 — Data S4: Supporting Information. [file ADB-31-e70160-s001.zip › Additional file4/Forward MR analysis/GCST90006239.pdf]

# MR Method

- Inverse variance weighted
- MR Egger

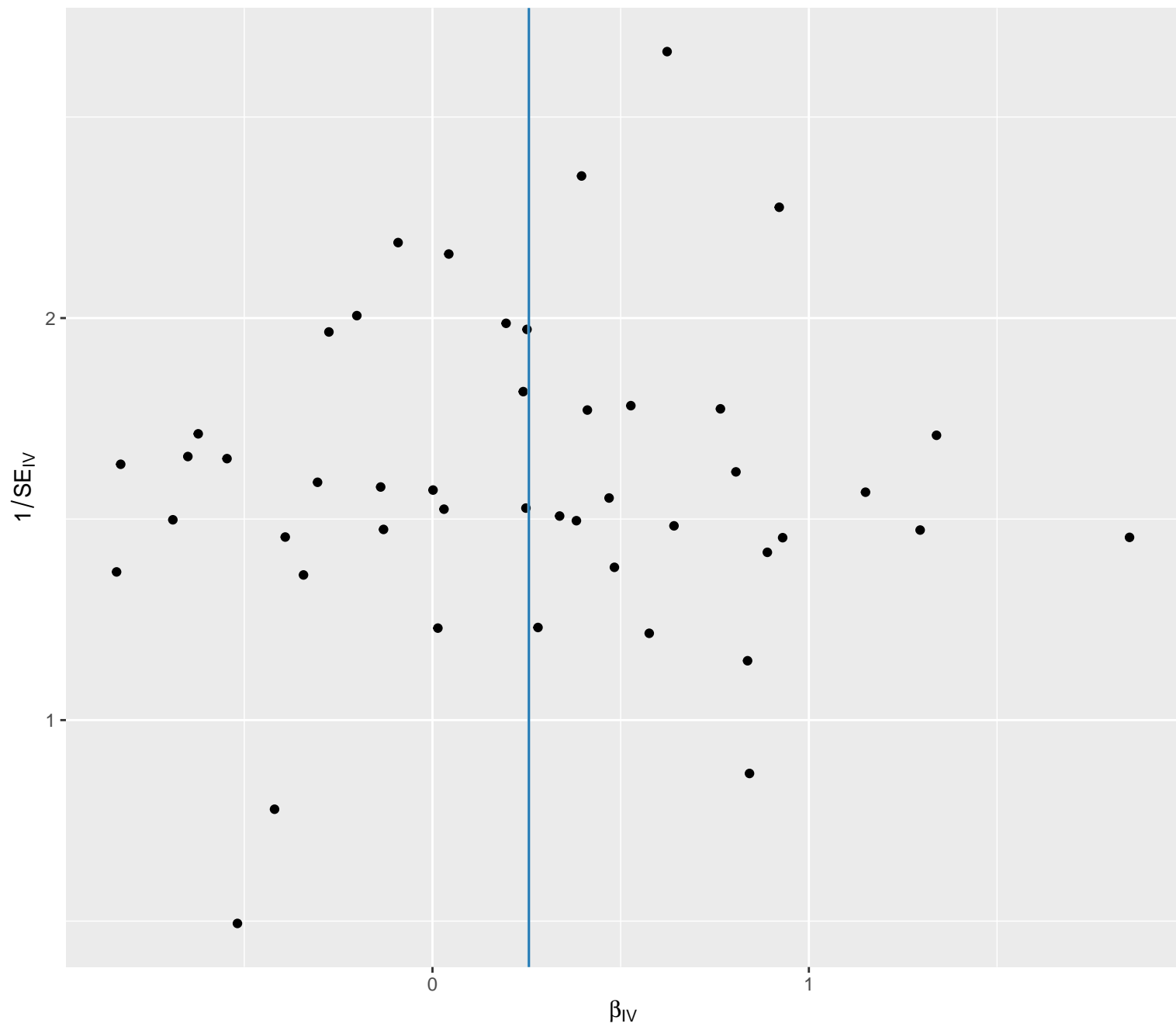

Supplement: Supplementary file 8 — Data S4: Supporting Information. [file ADB-31-e70160-s001.zip › Additional file4/Forward MR analysis/GCST90003807.pdf]

# MR Method

- Inverse variance weighted
- MR Egger

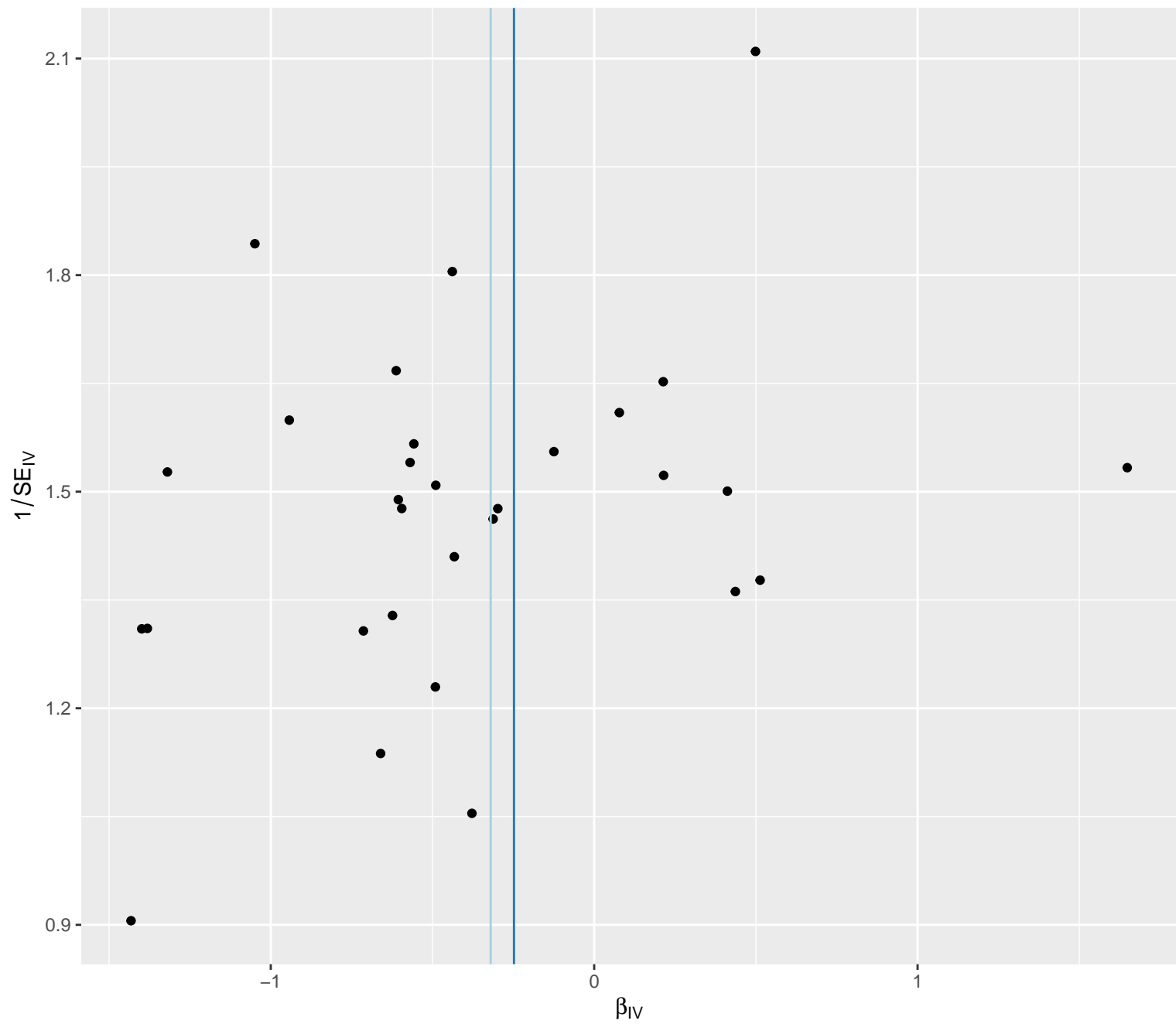

Supplement: Supplementary file 8 — Data S4: Supporting Information. [file ADB-31-e70160-s001.zip › Additional file4/Forward MR analysis/GCST90004004.pdf]

# MR Method

- Inverse variance weighted
- MR Egger

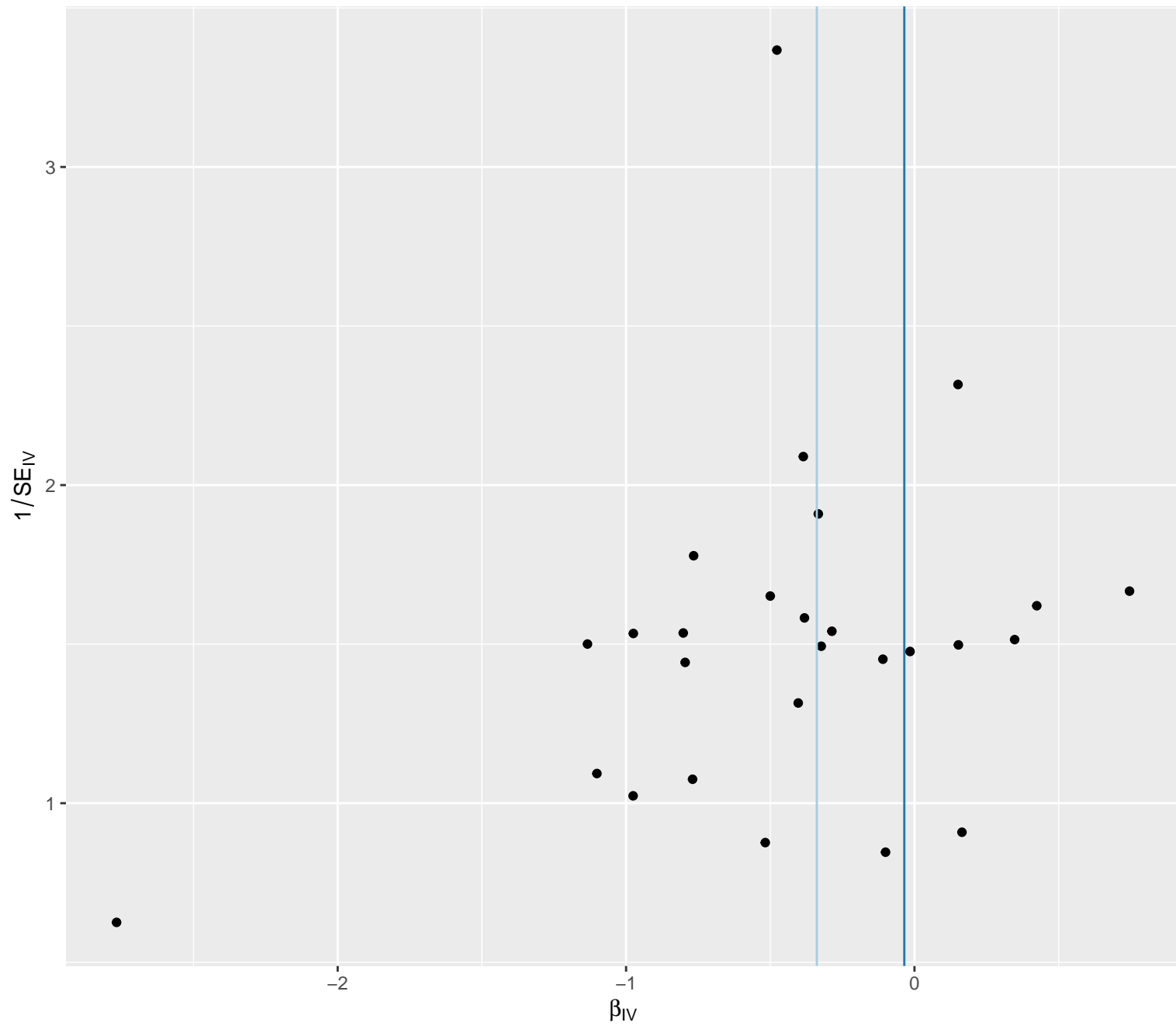

Supplement: Supplementary file 8 — Data S4: Supporting Information. [file ADB-31-e70160-s001.zip › Additional file4/Forward MR analysis/GCST90004301.pdf]

# MR Method

- Inverse variance weighted
- MR Egger

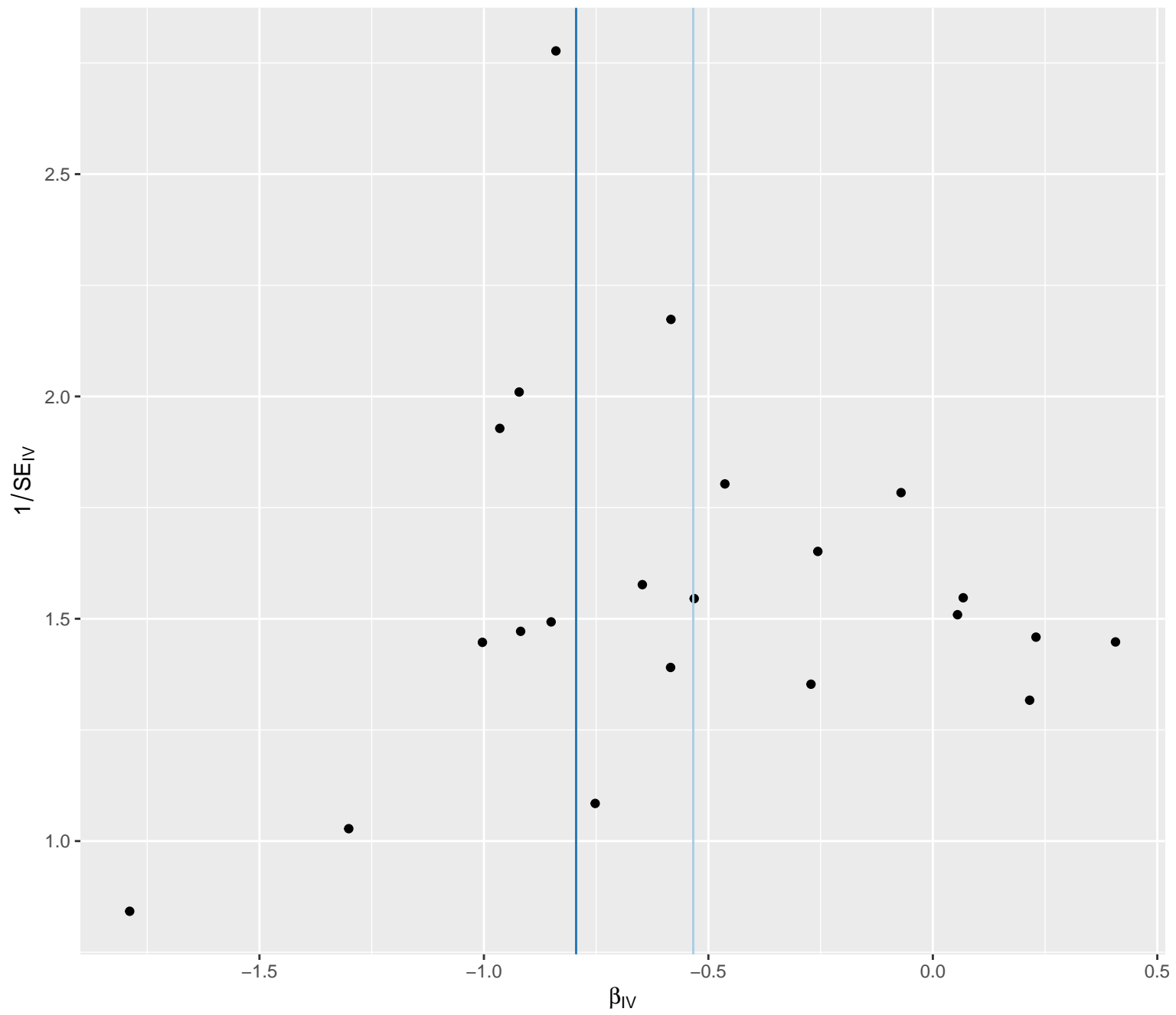

Supplement: Supplementary file 8 — Data S4: Supporting Information. [file ADB-31-e70160-s001.zip › Additional file4/Forward MR analysis/GCST90005182.pdf]

# MR Method

- Inverse variance weighted
- MR Egger

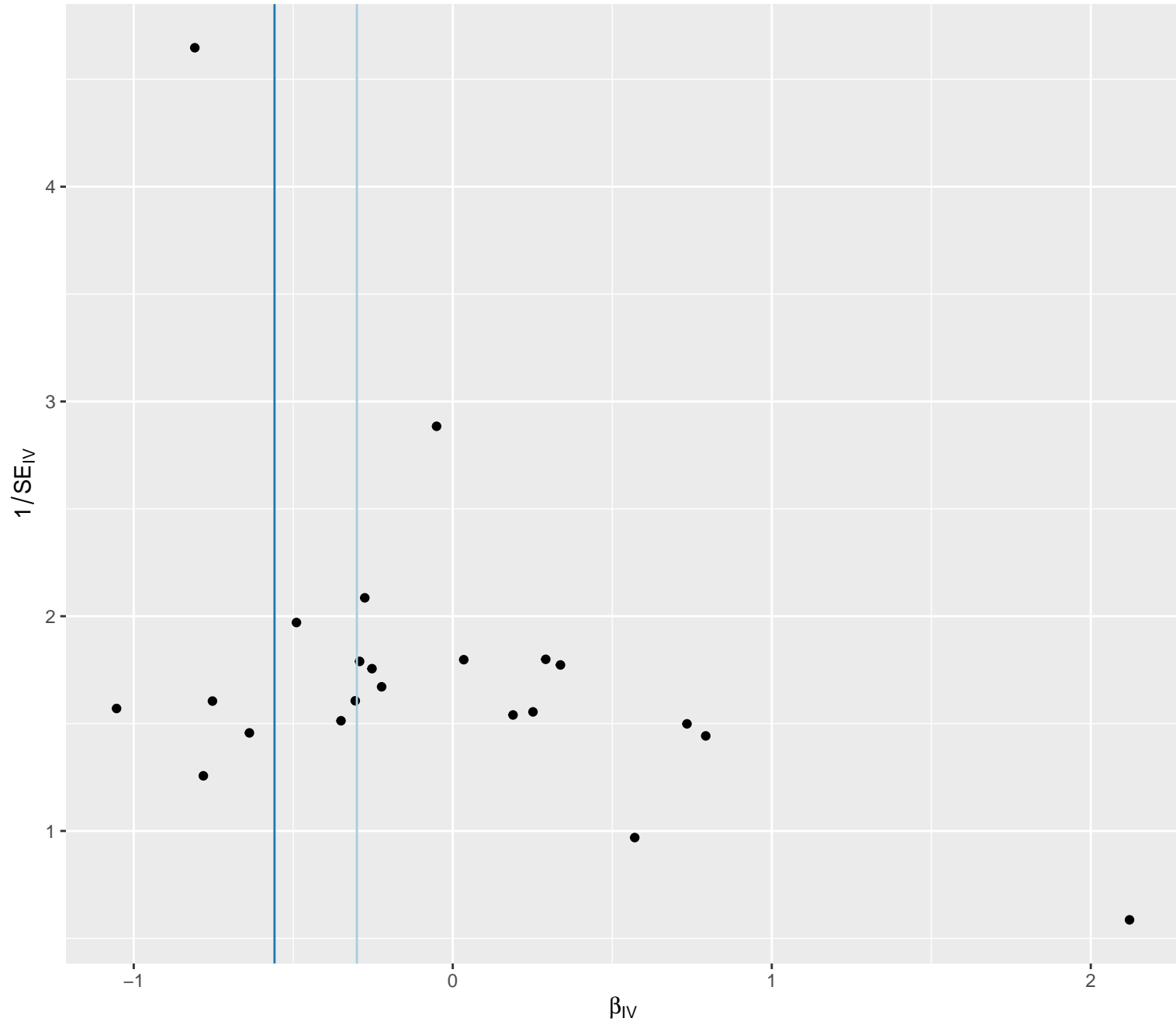

Supplement: Supplementary file 8 — Data S4: Supporting Information. [file ADB-31-e70160-s001.zip › Additional file4/Forward MR analysis/GCST90006306.pdf]

# MR Method

- Inverse variance weighted
- MR Egger

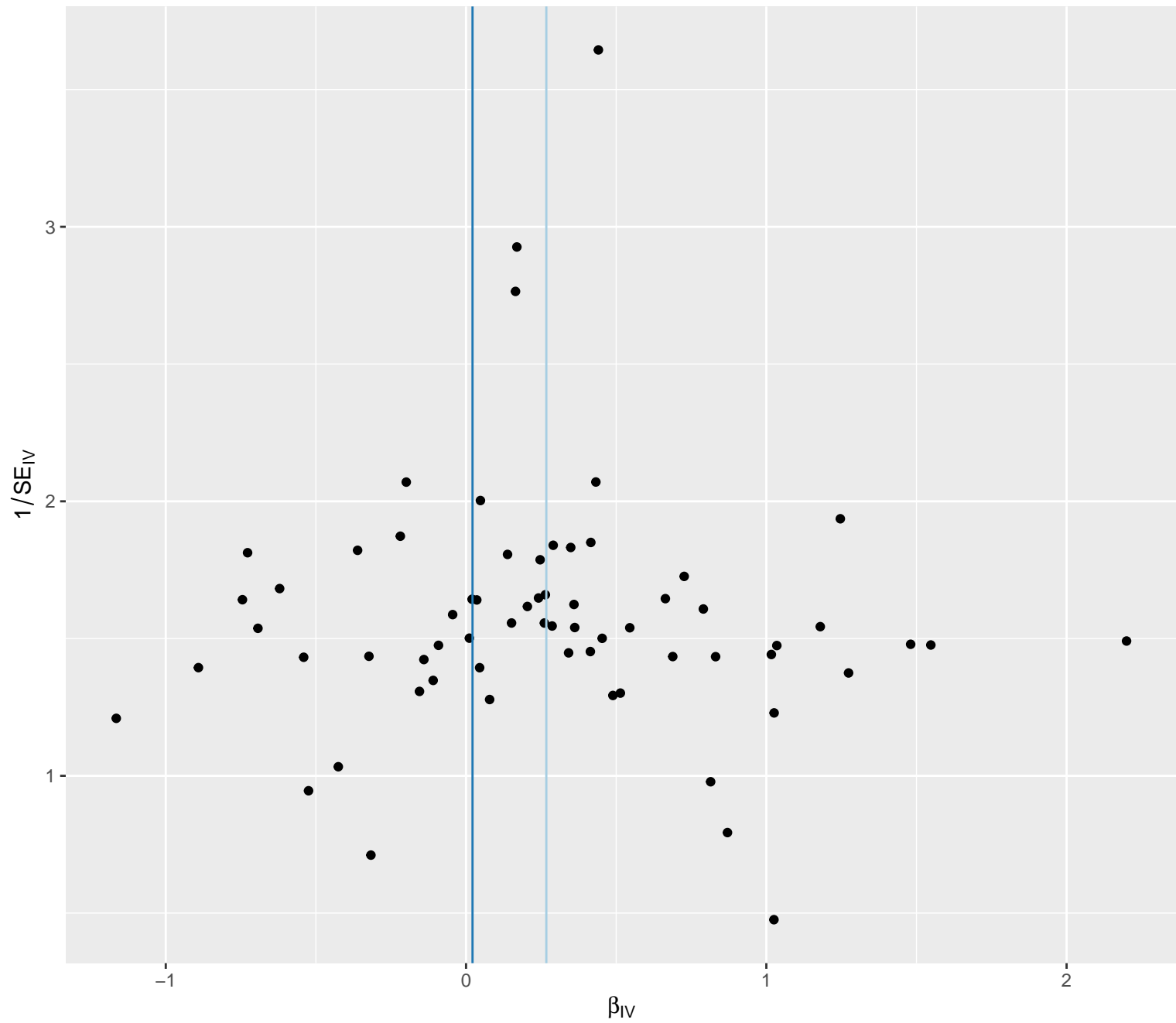

Supplement: Supplementary file 8 — Data S4: Supporting Information. [file ADB-31-e70160-s001.zip › Additional file4/Forward MR analysis/GCST90003861.pdf]

# MR Method

- Inverse variance weighted
- MR Egger

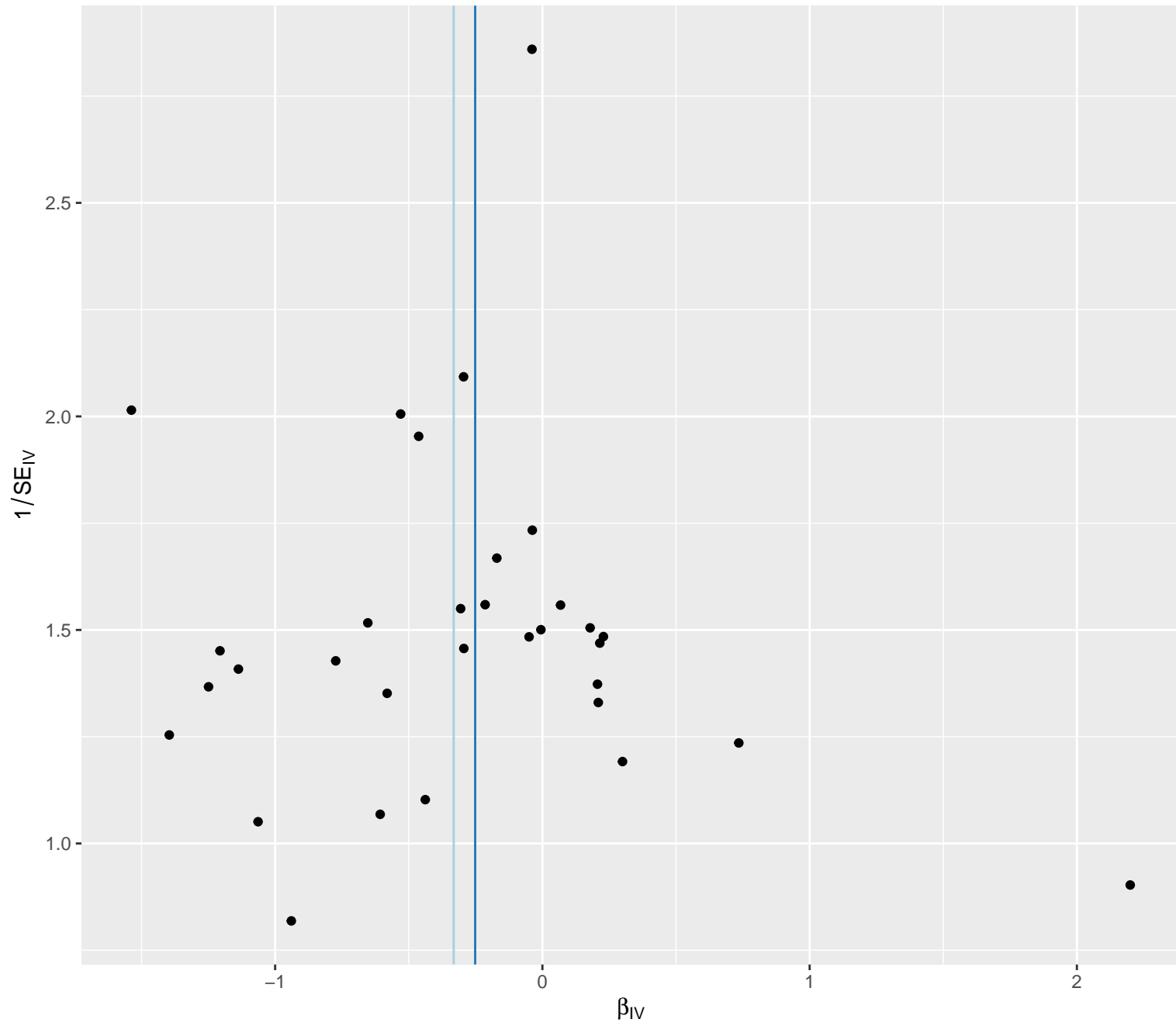

Supplement: Supplementary file 8 — Data S4: Supporting Information. [file ADB-31-e70160-s001.zip › Additional file4/Forward MR analysis/GCST90005975.pdf]

# MR Method

- Inverse variance weighted
- MR Egger

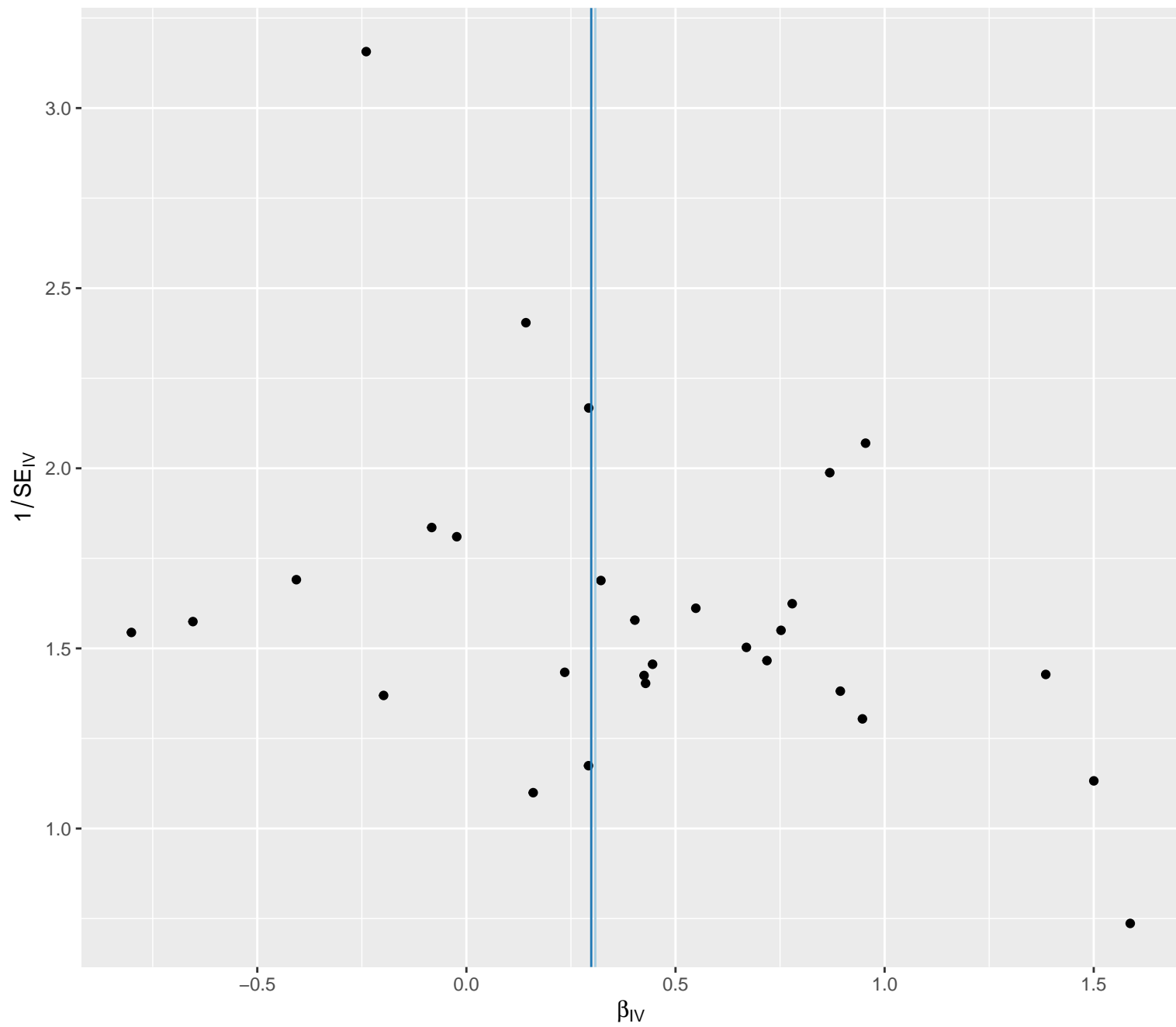

Supplement: Supplementary file 8 — Data S4: Supporting Information. [file ADB-31-e70160-s001.zip › Additional file4/Forward MR analysis/GCST90003733.pdf]

# MR Method

- Inverse variance weighted
- MR Egger

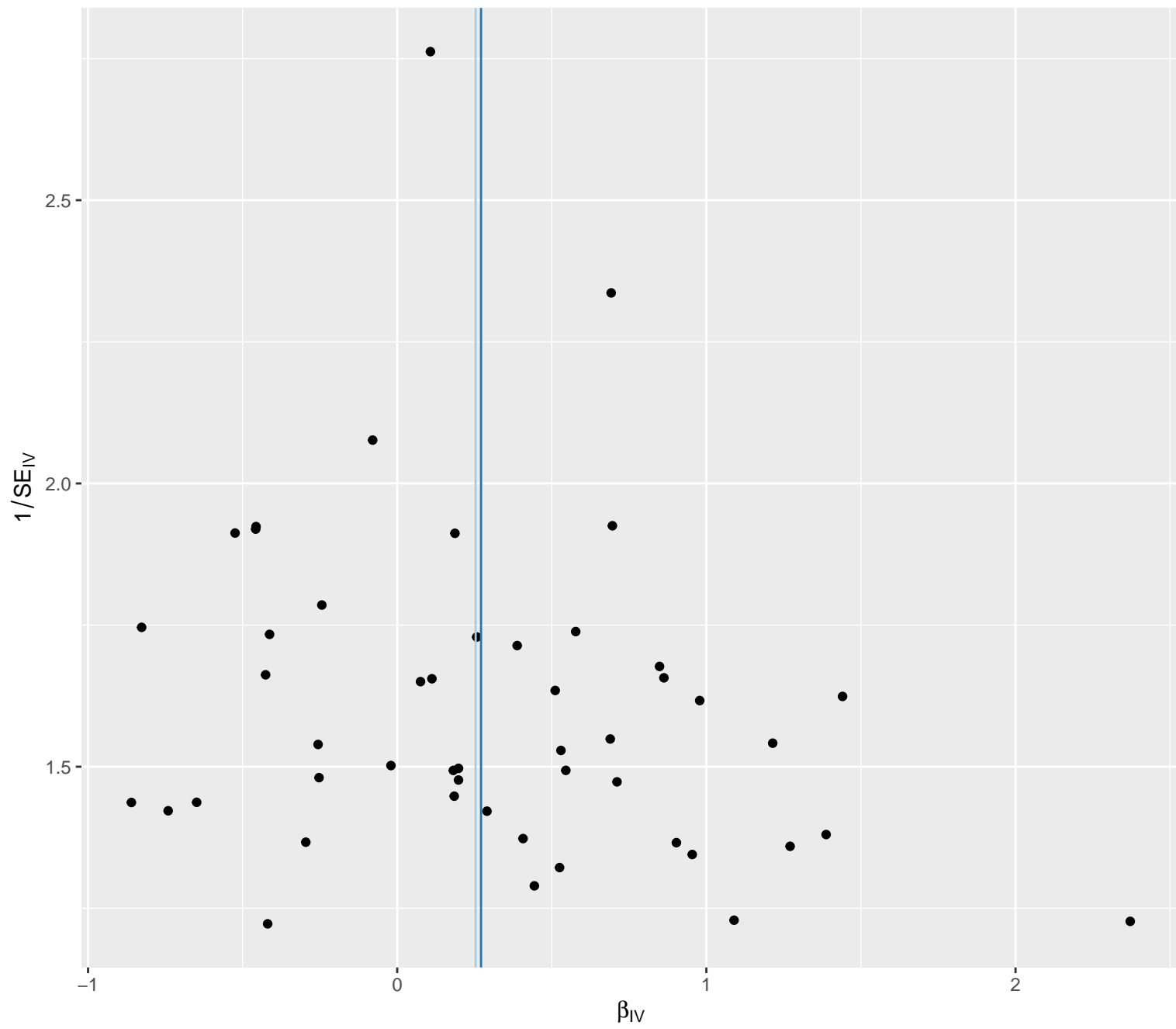

Supplement: Supplementary file 8 — Data S4: Supporting Information. [file ADB-31-e70160-s001.zip › Additional file4/Forward MR analysis/GCST90003131.pdf]

# MR Method

- Inverse variance weighted
- MR Egger

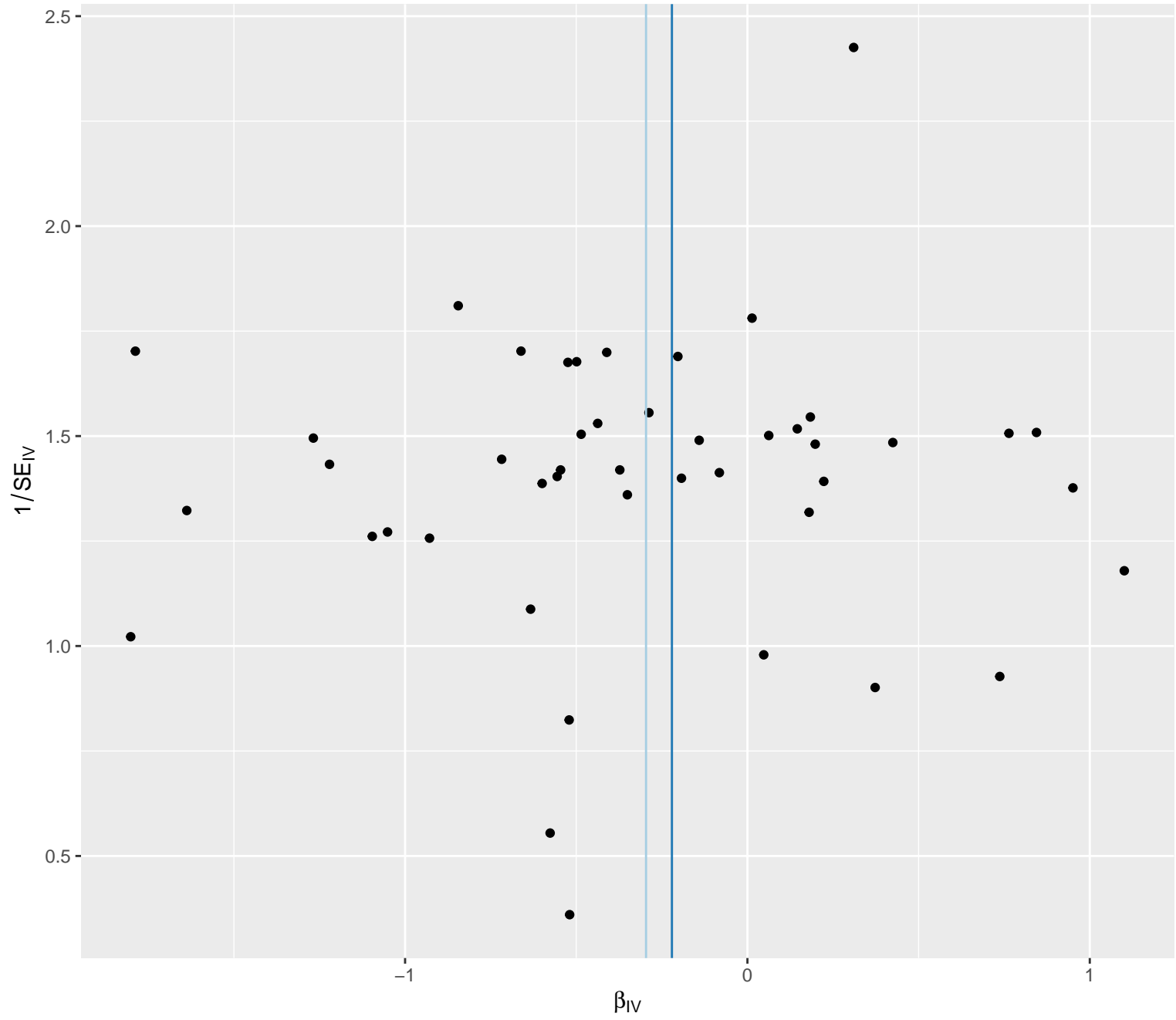

Supplement: Supplementary file 8 — Data S4: Supporting Information. [file ADB-31-e70160-s001.zip › Additional file4/Forward MR analysis/GCST90002561.pdf]

# MR Method

- Inverse variance weighted
- MR Egger

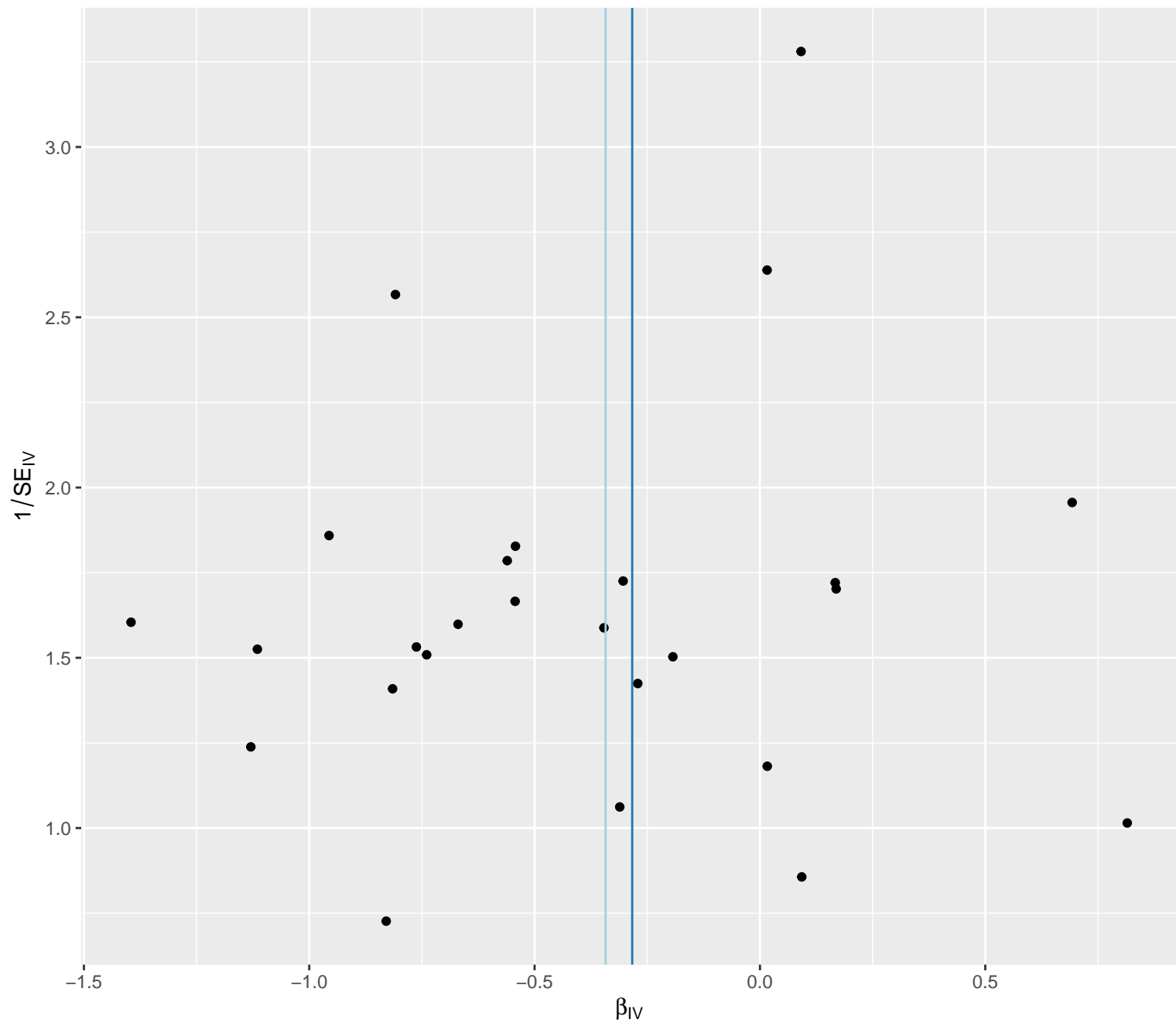

Supplement: Supplementary file 8 — Data S4: Supporting Information. [file ADB-31-e70160-s001.zip › Additional file4/Forward MR analysis/GCST90005971.pdf]

# MR Method

- Inverse variance weighted
- MR Egger

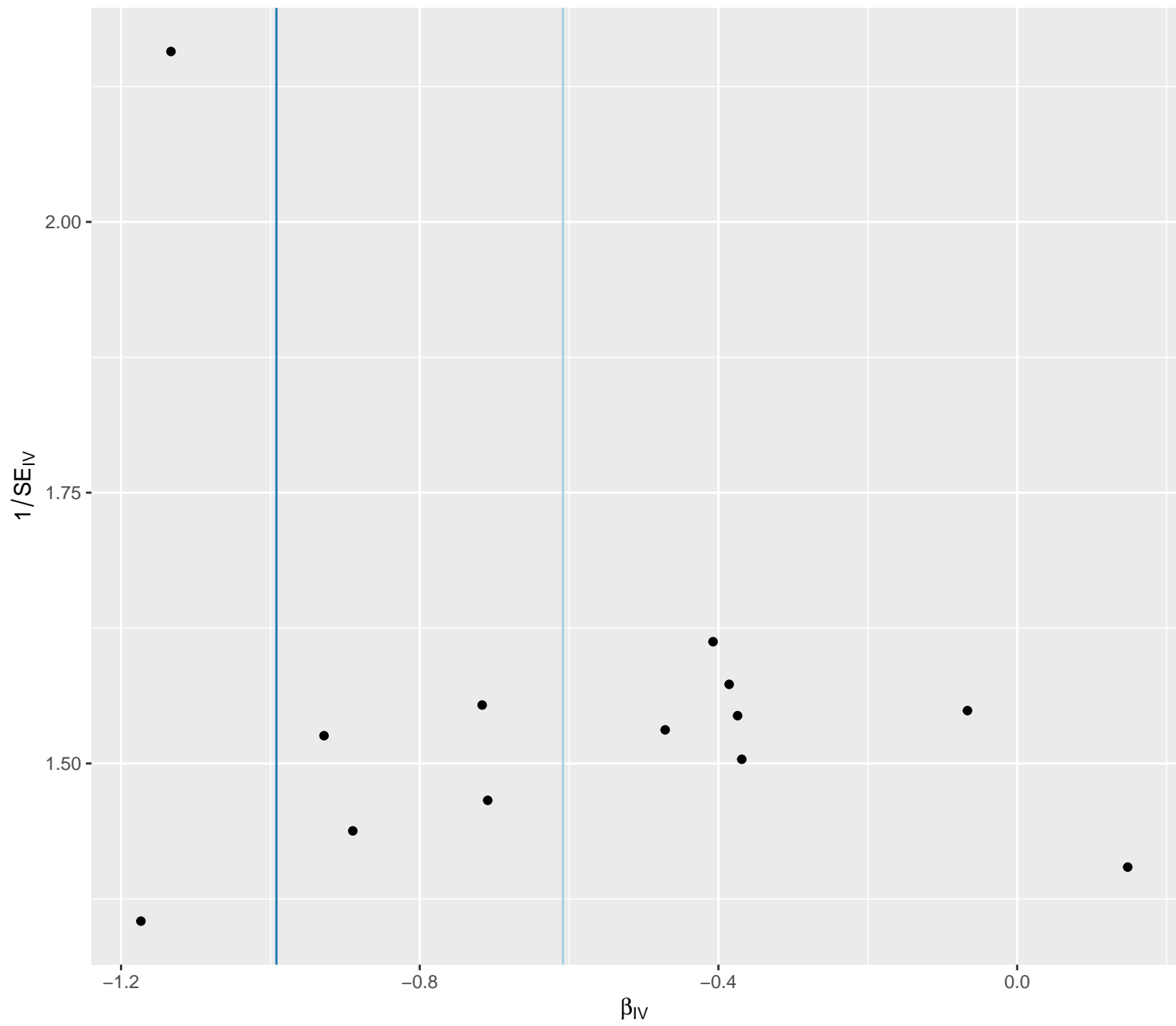

Supplement: Supplementary file 8 — Data S4: Supporting Information. [file ADB-31-e70160-s001.zip › Additional file4/Forward MR analysis/GCST90005384.pdf]

# MR Method

- Inverse variance weighted
- MR Egger

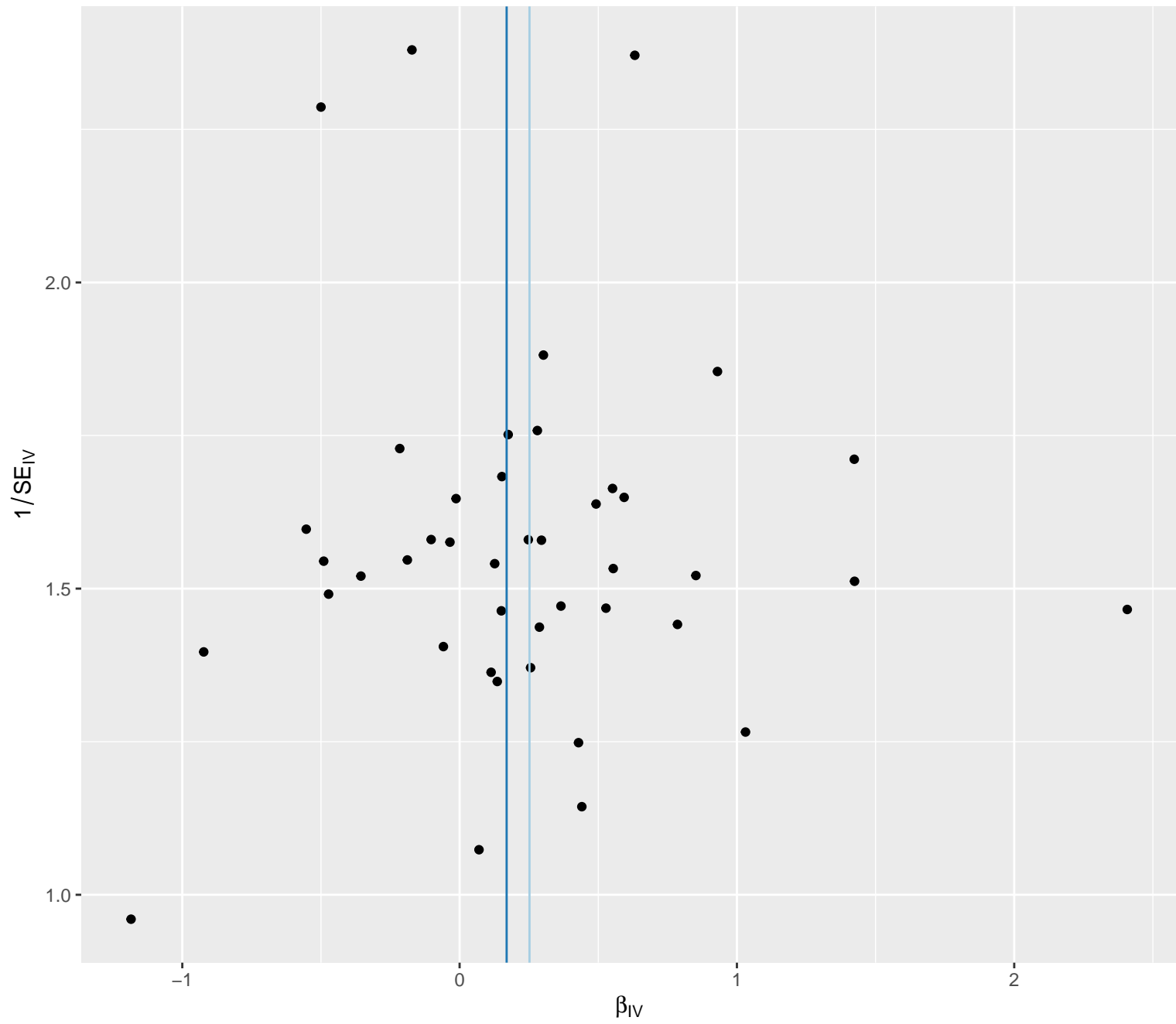

Supplement: Supplementary file 8 — Data S4: Supporting Information. [file ADB-31-e70160-s001.zip › Additional file4/Forward MR analysis/GCST90002946.pdf]
